# Prevalence of sexually risky behaviors among Mexican medical students

**DOI:** 10.1371/journal.pone.0302570

**Published:** 2024-05-06

**Authors:** Gina Stella Garcia-Romo, Glustein Pozo-Molina, Julia Reyes-Reali, Claudia Fabiola Mendez-Catala, Efrain Garrido, Adolfo Rene Mendez-Cruz, Patricia Alanis-Lopez, Maria Isabel Mendoza-Ramos, Oscar Nieto‐Yañez, Nelly Rivera-Yañez, Alexander Pedroza-Gonzalez

**Affiliations:** 1 Laboratorio de Inmunología, Unidad de Morfología y Función, Facultad de Estudios Superiores Iztacala, Universidad Nacional Autónoma de México, México City, México; 2 Carrera de Médico Cirujano, Facultad de Estudios Superiores Iztacala, Universidad Nacional Autónoma de México, México City, México; 3 Laboratorio de Genética y Oncología Molecular, Laboratorio, Edificio A4, Carrera de Médico Cirujano, Facultad de Estudios Superiores Iztacala, Universidad Nacional Autónoma de México, Tlalnepantla, Estado de México, México; 4 División de Investigación y Posgrado, Facultad de Estudios Superiores Iztacala, Universidad Nacional Autónoma de México, Tlalnepantla, México; 5 Departamento de Genética y Biología Molecular, CINVESTAV-IPN, Ciudad de México, México; 6 Hospital de Gineco Obstetricia No. 3 del Centro Médico Nacional la Raza del Instituto Mexicano del Seguro Social, Ciudad de México, México; University of Udine: Universita degli Studi di Udine, ITALY

## Abstract

University students are at high risk of sexually transmitted infections due to the lack of adequate sexual education, as well as multiple associated factors, which lead to risky sexual practices. It is important to update data about sexual behaviors to identify the main factors associated with sexually risky behaviors. The present study aimed to evaluate the current prevalence of sexually risky practices in medical students. A cross-sectional study was conducted among medical students through an anonymous self-administered online questionnaire including demographic characteristics and sexual behaviors. We used descriptive statistics and multivariable regression to analyze the data collected. A total of 1520 undergraduate medical students aged between 18 and 28 years old were included in the study. Sixty percent of the students were sexually active with a higher proportion in men (70%), likewise, they had an earlier sexual debut (16.5 vs 16.9 years old), and a greater number of lifetime sexual partners than women (3.8 vs 2.2). The main sexual activity in both groups was vaginal sex with high use of condoms (75%), however, most of them (67%) reported having unprotected oral sex. Logistic regression analysis showed that condomless sex was associated with having oral sex, anal sex, and being female. The findings of this study showed that medical university students are involved in risky sexual behaviors, the major risk factor was unprotected oral sex. Based on these results, we recommended designing interventions to improve sexual education and preventive approaches from early stages such as in middle school students to mitigate sexually transmitted infections among medical university students.

## Introduction

Sexually transmitted infections (STIs) are an important public health problem worldwide because they directly impact sexual and reproductive health through stigmatization, infertility, cancers, and pregnancy. STIs are caused by a broad spectrum of bacteria, fungi, viruses, and parasites that are transmitted through sexual contact, including vaginal, anal, and oral sex. Although sexual transmission plays a primary role, some STIs can spread differently, such as from mother to child during pregnancy, childbirth, breastfeeding, or through blood products and tissue transfer [1]. STIs affect individuals of all ages, however, young people between ages 15–24 (adolescents and young adults) account for almost half of new sexually transmitted infections according to the Centers for Disease Control and Prevention (CDC) [2]. In Mexico, this group accounted for 25% of new cases of STIs in 2022 [3]. Part of the increased risk for STIs among adolescents is attributable to biological factors like hormonal changes, the lack of immunity against the pathogens that cause them, and an increased risk for physically traumatic sex [4–6]. Furthermore, this group of the population is more likely to practice risky sexual behaviors, which include, having sex with multiple partners, having unprotected sex, having sexual intercourse with strangers, and having intoxicated sex, because there are various factors such as lack of adequate sexual education, socio-economic status, family structure, parental education, smoking, illegal drugs or alcohol use, low self-esteem, depression, anxiety, impulsiveness, delinquent behaviors and as well as social norms, that affect their knowledge about STIs, their consequences and prevention [7–9]. Although undergraduate students in the health area have a greater knowledge of sexually transmitted diseases than students of other disciplines, it has been observed that both groups have deficient knowledge in this regard [10, 11]. In line with these facts, we recently reported a high incidence of HPV infection in a cohort of female health science university students [12]. Importantly, the presence of high-risk HPV was dominant, and it was associated with early age of sexual debut, an increasing number of sexual partners, and a low percentage of individuals with serum antibodies against HPV. These observations indicate that there are multiple risk factors present not only for HPV infection but also for various STIs in the university population. Given this concern, the present study was aimed to update and ascertain trends in sexual behavior as an indication of the likely risk of the spread of STIs among medical university students.

## Materials and methods

An institution-based descriptive cross-sectional study was conducted among medical students of Facultad de Estudios Superiores Iztacala (FESI) [School of Higher Studies Iztacala] of the Universidad Nacional Autónoma de México (UNAM) [National Autonomous University of Mexico]. The study was conducted from November 20, 2021, to December 20, 2022, through an anonymous web-based questionnaire. The survey included questions about demographic characteristics like sex, age, and sexual orientation. Based on the national survey on sexual and gender diversity carried out in 2021 by the National Institute of Statistics and Geography (INEGI) [13], in Mexico the main sexual preferences are heterosexual (95.2%), bisexual (2.5%), homosexual (1.8%), and others (0.5%). According to the above, for the study we only considered the three main sexual orientations. In addition, sexual behaviors were assessed, including sexual activity, age of sexual debut, number of lifetime sexual partners, and the use of condoms. All the items were designed according to previous scientific literature, and a pilot study was conducted on 30 students to validate and improve the questionnaire. The items of the questionnaire are depicted in S1 Table. The raw responses from the surveys are in S3 Table.

The study was approved by the ethics review board of the School of Higher Studies Iztacala, UNAM. The students were informed of the study in classes and the informed consent statement was embedded in the survey instructions, those who decided to participate answered the anonymous online questionnaire of 10 items. The study participants were university students aged between 18 and 28 years old. The survey was designed as a self-administered questionnaire which was converted to a web-based Moodle platform. Participation was voluntary, and the participants’ anonymity was ensured.

Data collected were transferred from the Moodle platform to Excel and GraphPad software for analysis. Data from women and men were analyzed separately and compared with each other. The differences between groups were analyzed depending on the distribution by a t-test or by the Mann-Whitney U test. Socio-demographic characteristics and sexual behaviors were calculated as percentages. Chi-square test was used to evaluate differences in proportions. Multiple logistic regression analysis was performed with unprotected sex (non-condom use at any type of sexual encounter including vaginal, anal, or oral sex) as the dependent variable. All the statistical analysis was carried out using GraphPad Prism Software version 9. *P*-values less than 0.05 were considered statistically significant (*P<0.05; **P<0.01; ***P<0.001).

## Results

A total of 1703 undergraduate students answered the survey, 118 surveys were eliminated for having incomplete or inconsistent responses, and 65 because the students were under 18 years old. Therefore, 1520 surveys were included in the study of which 72% were from women and 28% were from men. These proportions reflect those observed in the student population in the last years of the medical career at FESI (S2 Table). The mean age of the subjects at enrollment was around 19 years, and most of the individuals were heterosexual. The proportion of homosexual and bisexual students was low in both groups (Table 1).

**Table 1:** Demographic and sexual behavior characteristics of medical students.

	Women	Men	P value
Sex	1100 (72%)	420(28%)	
Age	18.9 ± 1.2 (18–28)	19 ± 1.4 (18–27)	.351^1^
Heterosexual	1012 (92%)	376 (90%)	.125^2^
Homosexual	16 (1.5%)	29 (6.9%)	< .001^2^
Bisexual	72 (6.5%)	15 (3.6%)	.025^2^
Sexually active	598 (54.3%)	293 (69.7%)	< .001^2^
Sexual debut	16.9 ± 1.4 (13–24)	16.5 ± 1.4 (12–20)	.001^*1*^
Number of lifetime partners	2.2 ± 1.9 (1–18)	3.8 ± 4 (1–30)	< .001^*1*^
Early sexual debut	16.5%	25.9%	< .001

^1^Mann Whitney test (two-tailed)

^2^Chi-square test (two-sided). The adjusted P-values less than 0.00555 were considered statistically significant. Data are reported as the number of individuals and percentages or mean ± sd and range.

**Fig 1:**
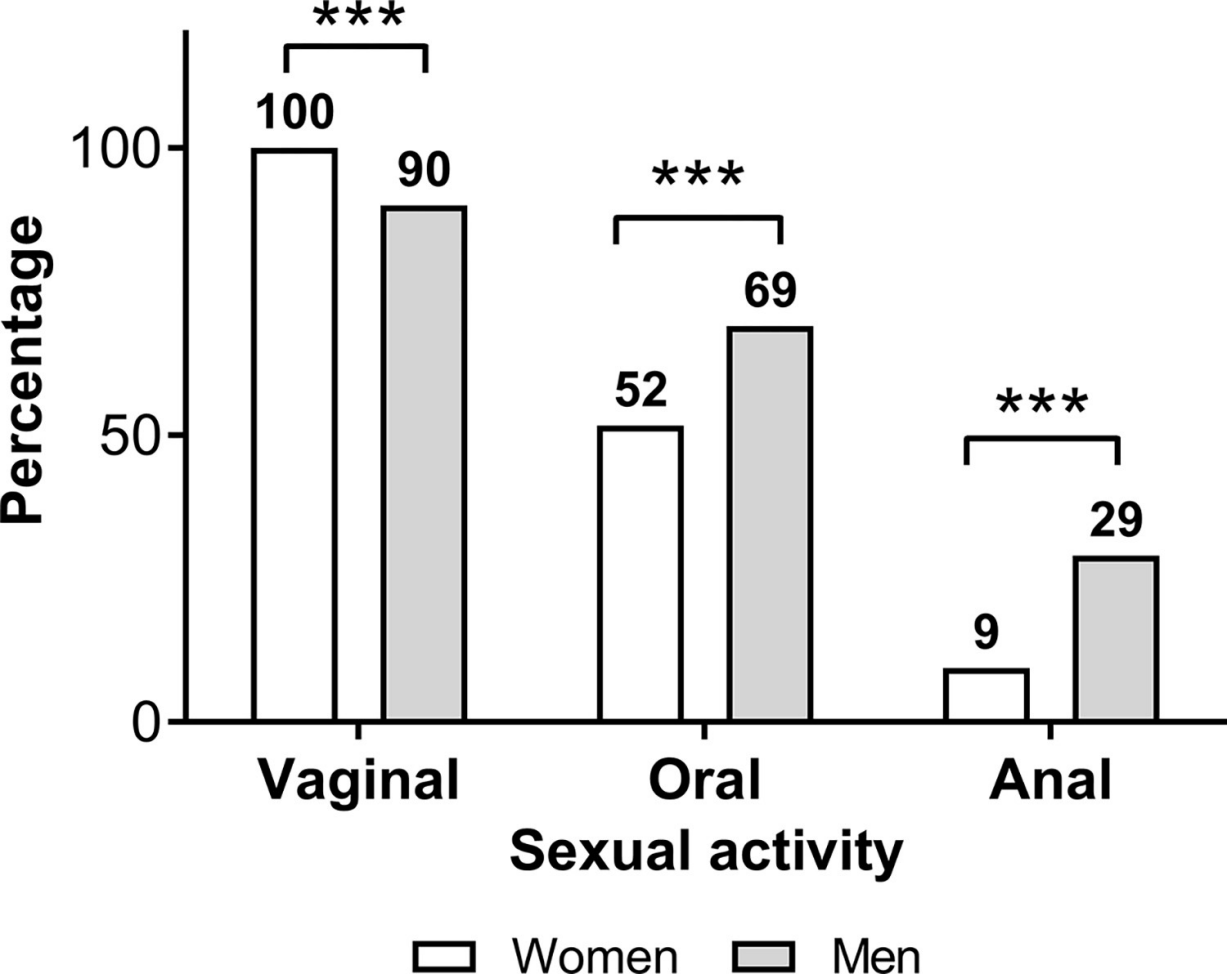
Proportions of sexual practices among sex-active students. The percentage of vaginal, oral, and anal sex in students who reported having had sexual activity. The data were analyzed usingChi-square exact test. *P*-values less than 0.05 were considered statistically significant (***P<0.001).

**Fig 2:**
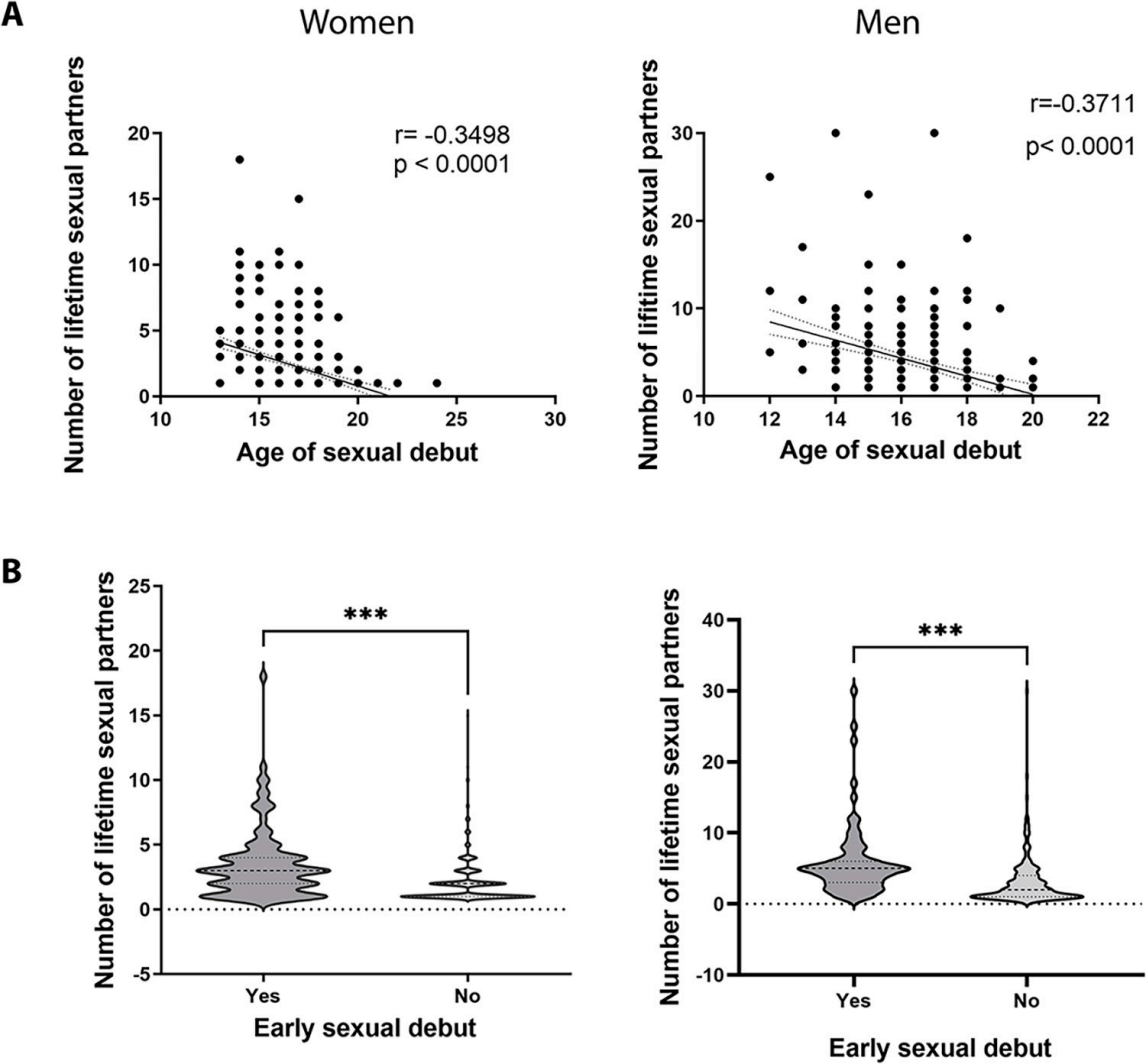
Influence of the age of sexual debut in the number of sexual partners. The timing of sexual debut was inversely correlated with the number of lifetime sexual partners for both women and men (A), Spearman correlation analysis. (B) Mann Whitney test shows that early sexual debut impact significantly the number of lifetime sexual partners in both women and men students (***P<0.001).

In the study population, 891 (58%) students reported having had some sexual activity of any kind, whether vaginal, oral, or anal. About half (54%) of the females were sexually active, a proportion significantly lower than that observed for men with almost 70% (Table 1). Among the students that had been sexually active, the main practice was vaginal intercourse. 100% of the female respondents who indicated sexual activity, reported vaginal sex as the main practice (but not the only kind); while for men, just 90% indicated this practice, showing a statistically significant difference (Fig 1). The following practice was oral sex in both groups, and the less frequent was anal sex. In both cases, men have a statistically significant higher proportion than women, for oral sex men reported having 67.7% compared with 51.6% in women. For anal sex, 28.7% of men have reported this activity, but only 9.4% of women referred to having anal sex (Fig 1). In addition to the different proportions of sexual activity, women had about 5-month delay in the age of sexual debut at 16.9 years in comparison with men at 16.5 years (*P* = 0.0016). The timing of sexual debut was inversely correlated with the number of lifetime sexual partners for both women and men (Fig 2A). According to the distribution of reported ages at first sexual activity in the population analyzed, we used the first third percentile to establish an early sexual debut, which results at or before age 15, which was in agreement with some previous studies [14–16]. Of note, in sexually active men a proportion of 25.9% had an early sexual debut, while women had only 16.5% (Table 1). Importantly, the number of lifetime sexual partners was significantly higher in those individuals who had an early sexual debut (Fig 2B). Overall, the number of lifetime sexual partners was significantly higher in males with an average of 3.8 sexual partners ranging from 1 to 30, in contrast, women reported only an average of 2.2 with a range from 1 to 18 (P < 0.0001, Table 1).

**Fig 3:**
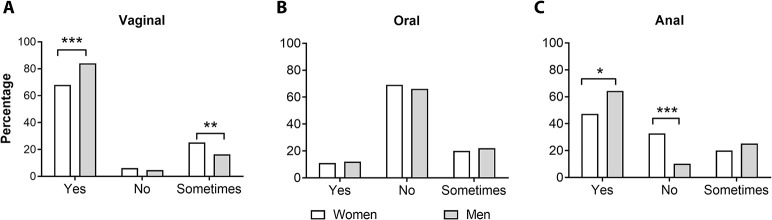
Condom use in medical students. Percentages of condom use during vaginal (A), oral (B), and anal sex (C). Responses were categorized as yes/no or sometimes and compared between both groups by Chi-square t test. *P*-values less than 0.05 were considered statistically significant (*P<0.05; **P<0.01; ***P<0.001).

Regarding preventive measures in sexual practice, an important proportion of sexually active students reported the use of condoms during vaginal intercourse, being higher in men than in women, 83.6% in men versus 67.7% in women (Fig 3A). Only a minor percentage of the students reported having unprotected vaginal sex, in the case of men the percentage was 4.8, while in women it was 6.4%. Twenty-five percent of women answered that they sometimes use condoms during vaginal sex, for men this proportion was significantly lower at 16% (*P* = 0.004, Fig 3A). Oral sex was the second most frequent sexual activity reported in the surveys, importantly, the use of condoms was minimal. Most of the students reported having unprotected oral sex (Fig 3B), 69% of women and 66% of men. Regarding anal sex, male students reported a higher percentage of condom use at 64.4%, which was statistically significantly higher than the proportion of females with 47.3% (*P* = 0.022, Fig 3C). Among students who had anal sex only 10% of men had unprotected sex, in contrast, women reported a proportion of 33% (*P* = 0.0001).

Multiple logistic regression analysis exploring risk factors for unprotected sex showed significant association with three factors: to be female (AOR 1.97, 95% CI: 1.25–3.15), to have anal sex (AOR 6.28, 95% CI: 2.59–18.08), and to have oral sex (AOR 29.66, 95% CI: 19.92–45.13), (Table 2).

**Table 2:** Associations with unprotected sex among university students.

Variable	β estimates (95% Cl)	Standard error	AOR (95% Cl)	Wald chi-square: χ^2^ = Z^2^	P value
*Sex* Male Female	Reference 0.67 (0.22, 1.15)	0.23	1.97 (1.25, 3.15)	8.48	.004
*Sexual orientation* Heterosexual Homosexual Bisexual	Reference 1.3 (-0.33, 3.14) -0.08 (-0.92, 0.8)	0.87 0.44	3.7 (0.72, 23.16) 0.92 (0.39, 2.23)	2.23 0.03	.132 .86
Early sexual debut	0.13 (-0.39, 0.66)	0.27	1.15 (0.68, 1.94)	0.23	.61
*Number of lifetime sexual partners* Less than 5 More than 5	Reference -0.002 (-0.75, 0.78)	0.39	0.99 (0.47, 2.19)	2.62	.99
Sexual activity Vaginal Anal Oral	1.02 (-0.57, 2.58) 1.83 (0.95, 2.89) 3.39 (2.99, 3.81)	0.79 0.49 0.2	2.74 (0.56, 13.19) 6.28 (2.59, 18.08) 29.66 (19.92, 45.13)	1.66 13.94 287.3	0.19 < 0.001 < 0.001

Multiple logistic regression analysis was performed with unprotected sex (non-condom use at any type of sexual encounter including vaginal, anal, or oral sex) as the dependent variable.

## Discussion

Sexually transmitted infections are an important public health problem since they can have devastating consequences, especially those of viral origin that can cause diseases such as cancer or acquired immunodeficiency syndrome (AIDS). People aged 15–24 years continue to be one of the main groups affected by these infections [2], high-risk sexual behaviors among young adults are key risk factors for STIs and unplanned pregnancies. University students are in this group population, in our study, the range of age was 18–28 with a median of 19 years old, and based on a previous report this particular population has an elevated incidence of HPV infection reflecting the susceptibility to STI [12]. Overall, in the present work, almost 60% of the students were sexually active, with men standing out with 70%, which indicates that depending on gender there is a different sexual activity and therefore men have a greater exposure to sexually transmitted infections. This was associated with an earlier sexual debut and a higher number of lifetime sexual partners in men. According to several reports, the proportion of sexually active students is variable in different regions, for example in China this proportion is low, ranging between 13–18% [17]; in Africa, the proportions are reported between 20 to 40% [18, 19]; in others regions such as Ireland and Jamaica the proportions are among the highest with a range of 75 to 85% [20, 21]; in this context, our cohort stands between the populations with higher proportions of sexually active young people, which is in agreement with a previous study conducted in the city of Queretaro in Mexico where the percentage of students with sex activity was 89% [22]. Although the great variability in the prevalence of sexual activity in different regions, the overall proportion in men is greater than that of the female population. A similar situation was observed with the age of sexual initiation, in our study men had a minor age at sexual debut than women, and 25% of men had what is considered an early sexual initiation in comparison with only 16% of female students. In agreement with several reports, we observed a lower prevalence of early sexual initiation among women than men, of note, in a study that analyzed 50 countries the prevalence reported in America was the highest with pooled estimates of 11% for females and 18% for males, which are slightly lower than those observed in our cohort. However, Mexico was not included [23]. Furthermore, other factors that have a significant influence on sexual initiation are cultural, religious, social, psychological, sex education, drug use, and family factors including single-parent family structure, parent-adolescent relationship, and the level of parental monitoring [23–25].

We found that the main sexual practice was vaginal intercourse, followed by oral sex, and a low frequency of anal sex. In several reports, vaginal sex is the main sexual practice among university students, and the proportions of anal and oral sex are diverse [18, 26]. Importantly, the practice of unsafe sex in college students is common, particularly, inconsistent condom use becomes one of the most relevant risk factors for STIs [27]. For vaginal sex we observed that only a minority in both women and men reported unprotected sex, reflecting preventive behavior against STIs or possible pregnancy. Female students had significantly lower use of condoms in vaginal sex probably because they tend to have a more stable sexual partner and develop a feeling of trust as the time of the relationship passes according to previous reports [27, 28], unlike men who reported a greater number of sexual partners. Alarmingly, we observe that a high percentage of the students surveyed do not use a condom during oral sex, with no significant differences among females and males. This finding is very relevant because several studies have indicated that oral sex in young people has been increasing over time [29–31], the overall proportion of oral sex in our study was about 60%, which is similar to those observed in other reports [32, 33]. Of note, men had a higher proportion (67.7% vs. 51.6% in women), furthermore, according to some reports females are more likely to have only one oral sex partner in comparison with men that have two or more [32, 33]. The main reason that has been associated with the lack of condom use during oral sex is the deficient knowledge about the transmission of STIs by this practice [34], which in part could be due to the priority given in school-based sex education to the reproductive sex and little attention to STIs by other sexual practices than vaginal intercourse. As a result of poor sex education, many adolescents have the general perception that oral sex is low risk, increasing the potential transmission of STIs by this practice. Even though oral sex is less risky than vaginal and anal sex, many infectious diseases can be transmitted by this such as herpes, gonorrhea, chlamydia, syphilis, HIV, and human papillomavirus [35].

This study provides an updated analysis of the main sexual risk practices in the medical student population. However, it has some limitations, one is that it only focuses on a very particular population of students, and it is necessary to extend the analysis to other student populations in the health area such as nurses, but also to other areas no related, and especially to young people who do not have access to university education. Furthermore, as in previous studies [9], it would be advisable to design interventions to improve sexuality education and preventive approaches from early stages, such as in middle school students. The study only analyzed the main populations of sexual orientation (heterosexual, bisexual, and homosexual), but not the minority additional sexual groups. Another limitation of the study is that we did not analyze the possible reasons why students do not use a condom during oral sex. The main cause is likely poor information about the risks associated with said practice, but perhaps other factors should be considered for preventive strategies. For example, we did not ask about the relationship status in our assessment, including monogamous couples in the outcome of "unprotected sex", which could potentially affect the accuracy of our results. To address some of these limitations, we plan to conduct further studies considering the stratification of the data based on relationship status, investigate the reasons for the lack of condom use during oral sex, and to extend the analysis to other young adult populations.

## Conclusions

In our study, we observed that male medical university students in the Mexico City Metropolitan Area were more sexually active and had more risky sexual behaviors than female students. Importantly, this population showed a preventive behavior for vaginal sex by a high rate of condom use, however, there was an important breach in oral sex, which was the second most frequent sexual practice and therefore constitutes an important route of possible transmission of sexual infectious diseases. According to a recent study the lack of sexual education, the perception that oral sex is low or no risk, decreased pleasure, and no pregnancy risk are the main reasons to have unprotected oral sex [34]. Therefore, there is an important need for improvements in sexual education so that it includes not only what is referred to vaginal practice, but also the risks associated with oral sex and other practices. Particularly for medical students, sexual education in various subjects should be reinforced to guarantee a better understanding that could be reflected in their sexual practices. It is also important to design and establish information campaigns that are not only for the university population but that are inclusive for all youth regardless of their academic and social status. Sexual education must be accompanied by education on alcohol and drug consumption. Likewise, parenteral counseling programs are important to promote supervision and communication between parents and adolescents about healthy sexual practices and the effective use of condoms. All these educative programs should begin at the onset of puberty and continue to be reinforced in young adults.

Other preventive approaches for inhibiting the acquisition of STIs are also necessary; developing vaccines should be a priority, but making existing vaccines accessible is an urgent obligation of the health services, for example, the HPV vaccine should be applied not only to girls but also to boys.

## Supporting information

S1 Table Questionnaire items. (PDF)

S2 Table Number of medical students enrolled at FESI from 2016 to 2022. (PDF)

S3 Table Survey’s responses. (PDF)
