# Supplementary material for: Prevalence of sexually risky behaviors among Mexican medical students
Source: PLoS One. 2024 May 6;19(5):e0302570. doi: 10.1371/journal.pone.0302570 (PMC11073697; doi:10.1371/journal.pone.0302570)
Supplement: S1 Table — (PDF) [file pone.0302570.s001.pdf]

S1 Table. Questionnaire items

Indicator	Item	Answers
1. Student category	Are you a medical student	Yes/no
2. Sex	What is your sex?	Female/Male
3. Age	How old are you?	Open answer
4. Sexual orientation	Are you heterosexual, homosexual, or bisexual?	Heterosexual/homosexual/bisexual
5. Sexual activity and sexual debut	At what age did you start having sex? If you have never had sex, answer 0	0 for no sex activity<br>Open answer for sexual debut
6. Number of lifetime sexual partners	During your life, how many people have you had sex	Open answer
7. Sexual practice	What kind of sexual relations do you practice?	Vaginal/oral/anal/not applicable
8. Condom use	Do you use a condom during vaginal intercourse?	Yes/no/sometimes/not applicable
9 Condom use	Do you use a condom during oral intercourse?	Yes/no/sometimes/not applicable
10. Condom use	Do you use a condom during anal intercourse?	Yes/no/sometimes/not applicable
