# Supplementary material for: Prevalence of sexually risky behaviors among Mexican medical students
Source: PLoS One. 2024 May 6;19(5):e0302570. doi: 10.1371/journal.pone.0302570 (PMC11073697; doi:10.1371/journal.pone.0302570)
Supplement: S2 Table — (PDF) [file pone.0302570.s002.pdf]

**S2 Table. Number of medical students enrolled at FESI from 2016 to 2022.**

| Semester | Women | Men | Total |
|---|---|---|---|
| 2017-1 | 2235 (67%) | 1090 (33%) | 3325 |
| 2017-2 | 2250 (66%) | 1153 (34%) | 3403 |
| 2018-1 | 2221 (68%) | 1061(32% ) | 3282 |
| 2018-2 | 2308 (67%) | 1118 (33%) | 3426 |
| 2019-1 | 2245 (69%) | 1016 (31%) | 3261 |
| 2019-2 | 2313 (68%) | 1093 (32%) | 3406 |
| 2020-1 | 2266 (69%)) | 998 (31%) | 3264 |
| 020-2 | 2363 (69%) | 1067 (31%) | 3430 |
| 2021-1 | 2217 (70%) | 942 (30%) | 3159 |
| 2021-2 | 2357 (70%) | 1030 (30%) | 3387 |
