# Supplementary material for: Prevalence of sexually risky behaviors among Mexican medical students
Source: PLoS One. 2024 May 6;19(5):e0302570. doi: 10.1371/journal.pone.0302570 (PMC11073697; doi:10.1371/journal.pone.0302570)
Supplement: S3 Table — (PDF) [file pone.0302570.s003.pdf]

Table S3. Surver´s responses.

Sex			Sexual orientation					Sexual practice				Use of condoms during vaginal sex				Use of condoms during anal sex				Use of condoms during oral sex			
Female	Male	Age	Heterosexual	Homosexual	Bisexual	Sexual debut	Sexual partners	Vaginal	Anal	Oral	No apply	Yes	No	SOMETIMES	NO APPLY	Yes	No	SOMETIMES	Not apply	Yes	No	Sometimes	No apply
1	0	18	1	0	0	0	0	0	0	0	1	0	0	0	1	0	0	0	1	0	0	0	1
1	0	18	1	0	0	0	0	0	0	0	1	0	0	0	1	0	0	0	1	0	0	0	1
1	0	18	1	0	0	0	0	0	0	0	1	0	0	0	1	0	0	0	1	0	0	0	1
1	0	18	1	0	0	0	0	0	0	0	1	0	0	0	1	0	0	0	1	0	0	0	1
1	0	18	1	0	0	0	0	0	0	0	1	0	0	0	1	0	0	0	1	0	0	0	1
1	0	18	1	0	0	0	0	0	0	0	1	0	0	0	1	0	0	0	1	0	0	0	1
1	0	18	1	0	0	0	0	0	0	0	1	0	0	0	1	0	0	0	1	0	0	0	1
1	0	18	1	0	0	0	0	0	0	0	1	0	0	0	1	0	0	0	1	0	0	0	1
1	0	18	1	0	0	0	0	0	0	0	1	0	0	0	1	0	0	0	1	0	0	0	1
1	0	18	1	0	0	0	0	0	0	0	1	0	0	0	1	0	0	0	1	0	0	0	1
1	0	18	0	0	1	0	0	0	0	0	1	0	0	0	1	0	0	0	1	0	0	0	1
1	0	18	1	0	0	0	0	0	0	0	1	0	0	0	1	0	0	0	1	0	0	0	1
1	0	18	1	0	0	0	0	0	0	0	1	0	0	0	1	0	0	0	1	0	0	0	1
1	0	18	1	0	0	0	0	0	0	0	1	0	0	0	1	0	0	0	1	0	0	0	1
1	0	18	1	0	0	0	0	0	0	0	1	0	0	0	1	0	0	0	1	0	0	0	1
1	0	18	1	0	0	0	0	0	0	0	1	0	0	0	1	0	0	0	1	0	0	0	1
1	0	18	1	0	0	0	0	0	0	0	1	0	0	0	1	0	0	0	1	0	0	0	1
1	0	18	1	0	0	0	0	0	0	0	1	0	0	0	1	0	0	0	1	0	0	0	1
1	0	18	1	0	0	0	0	0	0	0	1	0	0	0	1	0	0	0	1	0	0	0	1
1	0	18	1	0	0	0	0	0	0	0	1	0	0	0	1	0	0	0	1	0	0	0	1
1	0	18	1	0	0	0	0	0	0	0	1	0	0	0	1	0	0	0	1	0	0	0	1
1	0	18	1	0	0	0	0	0	0	0	1	0	0	0	1	0	0	0	1	0	0	0	1
1	0	18	1	0	0	0	0	0	0	0	1	0	0	0	1	0	0	0	1	0	0	0	1
1	0	18	1	0	0	0	0	0	0	0	1	0	0	0	1	0	0	0	1	0	0	0	1
1	0	18	1	0	0	0	0	0	0	0	1	0	0	0	1	0	0	0	1	0	0	0	1
1	0	18	1	0	0	0	0	0	0	0	1	0	0	0	1	0	0	0	1	0	0	0	1
1	0	18	1	0	0	0	0	0	0	0	1	0	0	0	1	0	0	0	1	0	0	0	1
1	0	18	1	0	0	0	0	0	0	0	1	0	0	0	1	0	0	0	1	0	0	0	1
1	0	18	1	0	0	0	0	0	0	0	1	0	0	0	1	0	0	0	1	0	0	0	1
1	0	18	1	0	0	0	0	0	0	0	1	0	0	0	1	0	0	0	1	0	0	0	1
1	0	18	1	0	0	0	0	0	0	0	1	0	0	0	1	0	0	0	1	0	0	0	1
1	0	18	1	0	0	0	0	0	0	0	1	0	0	0	1	0	0	0	1	0	0	0	1
1	0	18	0	1	0	0	0	0	0	0	1	0	0	0	1	0	0	0	1	0	0	0	1
1	0	18	1	0	0	0	0	0	0	0	1	0	0	0	1	0	0	0	1	0	0	0	1
1	0	18	1	0	0	0	0	0	0	0	1	0	0	0	1	0	0	0	1	0	0	0	1
1	0	18	1	0	0	0	0	0	0	0	1	0	0	0	1	0	0	0	1	0	0	0	1
1	0	18	1	0	0	0	0	0	0	0	1	0	0	0	1	0	0	0	1	0	0	0	1
1	0	18	1	0	0	0	0	0	0	0	1	0	0	0	1	0	0	0	1	0	0	0	1
1	0	18	1	0	0	0	0	0	0	0	1	0	0	0	1	0	0	0	1	0	0	0	1
1	0	18	1	0	0	0	0	0	0	0	1	0	0	0	1	0	0	0	1	0	0	0	1
1	0	18	1	0	0	0	0	0	0	0	1	0	0	0	1	0	0	0	1	0	0	0	1
1	0	18	1	0	0	0	0	0	0	0	1	0	0	0	1	0	0	0	1	0	0	0	1
1	0	18	1	0	0	0	0	0	0	0	1	0	0	0	1	0	0	0	1	0	0	0	1
1	0	18	1	0	0	0	0	0	0	0	1	0	0	0	1	0	0	0	1	0	0	0	1
1	0	18	1	0	0	0	0	0	0	0	1	0	0	0	1	0	0	0	1	0	0	0	1
1	0	18	1	0	0	0	0	0	0	0	1	0	0	0	1	0	0	0	1	0	0	0	1
1	0	18	1	0	0	0	0	0	0	0	1	0	0	0	1	0	0	0	1	0	0	0	1
1	0	18	1	0	0	0	0	0	0	0	1	0	0	0	1	0	0	0	1	0	0	0	1

1	0	18	1	0	0	0	0	0	0	0	1	0	0	0	1	0	0	0	1	0	0	0	1
1	0	18	1	0	0	0	0	0	0	0	1	0	0	0	1	0	0	0	1	0	0	0	1
1	0	18	0	0	1	0	0	0	0	0	1	0	0	0	1	0	0	0	1	0	0	0	1
1	0	18	1	0	0	0	0	0	0	0	1	0	0	0	1	0	0	0	1	0	0	0	1
1	0	18	1	0	0	0	0	0	0	0	1	0	0	0	1	0	0	0	1	0	0	0	1
1	0	18	1	0	0	0	0	0	0	0	1	0	0	0	1	0	0	0	1	0	0	0	1
1	0	18	1	0	0	0	0	0	0	0	1	0	0	0	1	0	0	0	1	0	0	0	1
1	0	18	0	1	0	0	0	0	0	0	1	0	0	0	1	0	0	0	1	0	0	0	1
1	0	18	1	0	0	0	0	0	0	0	1	0	0	0	1	0	0	0	1	0	0	0	1
1	0	18	1	0	0	0	0	0	0	0	1	0	0	0	1	0	0	0	1	0	0	0	1
1	0	18	1	0	0	0	0	0	0	0	1	0	0	0	1	0	0	0	1	0	0	0	1
1	0	18	1	0	0	0	0	0	0	0	1	0	0	0	1	0	0	0	1	0	0	0	1
1	0	18	1	0	0	0	0	0	0	0	1	0	0	0	1	0	0	0	1	0	0	0	1
1	0	18	1	0	0	0	0	0	0	0	1	0	0	0	1	0	0	0	1	0	0	0	1
1	0	18	1	0	0	0	0	0	0	0	1	0	0	0	1	0	0	0	1	0	0	0	1
1	0	18	1	0	0	0	0	0	0	0	1	0	0	0	1	0	0	0	1	0	0	0	1
1	0	18	1	0	0	0	0	0	0	0	1	0	0	0	1	0	0	0	1	0	0	0	1
1	0	18	1	0	0	0	0	0	0	0	1	0	0	0	1	0	0	0	1	0	0	0	1
1	0	18	1	0	0	0	0	0	0	0	1	0	0	0	1	0	0	0	1	0	0	0	1
1	0	18	1	0	0	0	0	0	0	0	1	0	0	0	1	0	0	0	1	0	0	0	1
1	0	18	1	0	0	0	0	0	0	0	1	0	0	0	1	0	0	0	1	0	0	0	1
1	0	19	0	0	1	0	0	0	0	0	1	0	0	0	1	0	0	0	1	0	0	0	1
1	0	19	1	0	0	0	0	0	0	0	1	0	0	0	1	0	0	0	1	0	0	0	1
1	0	19	1	0	0	0	0	0	0	0	1	0	0	0	1	0	0	0	1	0	0	0	1
1	0	19	1	0	0	0	0	0	0	0	1	0	0	0	1	0	0	0	1	0	0	0	1
1	0	19	1	0	0	0	0	0	0	0	1	0	0	0	1	0	0	0	1	0	0	0	1
1	0	19	1	0	0	0	0	0	0	0	1	0	0	0	1	0	0	0	1	0	0	0	1
1	0	19	1	0	0	0	0	0	0	0	1	0	0	0	1	0	0	0	1	0	0	0	1
1	0	19	1	0	0	0	0	0	0	0	1	0	0	0	1	0	0	0	1	0	0	0	1
1	0	19	1	0	0	0	0	0	0	0	1	0	0	0	1	0	0	0	1	0	0	0	1
1	0	19	1	0	0	0	0	0	0	0	1	0	0	0	1	0	0	0	1	0	0	0	1
1	0	19	1	0	0	0	0	0	0	0	1	0	0	0	1	0	0	0	1	0	0	0	1
1	0	19	1	0	0	0	0	0	0	0	1	0	0	0	1	0	0	0	1	0	0	0	1
1	0	19	1	0	0	0	0	0	0	0	1	0	0	0	1	0	0	0	1	0	0	0	1
1	0	19	1	0	0	0	0	0	0	0	1	0	0	0	1	0	0	0	1	0	0	0	1
1	0	19	1	0	0	0	0	0	0	0	1	0	0	0	1	0	0	0	1	0	0	0	1
1	0	19	0	0	1	0	0	0	0	0	1	0	0	0	1	0	0	0	1	0	0	0	1
1	0	19	1	0	0	0	0	0	0	0	1	0	0	0	1	0	0	0	1	0	0	0	1
1	0	19	1	0	0	0	0	0	0	0	1	0	0	0	1	0	0	0	1	0	0	0	1
1	0	19	1	0	0	0	0	0	0	0	1	0	0	0	1	0	0	0	1	0	0	0	1
1	0	19	1	0	0	0	0	0	0	0	1	0	0	0	1	0	0	0	1	0	0	0	1
1	0	19	1	0	0	0	0	0	0	0	1	0	0	0	1	0	0	0	1	0	0	0	1
1	0	19	1	0	0	0	0	0	0	0	1	0	0	0	1	0	0	0	1	0	0	0	1
1	0	19	1	0	0	0	0	0	0	0	1	0	0	0	1	0	0	0	1	0	0	0	1
1	0	19	1	0	0	0	0	0	0	0	1	0	0	0	1	0	0	0	1	0	0	0	1
1	0	19	1	0	0	0	0	0	0	0	1	0	0	0	1	0	0	0	1	0	0	0	1
1	0	19	1	0	0	0	0	0	0	0	1	0	0	0	1	0	0	0	1	0	0	0	1
1	0	19	1	0	0	0	0	0	0	0	1	0	0	0	1	0	0	0	1	0	0	0	1
1	0	19	1	0	0	0	0	0	0	0	1	0	0	0	1	0	0	0	1	0	0	0	1
1	0	19	1	0	0	0	0	0	0	0	1	0	0	0	1	0	0	0	1	0	0	0	1

1	0	19	1	0	0	0	0	0	0	0	1	0	0	0	1	0	0	0	1	0	0	0	1
1	0	19	1	0	0	0	0	0	0	0	1	0	0	0	1	0	0	0	1	0	0	0	1
1	0	19	1	0	0	0	0	0	0	0	1	0	0	0	1	0	0	0	1	0	0	0	1
1	0	19	1	0	0	0	0	0	0	0	1	0	0	0	1	0	0	0	1	0	0	0	1
1	0	19	1	0	0	0	0	0	0	0	1	0	0	0	1	0	0	0	1	0	0	0	1
1	0	19	1	0	0	0	0	0	0	0	1	0	0	0	1	0	0	0	1	0	0	0	1
1	0	19	1	0	0	0	0	0	0	0	1	0	0	0	1	0	0	0	1	0	0	0	1
1	0	19	1	0	0	0	0	0	0	0	1	0	0	0	1	0	0	0	1	0	0	0	1
1	0	19	1	0	0	0	0	0	0	0	1	0	0	0	1	0	0	0	1	0	0	0	1
1	0	19	1	0	0	0	0	0	0	0	1	0	0	0	1	0	0	0	1	0	0	0	1
1	0	19	1	0	0	0	0	0	0	0	1	0	0	0	1	0	0	0	1	0	0	0	1
1	0	19	1	0	0	0	0	0	0	0	1	0	0	0	1	0	0	0	1	0	0	0	1
1	0	19	1	0	0	0	0	0	0	0	1	0	0	0	1	0	0	0	1	0	0	0	1
1	0	19	1	0	0	0	0	0	0	0	1	0	0	0	1	0	0	0	1	0	0	0	1
1	0	19	1	0	0	0	0	0	0	0	1	0	0	0	1	0	0	0	1	0	0	0	1
1	0	19	1	0	0	0	0	0	0	0	1	0	0	0	1	0	0	0	1	0	0	0	1
1	0	19	0	0	1	0	0	0	0	0	1	0	0	0	1	0	0	0	1	0	0	0	1
1	0	19	1	0	0	0	0	0	0	0	1	0	0	0	1	0	0	0	1	0	0	0	1
1	0	20	1	0	0	0	0	0	0	0	1	0	0	0	1	0	0	0	1	0	0	0	1
1	0	20	1	0	0	0	0	0	0	0	1	0	0	0	1	0	0	0	1	0	0	0	1
1	0	20	1	0	0	0	0	0	0	0	1	0	0	0	1	0	0	0	1	0	0	0	1
1	0	20	1	0	0	0	0	0	0	0	1	0	0	0	1	0	0	0	1	0	0	0	1
1	0	20	1	0	0	0	0	0	0	0	1	0	0	0	1	0	0	0	1	0	0	0	1
1	0	20	1	0	0	0	0	0	0	0	1	0	0	0	1	0	0	0	1	0	0	0	1
1	0	20	1	0	0	0	0	0	0	0	1	0	0	0	1	0	0	0	1	0	0	0	1
1	0	20	1	0	0	0	0	0	0	0	1	0	0	0	1	0	0	0	1	0	0	0	1
1	0	20	1	0	0	0	0	0	0	0	1	0	0	0	1	0	0	0	1	0	0	0	1
1	0	21	1	0	0	0	0	0	0	0	1	0	0	0	1	0	0	0	1	0	0	0	1
1	0	21	1	0	0	0	0	0	0	0	1	0	0	0	1	0	0	0	1	0	0	0	1
1	0	21	1	0	0	0	0	0	0	0	1	0	0	0	1	0	0	0	1	0	0	0	1
1	0	18	1	0	0	0	0	0	0	0	1	0	0	0	1	0	0	0	1	0	0	0	1
1	0	20	1	0	0	0	0	0	0	0	1	0	0	0	1	0	0	0	1	0	0	0	1
1	0	18	1	0	0	0	0	0	0	0	1	0	0	0	1	0	0	0	1	0	0	0	1
1	0	18	1	0	0	0	0	0	0	0	1	0	0	0	1	0	0	0	1	0	0	0	1
1	0	18	1	0	0	0	0	0	0	0	1	0	0	0	1	0	0	0	1	0	0	0	1
1	0	18	1	0	0	0	0	0	0	0	1	0	0	0	1	0	0	0	1	0	0	0	1
1	0	19	0	0	1	0	0	0	0	0	1	0	0	0	1	0	0	0	1	0	0	0	1
1	0	18	1	0	0	0	0	0	0	0	1	0	0	0	1	0	0	0	1	0	0	0	1
1	0	18	1	0	0	0	0	0	0	0	1	0	0	0	1	0	0	0	1	0	0	0	1
1	0	18	1	0	0	0	0	0	0	0	1	0	0	0	1	0	0	0	1	0	0	0	1
1	0	18	1	0	0	0	0	0	0	0	1	0	0	0	1	0	0	0	1	0	0	0	1
1	0	18	1	0	0	0	0	0	0	0	1	0	0	0	1	0	0	0	1	0	0	0	1
1	0	18	1	0	0	0	0	0	0	0	1	0	0	0	1	0	0	0	1	0	0	0	1
1	0	18	1	0	0	0	0	0	0	0	1	0	0	0	1	0	0	0	1	0	0	0	1
1	0	18	1	0	0	0	0	0	0	0	1	0	0	0	1	0	0	0	1	0	0	0	1
1	0	18	1	0	0	0	0	0	0	0	1	0	0	0	1	0	0	0	1	0	0	0	1
1	0	18	1	0	0	0	0	0	0	0	1	0	0	0	1	0	0	0	1	0	0	0	1
1	0	18	1	0	0	0	0	0	0	0	1	0	0	0	1	0	0	0	1	0	0	0	1
1	0	18	0	0	1	0	0	0	0	0	1	0	0	0	1	0	0	0	1	0	0	0	1
1	0	18	1	0	0	0	0	0	0	0	1	0	0	0	1	0	0	0	1	0	0	0	1

1	0	19	1	0	0	0	0	0	0	0	1	0	0	0	1	0	0	0	1	0	0	0	1
1	0	19	1	0	0	0	0	0	0	0	1	0	0	0	1	0	0	0	1	0	0	0	1
1	0	19	1	0	0	0	0	0	0	0	1	0	0	0	1	0	0	0	1	0	0	0	1
1	0	19	1	0	0	0	0	0	0	0	1	0	0	0	1	0	0	0	1	0	0	0	1
1	0	20	1	0	0	0	0	0	0	0	1	0	0	0	1	0	0	0	1	0	0	0	1
1	0	20	1	0	0	0	0	0	0	0	1	0	0	0	1	0	0	0	1	0	0	0	1
1	0	20	1	0	0	0	0	0	0	0	1	0	0	0	1	0	0	0	1	0	0	0	1
1	0	21	1	0	0	0	0	0	0	0	1	0	0	0	1	0	0	0	1	0	0	0	1
1	0	18	1	0	0	0	0	0	0	0	1	0	0	0	1	0	0	0	1	0	0	0	1
1	0	18	1	0	0	0	0	0	0	0	1	0	0	0	1	0	0	0	1	0	0	0	1
1	0	18	1	0	0	0	0	0	0	0	1	0	0	0	1	0	0	0	1	0	0	0	1
1	0	18	1	0	0	0	0	0	0	0	1	0	0	0	1	0	0	0	1	0	0	0	1
1	0	18	0	0	1	0	0	0	0	0	1	0	0	0	1	0	0	0	1	0	0	0	1
1	0	18	1	0	0	0	0	0	0	0	1	0	0	0	1	0	0	0	1	0	0	0	1
1	0	18	1	0	0	0	0	0	0	0	1	0	0	0	1	0	0	0	1	0	0	0	1
1	0	18	1	0	0	0	0	0	0	0	1	0	0	0	1	0	0	0	1	0	0	0	1
1	0	18	1	0	0	0	0	0	0	0	1	0	0	0	1	0	0	0	1	0	0	0	1
1	0	18	1	0	0	0	0	0	0	0	1	0	0	0	1	0	0	0	1	0	0	0	1
1	0	18	1	0	0	0	0	0	0	0	1	0	0	0	1	0	0	0	1	0	0	0	1
1	0	18	1	0	0	0	0	0	0	0	1	0	0	0	1	0	0	0	1	0	0	0	1
1	0	18	1	0	0	0	0	0	0	0	1	0	0	0	1	0	0	0	1	0	0	0	1
1	0	18	1	0	0	0	0	0	0	0	1	0	0	0	1	0	0	0	1	0	0	0	1
1	0	18	1	0	0	0	0	0	0	0	1	0	0	0	1	0	0	0	1	0	0	0	1
1	0	18	1	0	0	0	0	0	0	0	1	0	0	0	1	0	0	0	1	0	0	0	1
1	0	18	1	0	0	0	0	0	0	0	1	0	0	0	1	0	0	0	1	0	0	0	1
1	0	18	1	0	0	0	0	0	0	0	1	0	0	0	1	0	0	0	1	0	0	0	1
1	0	19	1	0	0	0	0	0	0	0	1	0	0	0	1	0	0	0	1	0	0	0	1
1	0	19	1	0	0	0	0	0	0	0	1	0	0	0	1	0	0	0	1	0	0	0	1
1	0	19	1	0	0	0	0	0	0	0	1	0	0	0	1	0	0	0	1	0	0	0	1
1	0	19	1	0	0	0	0	0	0	0	1	0	0	0	1	0	0	0	1	0	0	0	1
1	0	19	1	0	0	0	0	0	0	0	1	0	0	0	1	0	0	0	1	0	0	0	1
1	0	19	1	0	0	0	0	0	0	0	1	0	0	0	1	0	0	0	1	0	0	0	1
1	0	19	0	0	1	0	0	0	0	0	1	0	0	0	1	0	0	0	1	0	0	0	1
1	0	19	1	0	0	0	0	0	0	0	1	0	0	0	1	0	0	0	1	0	0	0	1
1	0	19	1	0	0	0	0	0	0	0	1	0	0	0	1	0	0	0	1	0	0	0	1
1	0	19	1	0	0	0	0	0	0	0	1	0	0	0	1	0	0	0	1	0	0	0	1
1	0	20	1	0	0	0	0	0	0	0	1	0	0	0	1	0	0	0	1	0	0	0	1
1	0	20	1	0	0	0	0	0	0	0	1	0	0	0	1	0	0	0	1	0	0	0	1
1	0	20	0	0	1	0	0	0	0	0	1	0	0	0	1	0	0	0	1	0	0	0	1
1	0	20	1	0	0	0	0	0	0	0	1	0	0	0	1	0	0	0	1	0	0	0	1
1	0	20	1	0	0	0	0	0	0	0	1	0	0	0	1	0	0	0	1	0	0	0	1
1	0	18	1	0	0	0	0	0	0	0	1	0	0	0	1	0	0	0	1	0	0	0	1
1	0	18	0	0	1	0	0	0	0	0	1	0	0	0	1	0	0	0	1	0	0	0	1
1	0	18	1	0	0	0	0	0	0	0	1	0	0	0	1	0	0	0	1	0	0	0	1
1	0	18	1	0	0	0	0	0	0	0	1	0	0	0	1	0	0	0	1	0	0	0	1
1	0	18	1	0	0	0	0	0	0	0	1	0	0	0	1	0	0	0	1	0	0	0	1
1	0	18	1	0	0	0	0	0	0	0	1	0	0	0	1	0	0	0	1	0	0	0	1
1	0	18	1	0	0	0	0	0	0	0	1	0	0	0	1	0	0	0	1	0	0	0	1
1	0	18	1	0	0	0	0	0	0	0	1	0	0	0	1	0	0	0	1	0	0	0	1
1	0	18	1	0	0	0	0	0	0	0	1	0	0	0	1	0	0	0	1	0	0	0	1

1	0	18	1	0	0	0	0	0	0	0	1	0	0	0	1	0	0	0	1	0	0	0	1
1	0	18	1	0	0	0	0	0	0	0	1	0	0	0	1	0	0	0	1	0	0	0	1
1	0	18	1	0	0	0	0	0	0	0	1	0	0	0	1	0	0	0	1	0	0	0	1
1	0	18	1	0	0	0	0	0	0	0	1	0	0	0	1	0	0	0	1	0	0	0	1
1	0	18	1	0	0	0	0	0	0	0	1	0	0	0	1	0	0	0	1	0	0	0	1
1	0	18	1	0	0	0	0	0	0	0	1	0	0	0	1	0	0	0	1	0	0	0	1
1	0	18	1	0	0	0	0	0	0	0	1	0	0	0	1	0	0	0	1	0	0	0	1
1	0	18	1	0	0	0	0	0	0	0	1	0	0	0	1	0	0	0	1	0	0	0	1
1	0	18	1	0	0	0	0	0	0	0	1	0	0	0	1	0	0	0	1	0	0	0	1
1	0	18	1	0	0	0	0	0	0	0	1	0	0	0	1	0	0	0	1	0	0	0	1
1	0	18	1	0	0	0	0	0	0	0	1	0	0	0	1	0	0	0	1	0	0	0	1
1	0	18	1	0	0	0	0	0	0	0	1	0	0	0	1	0	0	0	1	0	0	0	1
1	0	18	1	0	0	0	0	0	0	0	1	0	0	0	1	0	0	0	1	0	0	0	1
1	0	18	1	0	0	0	0	0	0	0	1	0	0	0	1	0	0	0	1	0	0	0	1
1	0	18	1	0	0	0	0	0	0	0	1	0	0	0	1	0	0	0	1	0	0	0	1
1	0	18	1	0	0	0	0	0	0	0	1	0	0	0	1	0	0	0	1	0	0	0	1
1	0	18	1	0	0	0	0	0	0	0	1	0	0	0	1	0	0	0	1	0	0	0	1
1	0	18	1	0	0	0	0	0	0	0	1	0	0	0	1	0	0	0	1	0	0	0	1
1	0	18	1	0	0	0	0	0	0	0	1	0	0	0	1	0	0	0	1	0	0	0	1
1	0	19	1	0	0	0	0	0	0	0	1	0	0	0	1	0	0	0	1	0	0	0	1
1	0	19	1	0	0	0	0	0	0	0	1	0	0	0	1	0	0	0	1	0	0	0	1
1	0	19	1	0	0	0	0	0	0	0	1	0	0	0	1	0	0	0	1	0	0	0	1
1	0	19	1	0	0	0	0	0	0	0	1	0	0	0	1	0	0	0	1	0	0	0	1
1	0	19	1	0	0	0	0	0	0	0	1	0	0	0	1	0	0	0	1	0	0	0	1
1	0	19	1	0	0	0	0	0	0	0	1	0	0	0	1	0	0	0	1	0	0	0	1
1	0	19	1	0	0	0	0	0	0	0	1	0	0	0	1	0	0	0	1	0	0	0	1
1	0	19	1	0	0	0	0	0	0	0	1	0	0	0	1	0	0	0	1	0	0	0	1
1	0	19	0	0	1	0	0	0	0	0	1	0	0	0	1	0	0	0	1	0	0	0	1
1	0	19	1	0	0	0	0	0	0	0	1	0	0	0	1	0	0	0	1	0	0	0	1
1	0	19	1	0	0	0	0	0	0	0	1	0	0	0	1	0	0	0	1	0	0	0	1
1	0	19	1	0	0	0	0	0	0	0	1	0	0	0	1	0	0	0	1	0	0	0	1
1	0	19	1	0	0	0	0	0	0	0	1	0	0	0	1	0	0	0	1	0	0	0	1
1	0	19	1	0	0	0	0	0	0	0	1	0	0	0	1	0	0	0	1	0	0	0	1
1	0	19	1	0	0	0	0	0	0	0	1	0	0	0	1	0	0	0	1	0	0	0	1
1	0	19	1	0	0	0	0	0	0	0	1	0	0	0	1	0	0	0	1	0	0	0	1
1	0	19	1	0	0	0	0	0	0	0	1	0	0	0	1	0	0	0	1	0	0	0	1
1	0	19	1	0	0	0	0	0	0	0	1	0	0	0	1	0	0	0	1	0	0	0	1
1	0	19	1	0	0	0	0	0	0	0	1	0	0	0	1	0	0	0	1	0	0	0	1
1	0	19	1	0	0	0	0	0	0	0	1	0	0	0	1	0	0	0	1	0	0	0	1
1	0	19	1	0	0	0	0	0	0	0	1	0	0	0	1	0	0	0	1	0	0	0	1
1	0	19	1	0	0	0	0	0	0	0	1	0	0	0	1	0	0	0	1	0	0	0	1
1	0	19	0	0	1	0	0	0	0	0	1	0	0	0	1	0	0	0	1	0	0	0	1
1	0	19	1	0	0	0	0	0	0	0	1	0	0	0	1	0	0	0	1	0	0	0	1
1	0	20	1	0	0	0	0	0	0	0	1	0	0	0	1	0	0	0	1	0	0	0	1
1	0	20	1	0	0	0	0	0	0	0	1	0	0	0	1	0	0	0	1	0	0	0	1
1	0	20	1	0	0	0	0	0	0	0	1	0	0	0	1	0	0	0	1	0	0	0	1
1	0	20	1	0	0	0	0	0	0	0	1	0	0	0	1	0	0	0	1	0	0	0	1
1	0	20	1	0	0	0	0	0	0	0	1	0	0	0	1	0	0	0	1	0	0	0	1
1	0	20	1	0	0	0	0	0	0	0	1	0	0	0	1	0	0	0	1	0	0	0	1
1	0	20	1	0	0	0	0	0	0	0	1	0	0	0	1	0	0	0	1	0	0	0	1

1	0	20	1	0	0	0	0	0	0	0	1	0	0	0	1	0	0	0	1	0	0	0	1
1	0	21	1	0	0	0	0	0	0	0	1	0	0	0	1	0	0	0	1	0	0	0	1
1	0	18	1	0	0	0	0	0	0	0	1	0	0	0	1	0	0	0	1	0	0	0	1
1	0	18	1	0	0	0	0	0	0	0	1	0	0	0	1	0	0	0	1	0	0	0	1
1	0	18	1	0	0	0	0	0	0	0	1	0	0	0	1	0	0	0	1	0	0	0	1
1	0	18	1	0	0	0	0	0	0	0	1	0	0	0	1	0	0	0	1	0	0	0	1
1	0	18	1	0	0	0	0	0	0	0	1	0	0	0	1	0	0	0	1	0	0	0	1
1	0	18	1	0	0	0	0	0	0	0	1	0	0	0	1	0	0	0	1	0	0	0	1
1	0	18	1	0	0	0	0	0	0	0	1	0	0	0	1	0	0	0	1	0	0	0	1
1	0	18	1	0	0	0	0	0	0	0	1	0	0	0	1	0	0	0	1	0	0	0	1
1	0	18	1	0	0	0	0	0	0	0	1	0	0	0	1	0	0	0	1	0	0	0	1
1	0	18	1	0	0	0	0	0	0	0	1	0	0	0	1	0	0	0	1	0	0	0	1
1	0	18	1	0	0	0	0	0	0	0	1	0	0	0	1	0	0	0	1	0	0	0	1
1	0	18	1	0	0	0	0	0	0	0	1	0	0	0	1	0	0	0	1	0	0	0	1
1	0	18	1	0	0	0	0	0	0	0	1	0	0	0	1	0	0	0	1	0	0	0	1
1	0	18	1	0	0	0	0	0	0	0	1	0	0	0	1	0	0	0	1	0	0	0	1
1	0	18	1	0	0	0	0	0	0	0	1	0	0	0	1	0	0	0	1	0	0	0	1
1	0	18	1	0	0	0	0	0	0	0	1	0	0	0	1	0	0	0	1	0	0	0	1
1	0	18	1	0	0	0	0	0	0	0	1	0	0	0	1	0	0	0	1	0	0	0	1
1	0	18	1	0	0	0	0	0	0	0	1	0	0	0	1	0	0	0	1	0	0	0	1
1	0	18	0	0	1	0	0	0	0	0	1	0	0	0	1	0	0	0	1	0	0	0	1
1	0	18	1	0	0	0	0	0	0	0	1	0	0	0	1	0	0	0	1	0	0	0	1
1	0	18	1	0	0	0	0	0	0	0	1	0	0	0	1	0	0	0	1	0	0	0	1
1	0	18	1	0	0	0	0	0	0	0	1	0	0	0	1	0	0	0	1	0	0	0	1
1	0	18	1	0	0	0	0	0	0	0	1	0	0	0	1	0	0	0	1	0	0	0	1
1	0	18	1	0	0	0	0	0	0	0	1	0	0	0	1	0	0	0	1	0	0	0	1
1	0	18	1	0	0	0	0	0	0	0	1	0	0	0	1	0	0	0	1	0	0	0	1
1	0	18	1	0	0	0	0	0	0	0	1	0	0	0	1	0	0	0	1	0	0	0	1
1	0	18	1	0	0	0	0	0	0	0	1	0	0	0	1	0	0	0	1	0	0	0	1
1	0	18	1	0	0	0	0	0	0	0	1	0	0	0	1	0	0	0	1	0	0	0	1
1	0	18	1	0	0	0	0	0	0	0	1	0	0	0	1	0	0	0	1	0	0	0	1
1	0	18	1	0	0	0	0	0	0	0	1	0	0	0	1	0	0	0	1	0	0	0	1
1	0	18	1	0	0	0	0	0	0	0	1	0	0	0	1	0	0	0	1	0	0	0	1
1	0	18	1	0	0	0	0	0	0	0	1	0	0	0	1	0	0	0	1	0	0	0	1
1	0	18	1	0	0	0	0	0	0	0	1	0	0	0	1	0	0	0	1	0	0	0	1
1	0	18	1	0	0	0	0	0	0	0	1	0	0	0	1	0	0	0	1	0	0	0	1
1	0	18	1	0	0	0	0	0	0	0	1	0	0	0	1	0	0	0	1	0	0	0	1
1	0	18	1	0	0	0	0	0	0	0	1	0	0	0	1	0	0	0	1	0	0	0	1
1	0	18	1	0	0	0	0	0	0	0	1	0	0	0	1	0	0	0	1	0	0	0	1
1	0	18	1	0	0	0	0	0	0	0	1	0	0	0	1	0	0	0	1	0	0	0	1
1	0	18	1	0	0	0	0	0	0	0	1	0	0	0	1	0	0	0	1	0	0	0	1
1	0	18	1	0	0	0	0	0	0	0	1	0	0	0	1	0	0	0	1	0	0	0	1
1	0	18	1	0	0	0	0	0	0	0	1	0	0	0	1	0	0	0	1	0	0	0	1
1	0	18	1	0	0	0	0	0	0	0	1	0	0	0	1	0	0	0	1	0	0	0	1
1	0	18	1	0	0	0	0	0	0	0	1	0	0	0	1	0	0	0	1	0	0	0	1
1	0	18	1	0	0	0	0	0	0	0	1	0	0	0	1	0	0	0	1	0	0	0	1
1	0	18	1	0	0	0	0	0	0	0	1	0	0	0	1	0	0	0	1	0	0	0	1
1	0	18	1	0	0	0	0	0	0	0	1	0	0	0	1	0	0	0	1	0	0	0	1
1	0	18	1	0	0	0	0	0	0	0	1	0	0	0	1	0	0	0	1	0	0	0	1
1	0	18	1	0	0	0	0	0	0	0	1	0	0	0	1	0	0	0	1	0	0	0	1

1	0	18	1	0	0	0	0	0	0	0	1	0	0	0	1	0	0	0	1	0	0	0	1
1	0	18	1	0	0	0	0	0	0	0	1	0	0	0	1	0	0	0	1	0	0	0	1
1	0	18	1	0	0	0	0	0	0	0	1	0	0	0	1	0	0	0	1	0	0	0	1
1	0	18	1	0	0	0	0	0	0	0	1	0	0	0	1	0	0	0	1	0	0	0	1
1	0	18	1	0	0	0	0	0	0	0	1	0	0	0	1	0	0	0	1	0	0	0	1
1	0	18	1	0	0	0	0	0	0	0	1	0	0	0	1	0	0	0	1	0	0	0	1
1	0	18	1	0	0	0	0	0	0	0	1	0	0	0	1	0	0	0	1	0	0	0	1
1	0	18	1	0	0	0	0	0	0	0	1	0	0	0	1	0	0	0	1	0	0	0	1
1	0	18	1	0	0	0	0	0	0	0	1	0	0	0	1	0	0	0	1	0	0	0	1
1	0	18	1	0	0	0	0	0	0	0	1	0	0	0	1	0	0	0	1	0	0	0	1
1	0	18	1	0	0	0	0	0	0	0	1	0	0	0	1	0	0	0	1	0	0	0	1
1	0	18	1	0	0	0	0	0	0	0	1	0	0	0	1	0	0	0	1	0	0	0	1
1	0	18	1	0	0	0	0	0	0	0	1	0	0	0	1	0	0	0	1	0	0	0	1
1	0	19	1	0	0	0	0	0	0	0	1	0	0	0	1	0	0	0	1	0	0	0	1
1	0	19	0	0	1	0	0	0	0	0	1	0	0	0	1	0	0	0	1	0	0	0	1
1	0	19	1	0	0	0	0	0	0	0	1	0	0	0	1	0	0	0	1	0	0	0	1
1	0	19	1	0	0	0	0	0	0	0	1	0	0	0	1	0	0	0	1	0	0	0	1
1	0	19	1	0	0	0	0	0	0	0	1	0	0	0	1	0	0	0	1	0	0	0	1
1	0	19	0	0	1	0	0	0	0	0	1	0	0	0	1	0	0	0	1	0	0	0	1
1	0	19	0	0	1	0	0	0	0	0	1	0	0	0	1	0	0	0	1	0	0	0	1
1	0	19	1	0	0	0	0	0	0	0	1	0	0	0	1	0	0	0	1	0	0	0	1
1	0	19	1	0	0	0	0	0	0	0	1	0	0	0	1	0	0	0	1	0	0	0	1
1	0	19	1	0	0	0	0	0	0	0	1	0	0	0	1	0	0	0	1	0	0	0	1
1	0	19	0	1	0	0	0	0	0	0	1	0	0	0	1	0	0	0	1	0	0	0	1
1	0	19	1	0	0	0	0	0	0	0	1	0	0	0	1	0	0	0	1	0	0	0	1
1	0	19	1	0	0	0	0	0	0	0	1	0	0	0	1	0	0	0	1	0	0	0	1
1	0	19	1	0	0	0	0	0	0	0	1	0	0	0	1	0	0	0	1	0	0	0	1
1	0	19	1	0	0	0	0	0	0	0	1	0	0	0	1	0	0	0	1	0	0	0	1
1	0	19	1	0	0	0	0	0	0	0	1	0	0	0	1	0	0	0	1	0	0	0	1
1	0	19	1	0	0	0	0	0	0	0	1	0	0	0	1	0	0	0	1	0	0	0	1
1	0	19	1	0	0	0	0	0	0	0	1	0	0	0	1	0	0	0	1	0	0	0	1
1	0	19	1	0	0	0	0	0	0	0	1	0	0	0	1	0	0	0	1	0	0	0	1
1	0	19	1	0	0	0	0	0	0	0	1	0	0	0	1	0	0	0	1	0	0	0	1
1	0	19	1	0	0	0	0	0	0	0	1	0	0	0	1	0	0	0	1	0	0	0	1
1	0	19	1	0	0	0	0	0	0	0	1	0	0	0	1	0	0	0	1	0	0	0	1
1	0	19	1	0	0	0	0	0	0	0	1	0	0	0	1	0	0	0	1	0	0	0	1
1	0	19	0	0	1	0	0	0	0	0	1	0	0	0	1	0	0	0	1	0	0	0	1
1	0	19	1	0	0	0	0	0	0	0	1	0	0	0	1	0	0	0	1	0	0	0	1
1	0	19	1	0	0	0	0	0	0	0	1	0	0	0	1	0	0	0	1	0	0	0	1
1	0	19	1	0	0	0	0	0	0	0	1	0	0	0	1	0	0	0	1	0	0	0	1
1	0	19	1	0	0	0	0	0	0	0	1	0	0	0	1	0	0	0	1	0	0	0	1
1	0	19	1	0	0	0	0	0	0	0	1	0	0	0	1	0	0	0	1	0	0	0	1
1	0	19	1	0	0	0	0	0	0	0	1	0	0	0	1	0	0	0	1	0	0	0	1
1	0	19	1	0	0	0	0	0	0	0	1	0	0	0	1	0	0	0	1	0	0	0	1
1	0	19	1	0	0	0	0	0	0	0	1	0	0	0	1	0	0	0	1	0	0	0	1
1	0	19	1	0	0	0	0	0	0	0	1	0	0	0	1	0	0	0	1	0	0	0	1
1	0	19	1	0	0	0	0	0	0	0	1	0	0	0	1	0	0	0	1	0	0	0	1
1	0	19	1	0	0	0	0	0	0	0	1	0	0	0	1	0	0	0	1	0	0	0	1
1	0	19	1	0	0	0	0	0	0	0	1	0	0	0	1	0	0	0	1	0	0	0	1
1	0	19	1	0	0	0	0	0	0	0	1	0	0	0	1	0	0	0	1	0	0	0	1

1	0	19	1	0	0	0	0	0	0	0	1	0	0	0	1	0	0	0	1	0	0	0	1
1	0	19	1	0	0	0	0	0	0	0	1	0	0	0	1	0	0	0	1	0	0	0	1
1	0	19	1	0	0	0	0	0	0	0	1	0	0	0	1	0	0	0	1	0	0	0	1
1	0	19	1	0	0	0	0	0	0	0	1	0	0	0	1	0	0	0	1	0	0	0	1
1	0	19	1	0	0	0	0	0	0	0	1	0	0	0	1	0	0	0	1	0	0	0	1
1	0	19	1	0	0	0	0	0	0	0	1	0	0	0	1	0	0	0	1	0	0	0	1
1	0	19	1	0	0	0	0	0	0	0	1	0	0	0	1	0	0	0	1	0	0	0	1
1	0	19	1	0	0	0	0	0	0	0	1	0	0	0	1	0	0	0	1	0	0	0	1
1	0	19	1	0	0	0	0	0	0	0	1	0	0	0	1	0	0	0	1	0	0	0	1
1	0	19	1	0	0	0	0	0	0	0	1	0	0	0	1	0	0	0	1	0	0	0	1
1	0	20	1	0	0	0	0	0	0	0	1	0	0	0	1	0	0	0	1	0	0	0	1
1	0	20	1	0	0	0	0	0	0	0	1	0	0	0	1	0	0	0	1	0	0	0	1
1	0	20	1	0	0	0	0	0	0	0	1	0	0	0	1	0	0	0	1	0	0	0	1
1	0	20	1	0	0	0	0	0	0	0	1	0	0	0	1	0	0	0	1	0	0	0	1
1	0	20	1	0	0	0	0	0	0	0	1	0	0	0	1	0	0	0	1	0	0	0	1
1	0	20	0	0	1	0	0	0	0	0	1	0	0	0	1	0	0	0	1	0	0	0	1
1	0	20	1	0	0	0	0	0	0	0	1	0	0	0	1	0	0	0	1	0	0	0	1
1	0	20	1	0	0	0	0	0	0	0	1	0	0	0	1	0	0	0	1	0	0	0	1
1	0	20	0	0	1	0	0	0	0	0	1	0	0	0	1	0	0	0	1	0	0	0	1
1	0	20	1	0	0	0	0	0	0	0	1	0	0	0	1	0	0	0	1	0	0	0	1
1	0	20	1	0	0	0	0	0	0	0	1	0	0	0	1	0	0	0	1	0	0	0	1
1	0	20	0	1	0	0	0	0	0	0	1	0	0	0	1	0	0	0	1	0	0	0	1
1	0	21	0	0	1	0	0	0	0	0	1	0	0	0	1	0	0	0	1	0	0	0	1
1	0	21	1	0	0	0	0	0	0	0	1	0	0	0	1	0	0	0	1	0	0	0	1
1	0	18	1	0	0	0	0	0	0	0	1	0	0	0	1	0	0	0	1	0	0	0	1
1	0	18	1	0	0	0	0	0	0	0	1	0	0	0	1	0	0	0	1	0	0	0	1
1	0	18	1	0	0	0	0	0	0	0	1	0	0	0	1	0	0	0	1	0	0	0	1
1	0	18	1	0	0	0	0	0	0	0	1	0	0	0	1	0	0	0	1	0	0	0	1
1	0	18	1	0	0	0	0	0	0	0	1	0	0	0	1	0	0	0	1	0	0	0	1
1	0	18	1	0	0	0	0	0	0	0	1	0	0	0	1	0	0	0	1	0	0	0	1
1	0	18	1	0	0	0	0	0	0	0	1	0	0	0	1	0	0	0	1	0	0	0	1
1	0	18	1	0	0	0	0	0	0	0	1	0	0	0	1	0	0	0	1	0	0	0	1
1	0	18	1	0	0	0	0	0	0	0	1	0	0	0	1	0	0	0	1	0	0	0	1
1	0	18	1	0	0	0	0	0	0	0	1	0	0	0	1	0	0	0	1	0	0	0	1
1	0	18	1	0	0	0	0	0	0	0	1	0	0	0	1	0	0	0	1	0	0	0	1
1	0	18	1	0	0	0	0	0	0	0	1	0	0	0	1	0	0	0	1	0	0	0	1
1	0	19	1	0	0	0	0	0	0	0	1	0	0	0	1	0	0	0	1	0	0	0	1
1	0	19	1	0	0	0	0	0	0	0	1	0	0	0	1	0	0	0	1	0	0	0	1
1	0	19	1	0	0	0	0	0	0	0	1	0	0	0	1	0	0	0	1	0	0	0	1
1	0	19	1	0	0	0	0	0	0	0	1	0	0	0	1	0	0	0	1	0	0	0	1
1	0	19	1	0	0	0	0	0	0	0	1	0	0	0	1	0	0	0	1	0	0	0	1
1	0	19	1	0	0	0	0	0	0	0	1	0	0	0	1	0	0	0	1	0	0	0	1
1	0	19	0	0	1	0	0	0	0	0	1	0	0	0	1	0	0	0	1	0	0	0	1
1	0	19	1	0	0	0	0	0	0	0	1	0	0	0	1	0	0	0	1	0	0	0	1
1	0	19	1	0	0	0	0	0	0	0	1	0	0	0	1	0	0	0	1	0	0	0	1
1	0	19	1	0	0	0	0	0	0	0	1	0	0	0	1	0	0	0	1	0	0	0	1
1	0	19	1	0	0	0	0	0	0	0	1	0	0	0	1	0	0	0	1	0	0	0	1
1	0	19	1	0	0	0	0	0	0	0	1	0	0	0	1	0	0	0	1	0	0	0	1
1	0	19	1	0	0	0	0	0	0	0	1	0	0	0	1	0	0	0	1	0	0	0	1
1	0	19	1	0	0	0	0	0	0	0	1	0	0	0	1	0	0	0	1	0	0	0	1

1	0	19	1	0	0	0	0	0	0	0	1	0	0	0	1	0	0	0	1	0	0	0	1
1	0	20	1	0	0	0	0	0	0	0	1	0	0	0	1	0	0	0	1	0	0	0	1
1	0	20	1	0	0	0	0	0	0	0	1	0	0	0	1	0	0	0	1	0	0	0	1
1	0	20	1	0	0	0	0	0	0	0	1	0	0	0	1	0	0	0	1	0	0	0	1
1	0	20	1	0	0	0	0	0	0	0	1	0	0	0	1	0	0	0	1	0	0	0	1
1	0	20	1	0	0	0	0	0	0	0	1	0	0	0	1	0	0	0	1	0	0	0	1
1	0	20	1	0	0	0	0	0	0	0	1	0	0	0	1	0	0	0	1	0	0	0	1
1	0	20	1	0	0	0	0	0	0	0	1	0	0	0	1	0	0	0	1	0	0	0	1
1	0	21	0	0	1	0	0	0	0	0	1	0	0	0	1	0	0	0	1	0	0	0	1
1	0	18	1	0	0	0	0	0	0	0	1	0	0	0	1	0	0	0	1	0	0	0	1
1	0	18	1	0	0	0	0	0	0	0	1	0	0	0	1	0	0	0	1	0	0	0	1
1	0	18	1	0	0	0	0	0	0	0	1	0	0	0	1	0	0	0	1	0	0	0	1
1	0	18	1	0	0	0	0	0	0	0	1	0	0	0	1	0	0	0	1	0	0	0	1
1	0	18	1	0	0	0	0	0	0	0	1	0	0	0	1	0	0	0	1	0	0	0	1
1	0	18	0	1	0	0	0	0	0	0	1	0	0	0	1	0	0	0	1	0	0	0	1
1	0	18	1	0	0	0	0	0	0	0	1	0	0	0	1	0	0	0	1	0	0	0	1
1	0	18	1	0	0	0	0	0	0	0	1	0	0	0	1	0	0	0	1	0	0	0	1
1	0	18	1	0	0	0	0	0	0	0	1	0	0	0	1	0	0	0	1	0	0	0	1
1	0	18	1	0	0	0	0	0	0	0	1	0	0	0	1	0	0	0	1	0	0	0	1
1	0	18	1	0	0	0	0	0	0	0	1	0	0	0	1	0	0	0	1	0	0	0	1
1	0	19	0	1	0	0	0	0	0	0	1	0	0	0	1	0	0	0	1	0	0	0	1
1	0	19	1	0	0	0	0	0	0	0	1	0	0	0	1	0	0	0	1	0	0	0	1
1	0	19	1	0	0	0	0	0	0	0	1	0	0	0	1	0	0	0	1	0	0	0	1
1	0	19	1	0	0	0	0	0	0	0	1	0	0	0	1	0	0	0	1	0	0	0	1
1	0	19	1	0	0	0	0	0	0	0	1	0	0	0	1	0	0	0	1	0	0	0	1
1	0	19	1	0	0	0	0	0	0	0	1	0	0	0	1	0	0	0	1	0	0	0	1
1	0	19	1	0	0	0	0	0	0	0	1	0	0	0	1	0	0	0	1	0	0	0	1
1	0	19	1	0	0	0	0	0	0	0	1	0	0	0	1	0	0	0	1	0	0	0	1
1	0	21	1	0	0	0	0	0	0	0	1	0	0	0	1	0	0	0	1	0	0	0	1
1	0	21	1	0	0	0	0	0	0	0	1	0	0	0	1	0	0	0	1	0	0	0	1
1	0	18	1	0	0	0	0	0	0	0	1	0	0	0	1	0	0	0	1	0	0	0	1
1	0	18	1	0	0	0	0	0	0	0	1	0	0	0	1	0	0	0	1	0	0	0	1
1	0	18	1	0	0	0	0	0	0	0	1	0	0	0	1	0	0	0	1	0	0	0	1
1	0	18	1	0	0	0	0	0	0	0	1	0	0	0	1	0	0	0	1	0	0	0	1
1	0	18	1	0	0	0	0	0	0	0	1	0	0	0	1	0	0	0	1	0	0	0	1
1	0	18	1	0	0	0	0	0	0	0	1	0	0	0	1	0	0	0	1	0	0	0	1
1	0	18	1	0	0	0	0	0	0	0	1	0	0	0	1	0	0	0	1	0	0	0	1
1	0	18	1	0	0	0	0	0	0	0	1	0	0	0	1	0	0	0	1	0	0	0	1
1	0	18	0	0	1	0	0	0	0	0	1	0	0	0	1	0	0	0	1	0	0	0	1
1	0	19	1	0	0	0	0	0	0	0	1	0	0	0	1	0	0	0	1	0	0	0	1
1	0	19	1	0	0	0	0	0	0	0	1	0	0	0	1	0	0	0	1	0	0	0	1
1	0	19	1	0	0	0	0	0	0	0	1	0	0	0	1	0	0	0	1	0	0	0	1
1	0	19	1	0	0	0	0	0	0	0	1	0	0	0	1	0	0	0	1	0	0	0	1
1	0	19	1	0	0	0	0	0	0	0	1	0	0	0	1	0	0	0	1	0	0	0	1
1	0	19	1	0	0	0	0	0	0	0	1	0	0	0	1	0	0	0	1	0	0	0	1
1	0	19	1	0	0	0	0	0	0	0	1	0	0	0	1	0	0	0	1	0	0	0	1
1	0	19	1	0	0	0	0	0	0	0	1	0	0	0	1	0	0	0	1	0	0	0	1
1	0	19	1	0	0	0	0	0	0	0	1	0	0	0	1	0	0	0	1	0	0	0	1
1	0	19	0	0	1	0	0	0	0	0	1	0	0	0	1	0	0	0	1	0	0	0	1
1	0	19	1	0	0	0	0	0	0	0	1	0	0	0	1	0	0	0	1	0	0	0	1

1	0	19	1	0	0	0	0	0	0	0	1	0	0	0	1	0	0	0	1	0	0	0	1
1	0	19	1	0	0	0	0	0	0	0	1	0	0	0	1	0	0	0	1	0	0	0	1
1	0	20	1	0	0	0	0	0	0	0	1	0	0	0	1	0	0	0	1	0	0	0	1
1	0	20	1	0	0	0	0	0	0	0	1	0	0	0	1	0	0	0	1	0	0	0	1
1	0	20	1	0	0	0	0	0	0	0	1	0	0	0	1	0	0	0	1	0	0	0	1
1	0	21	1	0	0	0	0	0	0	0	1	0	0	0	1	0	0	0	1	0	0	0	1
1	0	18	1	0	0	0	0	0	0	0	1	0	0	0	1	0	0	0	1	0	0	0	1
1	0	18	1	0	0	0	0	0	0	0	1	0	0	0	1	0	0	0	1	0	0	0	1
1	0	18	1	0	0	0	0	0	0	0	1	0	0	0	1	0	0	0	1	0	0	0	1
1	0	18	1	0	0	0	0	0	0	0	1	0	0	0	1	0	0	0	1	0	0	0	1
1	0	18	1	0	0	0	0	0	0	0	1	0	0	0	1	0	0	0	1	0	0	0	1
1	0	19	1	0	0	0	0	0	0	0	1	0	0	0	1	0	0	0	1	0	0	0	1
1	0	19	0	0	1	0	0	0	0	0	1	0	0	0	1	0	0	0	1	0	0	0	1
1	0	19	1	0	0	0	0	0	0	0	1	0	0	0	1	0	0	0	1	0	0	0	1
1	0	19	1	0	0	0	0	0	0	0	1	0	0	0	1	0	0	0	1	0	0	0	1
1	0	19	1	0	0	0	0	0	0	0	1	0	0	0	1	0	0	0	1	0	0	0	1
1	0	19	1	0	0	0	0	0	0	0	1	0	0	0	1	0	0	0	1	0	0	0	1
1	0	21	1	0	0	0	0	0	0	0	1	0	0	0	1	0	0	0	1	0	0	0	1
1	0	18	1	0	0	0	0	0	0	0	1	0	0	0	1	0	0	0	1	0	0	0	1
1	0	18	1	0	0	0	0	0	0	0	1	0	0	0	1	0	0	0	1	0	0	0	1
1	0	18	1	0	0	0	0	0	0	0	1	0	0	0	1	0	0	0	1	0	0	0	1
1	0	18	1	0	0	0	0	0	0	0	1	0	0	0	1	0	0	0	1	0	0	0	1
1	0	18	1	0	0	0	0	0	0	0	1	0	0	0	1	0	0	0	1	0	0	0	1
1	0	18	1	0	0	0	0	0	0	0	1	0	0	0	1	0	0	0	1	0	0	0	1
1	0	18	1	0	0	0	0	0	0	0	1	0	0	0	1	0	0	0	1	0	0	0	1
1	0	18	1	0	0	0	0	0	0	0	1	0	0	0	1	0	0	0	1	0	0	0	1
1	0	19	1	0	0	0	0	0	0	0	1	0	0	0	1	0	0	0	1	0	0	0	1
1	0	19	1	0	0	0	0	0	0	0	1	0	0	0	1	0	0	0	1	0	0	0	1
1	0	19	1	0	0	0	0	0	0	0	1	0	0	0	1	0	0	0	1	0	0	0	1
1	0	19	1	0	0	0	0	0	0	0	1	0	0	0	1	0	0	0	1	0	0	0	1
1	0	19	1	0	0	0	0	0	0	0	1	0	0	0	1	0	0	0	1	0	0	0	1
1	0	19	1	0	0	0	0	0	0	0	1	0	0	0	1	0	0	0	1	0	0	0	1
1	0	19	1	0	0	0	0	0	0	0	1	0	0	0	1	0	0	0	1	0	0	0	1
1	0	19	1	0	0	0	0	0	0	0	1	0	0	0	1	0	0	0	1	0	0	0	1
1	0	20	1	0	0	0	0	0	0	0	1	0	0	0	1	0	0	0	1	0	0	0	1
1	0	18	1	0	0	0	0	0	0	0	1	0	0	0	1	0	0	0	1	0	0	0	1
1	0	18	1	0	0	0	0	0	0	0	1	0	0	0	1	0	0	0	1	0	0	0	1
1	0	18	1	0	0	0	0	0	0	0	1	0	0	0	1	0	0	0	1	0	0	0	1
1	0	18	1	0	0	0	0	0	0	0	1	0	0	0	1	0	0	0	1	0	0	0	1
1	0	18	1	0	0	0	0	0	0	0	1	0	0	0	1	0	0	0	1	0	0	0	1
1	0	18	0	0	1	0	0	0	0	0	1	0	0	0	1	0	0	0	1	0	0	0	1
1	0	19	1	0	0	0	0	0	0	0	1	0	0	0	1	0	0	0	1	0	0	0	1
1	0	19	1	0	0	0	0	0	0	0	1	0	0	0	1	0	0	0	1	0	0	0	1
1	0	19	1	0	0	0	0	0	0	0	1	0	0	0	1	0	0	0	1	0	0	0	1
1	0	19	1	0	0	0	0	0	0	0	1	0	0	0	1	0	0	0	1	0	0	0	1
1	0	19	1	0	0	0	0	0	0	0	1	0	0	0	1	0	0	0	1	0	0	0	1
1	0	19	1	0	0	0	0	0	0	0	1	0	0	0	1	0	0	0	1	0	0	0	1
1	0	19	1	0	0	0	0	0	0	0	1	0	0	0	1	0	0	0	1	0	0	0	1
1	0	19	0	0	1	0	0	0	0	0	1	0	0	0	1	0	0	0	1	0	0	0	1
1	0	19	1	0	0	0	0	0	0	0	1	0	0	0	1	0	0	0	1	0	0	0	1

1	0	20	1	0	0	0	0	0	0	0	1	0	0	0	1	0	0	0	1	0	0	0	1
1	0	23	1	0	0	0	0	0	0	0	1	0	0	0	1	0	0	0	1	0	0	0	1
1	0	18	1	0	0	0	0	0	0	0	1	0	0	0	1	0	0	0	1	0	0	0	1
1	0	19	1	0	0	0	0	0	0	0	1	0	0	0	1	0	0	0	1	0	0	0	1
1	0	18	1	0	0	13	3	1	0	1	0	1	0	0	0	0	0	0	1	0	0	1	0
1	0	20	0	0	1	13	4	1	0	1	0	0	1	0	0	0	0	0	1	0	1	0	0
1	0	20	1	0	0	13	3	1	0	1	0	1	0	0	0	0	0	0	1	0	1	0	0
1	0	18	1	0	0	13	1	1	0	1	0	0	0	1	0	0	0	0	1	0	1	0	0
1	0	20	0	0	1	13	5	1	0	1	0	0	0	1	0	0	0	0	1	0	1	0	0
1	0	18	1	0	0	14	3	1	1	1	0	1	0	0	0	0	0	1	0	0	1	0	0
1	0	20	1	0	0	14	3	1	0	1	0	0	1	0	0	0	0	0	1	0	1	0	0
1	0	22	1	0	0	14	9	1	0	1	0	0	0	1	0	0	0	0	1	0	1	0	0
1	0	19	1	0	0	14	5	1	0	0	0	1	0	0	0	0	0	0	1	0	0	0	1
1	0	20	1	0	0	14	10	1	0	1	0	0	0	1	0	0	0	0	1	0	1	0	0
1	0	18	1	0	0	14	18	1	0	0	0	1	0	0	0	0	0	0	1	0	0	0	1
1	0	18	1	0	0	14	3	1	0	1	0	1	0	0	0	0	0	0	1	0	1	0	0
1	0	19	0	0	1	14	3	1	0	1	0	1	0	0	0	0	0	0	1	0	0	1	0
1	0	19	1	0	0	14	8	1	0	1	0	0	0	1	0	0	0	0	1	0	1	0	0
1	0	21	1	0	0	14	7	1	0	1	0	0	0	1	0	0	0	0	1	0	1	0	0
1	0	18	1	0	0	14	5	1	0	1	0	1	0	0	0	0	0	0	1	0	1	0	0
1	0	19	0	0	1	14	5	1	1	1	0	0	0	1	0	1	0	0	0	0	0	1	0
1	0	21	1	0	0	14	4	1	1	1	0	0	0	1	0	1	0	0	0	0	1	0	0
1	0	21	1	0	0	14	11	1	0	1	0	0	0	1	0	0	0	0	1	0	0	1	0
1	0	18	1	0	0	14	3	1	0	1	0	0	0	1	0	0	0	0	1	0	1	0	0
1	0	18	1	0	0	14	2	1	1	1	0	1	0	0	0	1	0	0	0	0	0	1	0
1	0	20	1	0	0	14	4	1	0	1	0	1	0	0	0	0	0	0	1	0	0	1	0
1	0	18	1	0	0	15	2	1	0	0	0	0	0	1	0	0	0	0	1	0	0	0	1
1	0	18	1	0	0	15	4	1	0	0	0	1	0	0	0	0	0	0	1	0	0	0	1
1	0	18	1	0	0	15	1	1	0	1	0	0	0	1	0	0	0	0	1	0	1	0	0
1	0	18	1	0	0	15	1	1	0	1	0	1	0	0	0	0	0	0	1	0	1	0	0
1	0	18	1	0	0	15	2	1	1	1	0	0	0	1	0	0	1	0	0	0	1	0	0
1	0	18	0	0	1	15	3	1	1	1	0	0	0	1	0	1	0	0	0	0	1	0	0
1	0	18	1	0	0	15	3	1	1	1	0	0	0	1	0	0	1	0	0	0	1	0	0
1	0	18	0	1	0	15	6	1	0	1	0	1	0	0	0	0	0	0	1	0	1	0	0
1	0	19	1	0	0	15	1	1	0	0	0	1	0	0	0	0	0	0	1	0	0	0	1
1	0	19	1	0	0	15	2	1	0	0	0	0	0	1	0	0	0	0	1	0	0	0	1
1	0	19	1	0	0	15	2	1	0	1	0	0	0	1	0	0	0	0	1	0	1	0	0
1	0	19	0	0	1	15	3	1	0	1	0	0	0	1	0	0	0	0	1	0	1	0	0
1	0	20	1	0	0	15	2	1	0	0	0	0	0	1	0	0	0	0	1	0	0	0	1
1	0	20	0	0	1	15	1	1	1	1	0	1	0	0	0	1	0	0	0	0	1	0	0
1	0	20	1	0	0	15	8	1	0	1	0	1	0	0	0	0	0	0	1	0	1	0	0
1	0	21	1	0	0	15	1	1	0	0	0	1	0	0	0	0	0	0	1	0	0	0	1
1	0	21	1	0	0	15	2	1	0	0	0	0	1	0	0	0	0	0	1	0	0	0	1
1	0	21	1	0	0	15	10	1	1	1	0	1	0	0	0	1	0	0	0	0	1	0	0
1	0	23	1	0	0	15	3	1	0	1	0	1	0	0	0	0	0	0	1	0	1	0	0
1	0	24	1	0	0	15	3	1	0	0	0	1	0	0	0	0	0	0	1	0	0	0	1
1	0	19	1	0	0	15	1	1	0	1	0	0	0	1	0	0	0	0	1	0	1	0	0
1	0	18	1	0	0	15	1	1	0	0	0	0	0	1	0	0	0	0	1	0	0	0	1
1	0	18	1	0	0	15	1	1	0	1	0	0	0	1	0	0	0	0	1	0	1	0	0
1	0	18	1	0	0	15	1	1	0	0	0	1	0	0	0	0	0	0	1	0	0	0	1

1	0	18	1	0	0	15	3	1	0	1	0	1	0	0	0	0	0	0	1	0	1	0	0
1	0	21	1	0	0	15	1	1	0	0	0	0	0	1	0	0	0	0	1	0	0	0	1
1	0	18	1	0	0	15	4	1	0	1	0	1	0	0	0	0	0	0	1	0	1	0	0
1	0	19	1	0	0	15	2	1	0	0	0	1	0	0	0	0	0	0	1	0	0	0	1
1	0	19	1	0	0	15	2	1	0	1	0	1	0	0	0	0	0	0	1	0	0	1	0
1	0	19	0	0	1	15	3	1	1	1	0	1	0	0	0	0	1	0	0	0	1	0	0
1	0	19	1	0	0	15	4	1	0	1	0	0	0	1	0	0	0	0	1	0	1	0	0
1	0	20	1	0	0	15	4	1	1	1	0	0	0	1	0	0	1	0	0	0	0	1	0
1	0	21	1	0	0	15	1	1	1	0	0	0	0	1	0	1	0	0	0	0	0	0	1
1	0	18	1	0	0	15	1	1	0	0	0	1	0	0	0	0	0	0	1	0	0	0	1
1	0	18	1	0	0	15	3	1	0	0	0	1	0	0	0	0	0	0	1	0	0	0	1
1	0	18	1	0	0	15	4	1	0	0	0	0	0	1	0	0	0	0	1	0	0	0	1
1	0	18	1	0	0	15	4	1	0	0	0	1	0	0	0	0	0	0	1	0	0	0	1
1	0	18	1	0	0	15	5	1	0	0	0	1	0	0	0	0	0	0	1	0	0	0	1
1	0	18	1	0	0	15	1	1	0	1	0	1	0	0	0	0	0	0	1	0	0	1	0
1	0	18	1	0	0	15	1	1	0	1	0	1	0	0	0	0	0	0	1	0	1	0	0
1	0	18	1	0	0	15	2	1	0	1	0	1	0	0	0	0	0	0	1	1	0	0	0
1	0	18	1	0	0	15	2	1	1	1	0	0	0	1	0	0	1	0	0	0	0	1	0
1	0	18	1	0	0	15	3	1	0	1	0	0	1	0	0	0	0	0	1	0	1	0	0
1	0	18	0	0	1	15	5	1	1	1	0	1	0	0	0	0	1	0	0	0	1	0	0
1	0	18	1	0	0	15	6	1	1	1	0	0	0	1	0	1	0	0	0	0	1	0	0
1	0	19	1	0	0	15	1	1	0	0	0	0	0	1	0	0	0	0	1	0	0	0	1
1	0	19	1	0	0	15	2	1	0	0	0	1	0	0	0	0	0	0	1	0	0	0	1
1	0	19	1	0	0	15	3	1	0	0	0	1	0	0	0	0	0	0	1	0	0	0	1
1	0	19	1	0	0	15	4	1	0	0	0	1	0	0	0	0	0	0	1	0	0	0	1
1	0	19	1	0	0	15	4	1	0	0	0	1	0	0	0	0	0	0	1	0	0	0	1
1	0	19	1	0	0	15	1	1	0	1	0	1	0	0	0	0	0	0	1	0	1	0	0
1	0	19	1	0	0	15	2	1	0	1	0	1	0	0	0	0	0	0	1	0	1	0	0
1	0	19	1	0	0	15	3	1	0	1	0	0	0	1	0	0	0	0	1	0	0	1	0
1	0	19	1	0	0	15	3	1	0	1	0	0	0	1	0	0	0	0	1	0	1	0	0
1	0	19	1	0	0	15	4	1	0	1	0	0	0	1	0	0	0	0	1	0	0	1	0
1	0	20	1	0	0	15	4	1	0	0	0	1	0	0	0	0	0	0	1	0	0	0	1
1	0	20	0	0	1	15	2	1	0	1	0	1	0	0	0	0	0	0	1	0	0	1	0
1	0	20	1	0	0	15	4	1	0	1	0	1	0	0	0	0	0	0	1	0	1	0	0
1	0	21	1	0	0	15	3	1	0	1	0	1	0	0	0	0	0	0	1	0	1	0	0
1	0	22	1	0	0	15	8	1	0	1	0	0	0	1	0	0	0	0	1	0	1	0	0
1	0	18	1	0	0	15	1	1	0	0	0	1	0	0	0	0	0	0	1	0	0	0	1
1	0	18	1	0	0	15	2	1	1	1	0	0	0	1	0	0	1	0	0	0	1	0	0
1	0	18	1	0	0	15	2	1	0	1	0	1	0	0	0	0	0	0	1	1	0	0	0
1	0	18	0	1	0	15	3	1	0	1	0	0	0	1	0	0	0	0	1	0	1	0	0
1	0	18	0	0	1	15	6	1	1	1	0	0	0	1	0	0	1	0	0	0	1	0	0
1	0	19	0	1	0	15	1	1	0	1	0	1	0	0	0	0	0	0	1	0	1	0	0
1	0	19	1	0	0	15	2	1	0	1	0	1	0	0	0	0	0	0	1	1	0	0	0
1	0	19	1	0	0	15	9	1	1	1	0	1	0	0	0	1	0	0	0	0	0	1	0
1	0	20	1	0	0	15	2	1	0	1	0	0	0	1	0	0	0	0	1	0	1	0	0
1	0	18	1	0	0	15	1	1	0	1	0	0	1	0	0	0	0	0	1	0	1	0	0
1	0	19	1	0	0	15	1	1	0	0	0	1	0	0	0	0	0	0	1	0	0	0	1
1	0	18	1	0	0	15	2	1	0	1	0	1	0	0	0	0	0	0	1	0	0	1	0
1	0	18	0	1	0	15	3	1	0	1	0	1	0	0	0	0	0	0	1	0	1	0	0
1	0	18	1	0	0	15	4	1	0	0	0	1	0	0	0	0	0	0	1	0	0	0	1

1	0	18	1	0	0	15	1	1	0	0	0	1	0	0	0	0	0	0	1	0	0	0	1
1	0	23	1	0	0	15	3	1	0	1	0	0	1	0	0	0	0	0	1	0	1	0	0
1	0	23	0	1	0	15	8	1	0	1	0	0	1	0	0	0	0	0	1	0	1	0	0
1	0	18	1	0	0	16	1	1	0	0	0	1	0	0	0	0	0	0	1	0	0	0	1
1	0	18	1	0	0	16	1	1	0	0	0	0	0	1	0	0	0	0	1	0	0	0	1
1	0	18	1	0	0	16	2	1	0	0	0	1	0	0	0	0	0	0	1	0	0	0	1
1	0	18	1	0	0	16	2	1	0	0	0	1	0	0	0	0	0	0	1	0	0	0	1
1	0	18	1	0	0	16	3	1	0	0	0	1	0	0	0	0	0	0	1	0	0	0	1
1	0	18	1	0	0	16	3	1	0	0	0	1	0	0	0	0	0	0	1	0	0	0	1
1	0	18	1	0	0	16	1	1	0	1	0	1	0	0	0	0	0	0	1	0	1	0	0
1	0	18	1	0	0	16	5	1	0	1	0	1	0	0	0	0	0	0	1	0	1	0	0
1	0	18	0	0	1	16	6	1	0	1	0	1	0	0	0	0	0	0	1	0	1	0	0
1	0	19	1	0	0	16	1	1	0	0	0	1	0	0	0	0	0	0	1	0	0	0	1
1	0	19	1	0	0	16	1	1	0	0	0	0	1	0	0	0	0	0	1	0	0	0	1
1	0	19	1	0	0	16	3	1	0	0	0	1	0	0	0	0	0	0	1	0	0	0	1
1	0	19	1	0	0	16	3	1	0	0	0	1	0	0	0	0	0	0	1	0	0	0	1
1	0	19	1	0	0	16	4	1	0	0	0	1	0	0	0	0	0	0	1	0	0	0	1
1	0	19	1	0	0	16	1	1	0	1	0	0	0	1	0	0	0	0	1	0	1	0	0
1	0	19	1	0	0	16	2	1	1	1	0	0	0	1	0	0	1	0	0	0	0	1	0
1	0	19	1	0	0	16	2	1	1	1	0	0	1	0	0	0	1	0	0	0	1	0	0
1	0	19	1	0	0	16	2	1	0	1	0	1	0	0	0	0	0	0	1	0	0	1	0
1	0	19	1	0	0	16	3	1	0	1	0	1	0	0	0	0	0	0	1	0	1	0	0
1	0	19	1	0	0	16	3	1	0	1	0	1	0	0	0	0	0	0	1	1	0	0	0
1	0	19	1	0	0	16	4	1	0	1	0	1	0	0	0	0	0	0	1	0	0	1	0
1	0	19	1	0	0	16	5	1	0	1	0	0	0	1	0	0	0	0	1	0	1	0	0
1	0	19	1	0	0	16	6	1	0	1	0	0	0	1	0	0	0	0	1	0	1	0	0
1	0	20	1	0	0	16	2	1	0	0	0	1	0	0	0	0	0	0	1	0	0	0	1
1	0	20	1	0	0	16	1	1	1	1	0	0	0	1	0	0	1	0	0	0	1	0	0
1	0	21	1	0	0	16	3	1	0	0	0	1	0	0	0	0	0	0	1	0	0	0	1
1	0	21	1	0	0	16	3	1	0	0	0	1	0	0	0	0	0	0	1	0	0	0	1
1	0	21	1	0	0	16	4	1	0	0	0	0	0	1	0	0	0	0	1	0	0	0	1
1	0	18	1	0	0	16	3	1	0	1	0	1	0	0	0	0	0	0	1	0	0	1	0
1	0	18	1	0	0	16	3	1	0	1	0	1	0	0	0	0	0	0	1	0	1	0	0
1	0	18	1	0	0	16	3	1	1	1	0	1	0	0	0	1	0	0	0	0	0	1	0
1	0	18	1	0	0	16	5	1	0	1	0	1	0	0	0	0	0	0	1	0	1	0	0
1	0	19	1	0	0	16	2	1	0	0	0	1	0	0	0	0	0	0	1	0	0	0	1
1	0	19	1	0	0	16	2	1	0	1	0	1	0	0	0	0	0	0	1	1	0	0	0
1	0	20	1	0	0	16	1	1	0	0	0	0	0	1	0	0	0	0	1	0	0	0	1
1	0	20	1	0	0	16	6	1	0	1	0	1	0	0	0	0	0	0	1	0	1	0	0
1	0	20	1	0	0	16	10	1	1	1	0	0	0	1	0	1	0	0	0	0	0	1	0
1	0	18	1	0	0	16	1	1	0	0	0	1	0	0	0	0	0	0	1	0	0	0	1
1	0	18	1	0	0	16	2	1	0	0	0	1	0	0	0	0	0	0	1	0	0	0	1
1	0	18	1	0	0	16	4	1	0	0	0	1	0	0	0	0	0	0	1	0	0	0	1
1	0	18	1	0	0	16	1	1	0	1	0	1	0	0	0	0	0	0	1	0	1	0	0
1	0	18	0	0	1	16	1	1	0	1	0	1	0	0	0	0	0	0	1	1	0	0	0
1	0	19	1	0	0	16	2	1	0	0	0	1	0	0	0	0	0	0	1	0	0	0	1
1	0	19	1	0	0	16	3	1	0	1	0	1	0	0	0	0	0	0	1	0	0	1	0
1	0	20	1	0	0	16	3	1	0	0	0	0	0	1	0	0	0	0	1	0	0	0	1
1	0	20	1	0	0	16	4	1	0	1	0	1	0	0	0	0	0	0	1	0	1	0	0
1	0	22	1	0	0	16	3	1	0	0	0	1	0	0	0	0	0	0	1	0	0	0	1

1	0	22	1	0	0	16	4	1	1	1	0	0	0	1	0	1	0	0	0	0	0	1	0
1	0	18	1	0	0	16	1	1	0	0	0	1	0	0	0	0	0	0	1	0	0	0	1
1	0	18	1	0	0	16	1	1	0	0	0	1	0	0	0	0	0	0	1	0	0	0	1
1	0	18	1	0	0	16	2	1	0	0	0	1	0	0	0	0	0	0	1	0	0	0	1
1	0	18	1	0	0	16	5	1	0	0	0	1	0	0	0	0	0	0	1	0	0	0	1
1	0	18	1	0	0	16	1	1	0	1	0	1	0	0	0	0	0	0	1	0	1	0	0
1	0	18	1	0	0	16	1	1	1	1	0	1	0	0	0	1	0	0	0	0	1	0	0
1	0	18	1	0	0	16	2	1	0	1	0	1	0	0	0	0	0	0	1	0	1	0	0
1	0	18	1	0	0	16	2	1	0	1	0	1	0	0	0	0	0	0	1	0	0	1	0
1	0	18	1	0	0	16	4	1	1	1	0	1	0	0	0	1	0	0	0	1	0	0	0
1	0	19	1	0	0	16	1	1	0	0	0	1	0	0	0	0	0	0	1	0	0	0	1
1	0	19	1	0	0	16	2	1	0	0	0	0	0	1	0	0	0	0	1	0	0	0	1
1	0	19	1	0	0	16	2	1	0	1	0	1	0	0	0	0	0	0	1	0	0	1	0
1	0	19	1	0	0	16	2	1	0	1	0	0	1	0	0	0	0	0	1	0	1	0	0
1	0	19	1	0	0	16	3	1	0	1	0	1	0	0	0	0	0	0	1	0	1	0	0
1	0	19	1	0	0	16	4	1	0	1	0	0	0	1	0	0	0	0	1	0	0	1	0
1	0	19	0	0	1	16	4	1	0	1	0	1	0	0	0	0	0	0	1	1	0	0	0
1	0	19	1	0	0	16	11	1	0	1	0	0	0	1	0	0	0	0	1	0	1	0	0
1	0	20	1	0	0	16	2	1	0	1	0	1	0	0	0	0	0	0	1	0	0	1	0
1	0	20	1	0	0	16	2	1	0	1	0	1	0	0	0	0	0	0	1	0	1	0	0
1	0	20	1	0	0	16	2	1	1	1	0	0	0	1	0	0	1	0	0	0	1	0	0
1	0	21	1	0	0	16	3	1	0	1	0	1	0	0	0	0	0	0	1	0	1	0	0
1	0	18	1	0	0	16	1	1	0	0	0	1	0	0	0	0	0	0	1	0	0	0	1
1	0	18	0	0	1	16	1	1	0	0	0	0	0	1	0	0	0	0	1	0	0	0	1
1	0	18	1	0	0	16	4	1	0	0	0	0	0	1	0	0	0	0	1	0	0	0	1
1	0	18	1	0	0	16	1	1	0	1	0	1	0	0	0	0	0	0	1	0	1	0	0
1	0	18	1	0	0	16	1	1	0	1	0	0	1	0	0	0	0	0	1	0	1	0	0
1	0	18	1	0	0	16	1	1	0	1	0	0	0	1	0	0	0	0	1	0	1	0	0
1	0	18	1	0	0	16	1	1	1	1	0	1	0	0	0	1	0	0	0	0	0	1	0
1	0	18	1	0	0	16	2	1	0	1	0	1	0	0	0	0	0	0	1	0	1	0	0
1	0	18	1	0	0	16	2	1	0	1	0	1	0	0	0	0	0	0	1	1	0	0	0
1	0	18	1	0	0	16	2	1	0	1	0	1	0	0	0	0	0	0	1	0	1	0	0
1	0	18	0	0	1	16	2	1	0	1	0	1	0	0	0	0	0	0	1	1	0	0	0
1	0	18	1	0	0	16	7	1	0	1	0	1	0	0	0	0	0	0	1	0	1	0	0
1	0	19	1	0	0	16	1	1	0	0	0	0	0	1	0	0	0	0	1	0	0	0	1
1	0	19	1	0	0	16	1	1	0	0	0	1	0	0	0	0	0	0	1	0	0	0	1
1	0	19	1	0	0	16	2	1	0	0	0	1	0	0	0	0	0	0	1	0	0	0	1
1	0	19	1	0	0	16	2	1	0	0	0	1	0	0	0	0	0	0	1	0	0	0	1
1	0	19	1	0	0	16	1	1	0	1	0	0	1	0	0	0	0	0	1	0	1	0	0
1	0	19	1	0	0	16	1	1	0	1	0	1	0	0	0	0	0	0	1	0	0	1	0
1	0	19	1	0	0	16	2	1	0	1	0	0	0	1	0	0	0	0	1	0	1	0	0
1	0	19	1	0	0	16	2	1	0	1	0	1	0	0	0	0	0	0	1	1	0	0	0
1	0	19	1	0	0	16	4	1	1	1	0	0	0	1	0	0	0	1	0	0	1	0	0
1	0	19	0	0	1	16	7	1	0	1	0	1	0	0	0	0	0	0	1	0	0	0	0
1	0	20	1	0	0	16	1	1	0	0	0	0	0	1	0	0	0	0	1	0	0	0	1
1	0	20	1	0	0	16	1	1	0	0	0	1	0	0	0	0	0	0	1	0	0	0	1
1	0	20	1	0	0	16	3	1	0	0	0	0	0	1	0	0	0	0	1	0	0	0	1
1	0	20	1	0	0	16	7	1	0	0	0	1	0	0	0	0	0	0	1	0	0	0	1
1	0	20	1	0	0	16	1	1	0	1	0	1	0	0	0	0	0	0	1	0	1	0	0
1	0	20	1	0	0	16	1	1	0	1	0	1	0	0	0	0	0	0	1	0	1	0	0

1	0	20	1	0	0	16	3	1	0	1	0	0	0	1	0	0	0	0	1	0	1	0	0
1	0	20	0	0	1	16	10	1	0	1	0	1	0	0	0	0	0	0	1	0	0	1	0
1	0	21	1	0	0	16	4	1	0	0	0	0	0	1	0	0	0	0	1	0	0	0	1
1	0	18	1	0	0	16	1	1	0	0	0	1	0	0	0	0	0	0	1	0	0	0	1
1	0	18	1	0	0	16	1	1	0	0	0	0	0	1	0	0	0	0	1	0	0	0	1
1	0	18	1	0	0	16	2	1	0	0	0	1	0	0	0	0	0	0	1	0	0	0	1
1	0	18	1	0	0	16	2	1	1	0	0	0	0	1	0	0	0	1	0	0	0	0	1
1	0	18	0	0	1	16	2	1	0	0	0	1	0	0	0	0	0	0	1	0	0	0	1
1	0	18	1	0	0	16	2	1	0	0	0	1	0	0	0	0	0	0	1	0	0	0	1
1	0	18	1	0	0	16	3	1	1	1	0	0	0	1	0	0	0	0	0	0	0	1	0
1	0	19	1	0	0	16	1	1	0	0	0	1	0	0	0	0	0	0	1	0	0	0	1
1	0	19	1	0	0	16	3	1	0	0	0	1	0	0	0	0	0	0	1	0	0	0	1
1	0	21	1	0	0	16	6	1	0	1	0	1	0	0	0	0	0	0	1	1	0	0	0
1	0	18	1	0	0	16	1	1	0	1	0	1	0	0	0	0	0	0	1	0	1	0	0
1	0	18	1	0	0	16	2	1	0	1	0	1	0	0	0	0	0	0	1	0	1	0	0
1	0	19	0	0	1	16	1	1	0	0	0	1	0	0	0	0	0	0	1	0	0	0	1
1	0	19	1	0	0	16	1	1	0	0	0	1	0	0	0	0	0	0	1	0	0	0	1
1	0	20	1	0	0	16	1	1	0	1	0	0	0	1	0	0	0	0	1	0	0	1	0
1	0	18	1	0	0	16	1	1	0	0	0	1	0	0	0	0	0	0	1	0	0	0	1
1	0	18	1	0	0	16	2	1	0	0	0	1	0	0	0	0	0	0	1	0	0	0	1
1	0	18	1	0	0	16	2	1	1	1	0	1	0	0	0	0	0	1	0	0	1	0	0
1	0	18	1	0	0	16	4	1	0	1	0	1	0	0	0	0	0	0	1	0	1	0	0
1	0	19	1	0	0	16	3	1	0	0	0	1	0	0	0	0	0	0	1	0	0	0	1
1	0	20	1	0	0	16	1	1	0	0	0	0	0	1	0	0	0	0	1	0	0	0	1
1	0	19	1	0	0	16	2	1	1	1	0	0	0	1	0	0	0	1	0	0	0	1	0
1	0	18	1	0	0	16	2	1	0	1	0	0	0	1	0	0	0	0	1	0	0	1	0
1	0	20	1	0	0	16	2	1	0	0	0	0	0	1	0	0	0	0	1	0	0	0	1
1	0	19	1	0	0	16	2	1	0	0	0	1	0	0	0	0	0	0	1	0	0	0	1
1	0	19	1	0	0	16	3	1	0	0	0	0	0	1	0	0	0	0	1	0	0	0	1
1	0	21	1	0	0	16	5	1	0	0	0	0	0	1	0	0	0	0	1	0	0	0	1
1	0	18	1	0	0	17	1	1	0	0	0	0	0	1	0	0	0	0	1	0	0	0	1
1	0	18	1	0	0	17	1	1	0	0	0	1	0	0	0	0	0	0	1	0	0	0	1
1	0	18	1	0	0	17	1	1	0	0	0	1	0	0	0	0	0	0	1	0	0	0	1
1	0	18	1	0	0	17	1	1	0	0	0	1	0	0	0	0	0	0	1	0	0	0	1
1	0	18	0	0	1	17	1	1	0	0	0	1	0	0	0	0	0	0	1	0	0	0	1
1	0	18	1	0	0	17	1	1	0	0	0	1	0	0	0	0	0	0	1	0	0	0	1
1	0	18	1	0	0	17	1	1	0	0	0	1	0	0	0	0	0	0	1	0	0	0	1
1	0	18	1	0	0	17	2	1	0	0	0	1	0	0	0	0	0	0	1	0	0	0	1
1	0	18	1	0	0	17	2	1	0	0	0	1	0	0	0	0	0	0	1	0	0	0	1
1	0	18	1	0	0	17	1	1	0	1	0	1	0	0	0	0	0	0	1	0	1	0	0
1	0	18	1	0	0	17	1	1	0	1	0	1	0	0	0	0	0	0	1	0	1	0	0
1	0	18	1	0	0	17	1	1	0	1	0	1	0	0	0	0	0	0	1	0	1	0	0
1	0	18	1	0	0	17	1	1	0	1	0	0	1	0	0	0	0	0	1	0	1	0	0
1	0	18	1	0	0	17	2	1	0	1	0	0	0	1	0	0	0	0	1	0	1	0	0
1	0	18	1	0	0	17	2	1	0	1	0	1	0	0	0	0	0	0	1	0	0	1	0
1	0	18	1	0	0	17	2	1	0	1	0	0	0	1	0	0	0	0	1	0	1	0	0
1	0	18	1	0	0	17	3	1	1	1	0	1	0	0	0	1	0	0	0	0	0	1	0
1	0	19	1	0	0	17	1	1	0	0	0	0	0	1	0	0	0	0	1	0	0	0	1
1	0	19	1	0	0	17	1	1	0	0	0	1	0	0	0	0	0	0	1	0	0	0	1
1	0	19	1	0	0	17	1	1	0	0	0	1	0	0	0	0	0	0	1	0	0	0	1

1	0	19	1	0	0	17	1	1	0	0	0	1	0	0	0	0	0	0	1	0	0	0	1
1	0	19	1	0	0	17	1	1	0	0	0	1	0	0	0	0	0	0	1	0	0	0	1
1	0	19	1	0	0	17	1	1	0	0	0	1	0	0	0	0	0	0	1	0	0	0	1
1	0	19	1	0	0	17	1	1	0	0	0	0	1	0	0	0	0	0	1	0	0	0	1
1	0	19	1	0	0	17	1	1	0	0	0	1	0	0	0	0	0	0	1	0	0	0	1
1	0	19	1	0	0	17	2	1	0	0	0	1	0	0	0	0	0	0	1	0	0	0	1
1	0	19	1	0	0	17	2	1	0	0	0	1	0	0	0	0	0	0	1	0	0	0	1
1	0	19	1	0	0	17	2	1	0	0	0	1	0	0	0	0	0	0	1	0	0	0	1
1	0	19	1	0	0	17	3	1	0	0	0	1	0	0	0	0	0	0	1	0	0	0	1
1	0	19	1	0	0	17	5	1	0	0	0	1	0	0	0	0	0	0	1	0	0	0	1
1	0	19	1	0	0	17	1	1	0	1	0	0	0	1	0	0	0	0	1	1	0	0	0
1	0	19	1	0	0	17	1	1	0	1	0	1	0	0	0	0	0	0	1	0	1	0	0
1	0	19	1	0	0	17	2	1	0	1	0	0	0	1	0	0	0	0	1	0	1	0	0
1	0	19	1	0	0	17	2	1	0	1	0	1	0	0	0	0	0	0	1	0	1	0	0
1	0	19	1	0	0	17	3	1	0	1	0	1	0	0	0	0	0	0	1	1	0	0	0
1	0	19	1	0	0	17	6	1	0	1	0	1	0	0	0	0	0	0	1	0	1	0	0
1	0	20	1	0	0	17	1	1	0	0	0	1	0	0	0	0	0	0	1	0	0	0	1
1	0	20	1	0	0	17	1	1	0	0	0	1	0	0	0	0	0	0	1	0	0	0	1
1	0	20	1	0	0	17	2	1	0	0	0	0	0	1	0	0	0	0	1	0	0	0	1
1	0	20	0	0	1	17	5	1	0	0	0	0	1	0	0	0	0	0	1	0	0	0	1
1	0	20	1	0	0	17	1	1	0	1	0	0	0	1	0	0	0	0	1	0	1	0	0
1	0	20	1	0	0	17	1	1	0	1	0	1	0	0	0	0	0	0	1	0	1	0	0
1	0	20	1	0	0	17	1	1	0	1	0	0	0	1	0	0	0	0	1	0	1	0	0
1	0	20	1	0	0	17	2	1	0	1	0	0	1	0	0	0	0	0	1	0	1	0	0
1	0	20	1	0	0	17	3	1	0	1	0	0	0	1	0	0	0	0	1	0	1	0	0
1	0	21	1	0	0	17	1	1	1	1	0	0	1	0	0	0	0	1	0	0	1	0	0
1	0	22	1	0	0	17	3	1	1	1	0	0	0	1	0	1	0	0	0	0	1	0	0
1	0	24	1	0	0	17	15	1	0	1	0	0	0	1	0	0	0	0	1	0	1	0	0
1	0	18	1	0	0	17	1	1	0	0	0	1	0	0	0	0	0	0	1	0	0	0	1
1	0	18	1	0	0	17	1	1	0	0	0	1	0	0	0	0	0	0	1	0	0	0	1
1	0	18	1	0	0	17	1	1	0	1	0	1	0	0	0	0	0	0	1	0	1	0	0
1	0	18	1	0	0	17	1	1	1	1	0	1	0	0	0	1	0	0	0	0	1	0	0
1	0	18	1	0	0	17	1	1	1	1	0	1	0	0	0	0	1	0	0	0	1	0	0
1	0	18	1	0	0	17	1	1	0	1	0	1	0	0	0	0	0	0	1	0	1	0	0
1	0	19	1	0	0	17	1	1	0	0	0	1	0	0	0	0	0	0	1	0	0	0	1
1	0	19	1	0	0	17	1	1	0	0	0	1	0	0	0	0	0	0	1	0	0	0	1
1	0	19	1	0	0	17	1	1	0	0	0	1	0	0	0	0	0	0	1	0	0	0	1
1	0	19	1	0	0	17	2	1	0	0	0	1	0	0	0	0	0	0	1	0	0	0	1
1	0	19	1	0	0	17	2	1	0	1	0	0	1	0	0	0	0	0	1	0	1	0	0
1	0	20	1	0	0	17	1	1	0	1	0	1	0	0	0	0	0	0	1	0	1	0	0
1	0	20	1	0	0	17	1	1	0	1	0	1	0	0	0	0	0	0	1	0	1	0	0
1	0	22	1	0	0	17	4	1	0	1	0	0	0	1	0	0	0	0	1	0	1	0	0
1	0	18	1	0	0	17	1	1	0	1	0	0	0	1	0	0	0	0	1	0	1	0	0
1	0	18	1	0	0	17	2	1	0	1	0	1	0	0	0	0	0	0	1	0	0	1	0
1	0	19	1	0	0	17	1	1	0	1	0	1	0	0	0	0	0	0	1	0	1	0	0
1	0	19	1	0	0	17	2	1	0	1	0	0	0	1	0	0	0	0	1	0	0	1	0
1	0	20	1	0	0	17	1	1	0	1	0	0	1	0	0	0	0	0	1	0	1	0	0
1	0	21	1	0	0	17	3	1	0	0	0	1	0	0	0	0	0	0	1	0	0	0	1
1	0	22	1	0	0	17	4	1	0	0	0	1	0	0	0	0	0	0	1	0	0	0	1
1	0	18	1	0	0	17	2	1	0	0	0	1	0	0	0	0	0	0	1	0	0	0	1

1	0	18	1	0	0	17	3	1	0	0	0	1	0	0	0	0	0	0	1	0	0	0	1
1	0	18	1	0	0	17	4	1	0	0	0	1	0	0	0	0	0	0	1	0	0	0	1
1	0	18	1	0	0	17	7	1	0	0	0	1	0	0	0	0	0	0	1	0	0	0	1
1	0	18	1	0	0	17	1	1	0	1	0	1	0	0	0	0	0	0	1	0	1	0	0
1	0	18	1	0	0	17	1	1	0	1	0	0	0	1	0	0	0	0	1	0	1	0	0
1	0	18	1	0	0	17	2	1	0	1	0	1	0	0	0	0	0	0	1	0	1	0	0
1	0	18	1	0	0	17	2	1	0	1	0	1	0	0	0	0	0	0	1	1	0	0	0
1	0	18	1	0	0	17	2	1	0	1	0	1	0	0	0	0	0	0	1	1	0	0	0
1	0	18	1	0	0	17	3	1	0	1	0	0	0	1	0	0	0	0	1	0	1	0	0
1	0	19	1	0	0	17	1	1	0	0	0	1	0	0	0	0	0	0	1	0	0	0	1
1	0	19	1	0	0	17	2	1	0	0	0	0	0	1	0	0	0	0	1	0	0	0	1
1	0	19	1	0	0	17	2	1	0	0	0	1	0	0	0	0	0	0	1	0	0	0	1
1	0	19	0	0	1	17	2	1	0	0	0	1	0	0	0	0	0	0	1	0	0	0	1
1	0	19	1	0	0	17	2	1	0	0	0	1	0	0	0	0	0	0	1	0	0	0	1
1	0	19	1	0	0	17	1	1	0	1	0	1	0	0	0	0	0	0	1	0	0	1	0
1	0	19	1	0	0	17	1	1	0	1	0	1	0	0	0	0	0	0	1	0	1	0	0
1	0	19	1	0	0	17	2	1	1	1	0	1	0	0	0	1	0	0	0	0	1	0	0
1	0	19	1	0	0	17	2	1	0	1	0	1	0	0	0	0	0	0	1	0	1	0	0
1	0	19	1	0	0	17	3	1	0	1	0	0	0	1	0	0	0	0	1	0	0	1	0
1	0	20	1	0	0	17	1	1	1	1	0	0	0	1	0	0	1	0	0	0	1	0	0
1	0	20	1	0	0	17	3	1	0	1	0	1	0	0	0	0	0	0	1	0	1	0	0
1	0	18	1	0	0	17	1	1	0	0	0	1	0	0	0	0	0	0	1	0	0	0	1
1	0	18	1	0	0	17	1	1	0	0	0	1	0	0	0	0	0	0	1	0	0	0	1
1	0	18	1	0	0	17	1	1	0	0	0	1	0	0	0	0	0	0	1	0	0	0	1
1	0	18	1	0	0	17	1	1	0	0	0	1	0	0	0	0	0	0	1	0	0	0	1
1	0	18	1	0	0	17	1	1	0	0	0	1	0	0	0	0	0	0	1	0	0	0	1
1	0	18	1	0	0	17	1	1	0	0	0	1	0	0	0	0	0	0	1	0	0	0	1
1	0	18	1	0	0	17	1	1	0	0	0	1	0	0	0	0	0	0	1	0	0	0	1
1	0	18	1	0	0	17	1	1	0	0	0	1	0	0	0	0	0	0	1	0	0	0	1
1	0	18	1	0	0	17	2	1	0	0	0	1	0	0	0	0	0	0	1	0	0	0	1
1	0	18	1	0	0	17	2	1	0	0	0	1	0	0	0	0	0	0	1	0	0	0	1
1	0	18	1	0	0	17	2	1	0	0	0	0	0	1	0	0	0	0	1	0	0	0	1
1	0	18	0	0	1	17	2	1	0	0	0	1	0	0	0	0	0	0	1	0	0	0	1
1	0	18	1	0	0	17	2	1	0	0	0	1	0	0	0	0	0	0	1	0	0	0	1
1	0	18	1	0	0	17	1	1	0	1	0	0	0	1	0	0	0	0	1	0	0	1	0
1	0	18	1	0	0	17	1	1	0	1	0	1	0	0	0	0	0	0	1	0	1	0	0
1	0	18	1	0	0	17	1	1	0	1	0	1	0	0	0	0	0	0	1	1	0	0	0
1	0	18	1	0	0	17	1	1	0	1	0	0	0	1	0	0	0	0	1	1	0	0	0
1	0	18	1	0	0	17	1	1	0	1	0	0	0	1	0	0	0	0	1	0	0	1	0
1	0	18	1	0	0	17	1	1	0	1	0	1	0	0	0	0	0	0	1	0	1	0	0
1	0	18	1	0	0	17	1	1	1	1	0	0	0	1	0	0	1	0	0	0	1	0	0
1	0	18	1	0	0	17	1	1	0	1	0	0	0	1	0	0	0	0	1	0	1	0	0
1	0	18	1	0	0	17	1	1	0	1	0	0	0	1	0	0	0	0	1	0	1	0	0
1	0	18	1	0	0	17	2	1	1	1	0	1	0	0	0	0	0	1	0	0	1	0	0
1	0	18	1	0	0	17	2	1	0	1	0	1	0	0	0	0	0	0	1	1	0	0	0
1	0	18	1	0	0	17	2	1	0	1	0	1	0	0	0	0	0	0	1	0	1	0	0
1	0	19	1	0	0	17	1	1	0	0	0	1	0	0	0	0	0	0	1	0	0	0	1
1	0	19	1	0	0	17	1	1	0	0	0	0	0	1	0	0	0	0	1	0	0	0	1
1	0	19	1	0	0	17	1	1	0	0	0	1	0	0	0	0	0	0	1	0	0	0	1
1	0	19	1	0	0	17	1	1	0	0	0	1	0	0	0	0	0	0	1	0	0	0	1

1	0	19	1	0	0	17	1	1	0	0	0	0	0	1	0	0	0	0	1	0	0	0	1
1	0	19	1	0	0	17	2	1	0	0	0	1	0	0	0	0	0	0	1	0	0	0	1
1	0	19	1	0	0	17	2	1	0	0	0	1	0	0	0	0	0	0	1	0	0	0	1
1	0	19	1	0	0	17	3	1	0	0	0	1	0	0	0	0	0	0	1	0	0	0	1
1	0	19	1	0	0	17	3	1	0	0	0	1	0	0	0	0	0	0	1	0	0	0	1
1	0	19	1	0	0	17	1	1	1	1	0	0	1	0	0	0	1	0	0	0	1	0	0
1	0	19	1	0	0	17	1	1	0	1	0	0	0	1	0	0	0	0	1	0	1	0	0
1	0	19	1	0	0	17	2	1	0	1	0	0	0	1	0	0	0	0	1	0	1	0	0
1	0	19	1	0	0	17	3	1	0	1	0	1	0	0	0	0	0	0	1	0	1	0	0
1	0	19	1	0	0	17	3	1	0	1	0	0	0	1	0	0	0	0	1	0	1	0	0
1	0	19	1	0	0	17	4	1	0	1	0	1	0	0	0	0	0	0	1	0	1	0	0
1	0	19	0	0	1	17	8	1	0	1	0	1	0	0	0	0	0	0	1	0	1	0	0
1	0	20	1	0	0	17	1	1	0	0	0	1	0	0	0	0	0	0	1	0	0	0	1
1	0	20	1	0	0	17	4	1	0	0	0	1	0	0	0	0	0	0	1	0	0	0	1
1	0	20	1	0	0	17	1	1	0	1	0	1	0	0	0	0	0	0	1	0	0	1	0
1	0	20	1	0	0	17	2	1	0	1	0	0	1	0	0	0	0	0	1	0	1	0	0
1	0	20	1	0	0	17	3	1	1	1	0	1	0	0	0	1	0	0	0	0	1	0	0
1	0	20	1	0	0	17	3	1	0	1	0	0	0	1	0	0	0	0	1	0	0	1	0
1	0	20	0	0	1	17	4	1	0	1	0	0	0	1	0	0	0	0	1	0	1	0	0
1	0	20	1	0	0	17		1	0	1	0	1	0	0	0	0	0	0	1	0	0	1	0
1	0	21	1	0	0	17	2	1	0	0	0	0	1	0	0	0	0	0	1	0	0	0	1
1	0	21	0	0	1	17	10	1	0	1	0	1	0	0	0	0	0	0	1	0	0	1	0
1	0	26	0	1	0	17	4	1	0	1	0	0	0	1	0	0	0	0	1	0	0	1	0
1	0	18	1	0	0	17	1	1	0	0	0	1	0	0	0	0	0	0	1	0	0	0	1
1	0	18	1	0	0	17	1	1	0	0	0	1	0	0	0	0	0	0	1	0	0	0	1
1	0	19	0	0	1	17	4	1	0	1	0	1	0	0	0	0	0	0	1	0	0	1	0
1	0	19	1	0	0	17	4	1	0	1	0	1	0	0	0	0	0	0	1	0	1	0	0
1	0	18	1	0	0	17	1	1	0	0	0	1	0	0	0	0	0	0	1	0	0	0	1
1	0	19	1	0	0	17	2	1	0	0	0	1	0	0	0	0	0	0	1	0	0	0	1
1	0	19	1	0	0	17	3	1	0	1	0	0	0	1	0	0	0	0	1	0	0	1	0
1	0	20	1	0	0	17	2	1	0	0	0	1	0	0	0	0	0	0	1	0	0	0	1
1	0	20	0	0	1	17	3	1	0	0	0	0	0	1	0	0	0	0	1	0	0	0	1
1	0	20	1	0	0	17	2	1	1	1	0	0	1	0	0	0	1	0	0	0	1	0	0
1	0	21	0	0	1	17	5	1	0	0	0	0	0	1	0	0	0	0	1	0	0	0	1
1	0	18	1	0	0	17	1	1	0	0	0	1	0	0	0	0	0	0	1	0	0	0	1
1	0	19	1	0	0	17	2	1	0	1	0	0	0	1	0	0	0	0	1	0	1	0	0
1	0	20	1	0	0	17	1	1	0	1	0	1	0	0	0	0	0	0	1	0	1	0	0
1	0	20	0	0	1	17	3	1	0	1	0	1	0	0	0	0	0	0	1	1	0	0	0
1	0	20	1	0	0	17	4	1	0	1	0	0	0	1	0	0	0	0	1	0	1	0	0
1	0	18	1	0	0	17	2	1	0	0	0	1	0	0	0	0	0	0	1	0	0	0	1
1	0	18	1	0	0	17	6	1	0	0	0	1	0	0	0	0	0	0	1	0	0	0	1
1	0	19	1	0	0	17	2	1	0	0	0	1	0	0	0	0	0	0	1	0	0	0	1
1	0	19	1	0	0	17	1	1	0	1	0	0	0	1	0	0	0	0	1	0	1	0	0
1	0	19	1	0	0	17	2	1	0	1	0	1	0	0	0	0	0	0	1	0	0	1	0
1	0	21	1	0	0	17	4	1	0	0	0	0	1	0	0	0	0	0	1	0	0	0	1
1	0	18	1	0	0	17	2	1	0	0	0	1	0	0	0	0	0	0	1	0	0	0	1
1	0	19	1	0	0	17	1	1	0	0	0	1	0	0	0	0	0	0	1	0	0	0	1
1	0	19	1	0	0	17	1	1	0	1	0	1	0	0	0	0	0	0	1	0	1	0	0
1	0	20	1	0	0	17	2	1	0	0	0	1	0	0	0	0	0	0	1	0	0	0	1
1	0	22	1	0	0	17	8	1	0	0	0	1	0	0	0	0	0	0	1	0	0	0	1

1	0	19	1	0	0	17	1	1	0	0	0	1	0	0	0	0	0	0	1	0	0	0	1
1	0	19	1	0	0	17	2	1	0	1	0	1	0	0	0	0	0	0	1	1	0	0	0
1	0	20	1	0	0	17	3	1	0	0	0	1	0	0	0	0	0	0	1	0	0	0	1
1	0	19	1	0	0	17	2	1	0	1	0	1	0	0	0	0	0	0	1	0	1	0	0
1	0	18	1	0	0	18	1	1	0	0	0	1	0	0	0	0	0	0	1	0	0	0	1
1	0	18	1	0	0	18	1	1	0	0	0	1	0	0	0	0	0	0	1	0	0	0	1
1	0	18	1	0	0	18	1	1	0	0	0	1	0	0	0	0	0	0	1	0	0	0	1
1	0	18	1	0	0	18	1	1	0	0	0	1	0	0	0	0	0	0	1	0	0	0	1
1	0	18	1	0	0	18	1	1	0	0	0	0	1	0	0	0	0	0	1	0	0	0	1
1	0	18	1	0	0	18	1	1	0	0	0	1	0	0	0	0	0	0	1	0	0	0	1
1	0	18	1	0	0	18	1	1	0	1	0	0	0	1	0	0	0	0	1	0	1	0	0
1	0	18	1	0	0	18	1	1	0	1	0	1	0	0	0	0	0	0	1	1	0	0	0
1	0	18	1	0	0	18	1	1	0	1	0	0	0	1	0	0	0	0	1	0	1	0	0
1	0	18	1	0	0	18	1	1	0	1	0	0	0	1	0	0	0	0	1	0	1	0	0
1	0	18	0	0	1	18	2	1	0	1	0	0	0	1	0	0	0	0	1	0	1	0	0
1	0	19	1	0	0	18	1	1	0	0	0	1	0	0	0	0	0	0	1	0	0	0	1
1	0	19	1	0	0	18	1	1	0	0	0	1	0	0	0	0	0	0	1	0	0	0	1
1	0	19	1	0	0	18	1	1	0	0	0	1	0	0	0	0	0	0	1	1	0	0	1
1	0	19	1	0	0	18	1	1	0	0	0	1	0	0	0	0	0	0	1	0	0	0	1
1	0	19	1	0	0	18	1	1	0	0	0	0	1	0	0	0	0	0	1	0	0	0	1
1	0	19	1	0	0	18	2	1	0	0	0	1	0	0	0	0	0	0	1	0	0	0	1
1	0	19	1	0	0	18	2	1	0	0	0	1	0	0	0	0	0	0	1	0	0	0	1
1	0	19	1	0	0	18	1	1	0	1	0	1	0	0	0	0	0	0	1	0	1	0	0
1	0	19	1	0	0	18	1	1	0	1	0	1	0	0	0	0	0	0	1	0	1	0	0
1	0	19	1	0	0	18	1	1	0	1	0	1	0	0	0	0	0	0	1	0	1	0	0
1	0	19	1	0	0	18	1	1	0	1	0	1	0	0	0	0	0	0	1	0	1	0	0
1	0	19	1	0	0	18	1	1	0	1	0	1	0	0	0	0	0	0	1	0	1	0	0
1	0	19	1	0	0	18	1	1	1	1	0	0	1	0	0	1	0	0	0	0	1	0	0
1	0	19	1	0	0	18	2	1	0	1	0	0	0	1	0	0	0	0	1	0	1	0	0
1	0	19	1	0	0	18	2	1	1	1	0	1	0	0	0	1	0	0	0	0	0	1	0
1	0	20	1	0	0	18	2	1	0	0	0	1	0	0	0	0	0	0	1	0	0	0	1
1	0	20	1	0	0	18	1	1	0	1	0	1	0	0	0	0	0	0	1	0	0	1	0
1	0	20	1	0	0	18	1	1	1	1	0	0	0	1	0	1	0	0	0	0	1	0	0
1	0	20	0	0	1	18	4	1	0	1	0	1	0	0	0	0	0	0	1	1	0	0	0
1	0	21	1	0	0	18	1	1	0	0	0	1	0	0	0	0	0	0	1	0	0	0	1
1	0	21	1	0	0	18	2	1	0	0	0	1	0	0	0	0	0	0	1	0	0	0	1
1	0	21	1	0	0	18	2	1	0	0	0	0	0	1	0	0	0	0	1	0	0	0	1
1	0	21	1	0	0	18	1	1	0	1	0	1	0	0	0	0	0	0	1	1	0	0	0
1	0	21	1	0	0	18	1	1	0	1	0	0	0	1	0	0	0	0	1	0	1	0	0
1	0	22	1	0	0	18	1	1	0	0	0	0	0	1	0	0	0	0	1	0	0	0	1
1	0	22	1	0	0	18	4	1	0	0	0	0	0	1	0	0	0	0	1	0	0	0	1
1	0	22	1	0	0	18	1	1	0	1	0	0	0	1	0	0	0	0	1	0	1	0	0
1	0	22	1	0	0	18	1	1	0	1	0	0	0	1	0	0	0	0	1	0	1	0	0
1	0	23	0	0	1	18	4	1	0	0	0	1	0	0	0	0	0	0	1	0	0	0	1
1	0	28	1	0	0	18	3	1	0	1	0	1	0	0	0	0	0	0	1	0	1	0	0
1	0	21	0	1	0	18	1	1	0	0	0	0	0	1	0	0	0	0	1	0	0	0	1
1	0	18	1	0	0	18	1	1	0	0	0	0	0	1	0	0	0	0	1	0	0	0	1
1	0	18	1	0	0	18	1	1	0	0	0	1	0	0	0	0	0	0	1	0	0	0	1
1	0	19	1	0	0	18	2	1	0	0	0	1	0	0	0	0	0	0	1	0	0	0	1
1	0	19	1	0	0	18	2	1	0	0	0	1	0	0	0	0	0	0	1	0	0	0	1

1	0	19	1	0	0	18	2	1	0	1	0	1	0	0	0	0	0	0	1	0	1	0	0
1	0	20	1	0	0	18	4	1	0	0	0	1	0	0	0	0	0	0	1	0	0	0	1
1	0	18	1	0	0	18	2	1	0	0	0	1	0	0	0	0	0	0	1	0	0	0	1
1	0	18	1	0	0	18	2	1	0	0	0	0	0	1	0	0	0	0	1	0	0	0	1
1	0	18	1	0	0	18	1	1	0	1	0	1	0	0	0	0	0	0	1	0	1	0	0
1	0	18	0	0	1	18	2	1	0	1	0	1	0	0	0	0	0	0	1	0	1	0	0
1	0	18	0	0	1	18	2	1	0	1	0	0	0	1	0	0	0	0	1	0	1	0	0
1	0	18	1	0	0	18	1	1	0	0	0	1	0	0	0	0	0	0	1	0	0	0	1
1	0	18	1	0	0	18	1	1	0	0	0	1	0	0	0	0	0	0	1	0	0	0	1
1	0	18	1	0	0	18	1	1	0	1	0	1	0	0	0	0	0	0	1	0	1	0	0
1	0	19	1	0	0	18	1	1	0	0	0	1	0	0	0	0	0	0	1	0	0	0	1
1	0	19	1	0	0	18	1	1	0	0	0	1	0	0	0	0	0	0	1	0	0	0	1
1	0	19	1	0	0	18	1	1	0	1	0	0	0	1	0	0	0	0	1	0	1	0	0
1	0	19	1	0	0	18	1	1	0	1	0	0	0	1	0	0	0	0	1	0	0	1	0
1	0	19	1	0	0	18	1	1	0	1	0	1	0	0	0	0	0	0	1	0	1	0	0
1	0	19	1	0	0	18	2	1	0	1	0	1	0	0	0	0	0	0	1	1	0	0	0
1	0	20	1	0	0	18	1	1	0	0	0	1	0	0	0	0	0	0	1	0	0	0	1
1	0	20	1	0	0	18	2	1	0	0	0	1	0	0	0	0	0	0	1	0	0	0	1
1	0	20	1	0	0	18	2	1	0	0	0	1	0	0	0	0	0	0	1	0	0	0	1
1	0	20	1	0	0	18	3	1	0	0	0	1	0	0	0	0	0	0	1	0	0	0	1
1	0	21	1	0	0	18	2	1	0	0	0	1	0	0	0	0	0	0	1	0	0	0	1
1	0	18	1	0	0	18	1	1	0	0	0	1	0	0	0	0	0	0	1	0	0	0	1
1	0	18	1	0	0	18	1	1	0	0	0	1	0	0	0	0	0	0	1	0	0	0	1
1	0	18	1	0	0	18	1	1	0	0	0	1	0	0	0	0	0	0	1	0	0	0	1
1	0	18	1	0	0	18	1	1	0	0	0	1	0	0	0	0	0	0	1	0	0	0	1
1	0	18	1	0	0	18	1	1	0	0	0	1	0	0	0	0	0	0	1	0	0	0	1
1	0	18	0	0	1	18	1	1	0	0	0	0	1	0	0	0	0	0	1	0	0	0	1
1	0	18	1	0	0	18	1	1	0	0	0	1	0	0	0	0	0	0	1	0	0	0	1
1	0	18	1	0	0	18	1	1	0	0	0	1	0	0	0	0	0	0	1	0	0	0	1
1	0	18	1	0	0	18	1	1	0	0	0	1	0	0	0	0	0	0	1	0	0	0	1
1	0	18	1	0	0	18	2	1	0	0	0	1	0	0	0	0	0	0	1	0	0	0	1
1	0	18	1	0	0	18	2	1	0	0	0	1	0	0	0	0	0	0	1	0	0	0	1
1	0	18	1	0	0	18	1	1	0	1	0	1	0	0	0	0	0	0	1	0	1	0	0
1	0	18	1	0	0	18	1	1	0	1	0	1	0	0	0	0	0	0	1	0	1	0	0
1	0	19	1	0	0	18	1	1	0	0	0	1	0	0	0	0	0	0	1	0	0	0	1
1	0	19	1	0	0	18	1	1	0	0	0	1	0	0	0	0	0	0	1	0	0	0	1
1	0	19	1	0	0	18	1	1	0	0	0	1	0	0	0	0	0	0	1	0	0	0	1
1	0	19	1	0	0	18	1	1	0	0	0	1	0	0	0	0	0	0	1	0	0	0	1
1	0	19	1	0	0	18	2	1	0	0	0	1	0	0	0	0	0	0	1	0	0	0	1
1	0	19	1	0	0	18	2	1	0	0	0	0	0	1	0	0	0	0	1	0	0	0	1
1	0	19	1	0	0	18	3	1	0	0	0	1	0	0	0	0	0	0	1	0	0	0	1
1	0	19	1	0	0	18	1	1	1	1	0	1	0	0	0	1	0	0	0	0	1	0	0
1	0	19	1	0	0	18	1	1	1	1	0	1	0	0	0	0	0	1	0	0	1	0	0
1	0	19	1	0	0	18	1	1	1	1	0	1	0	0	0	1	0	0	0	0	1	0	0
1	0	19	1	0	0	18	2	1	0	1	0	0	0	1	0	0	0	0	1	0	1	0	0
1	0	19	1	0	0	18	4	1	0	1	0	0	0	1	0	0	0	0	1	1	0	0	0
1	0	20	1	0	0	18	1	1	0	0	0	1	0	0	0	0	0	0	1	0	0	0	1
1	0	20	1	0	0	18	1	1	0	0	0	1	0	0	0	0	0	0	1	0	0	0	1
1	0	20	1	0	0	18	1	1	0	0	0	1	0	0	0	0	0	0	1	0	0	0	1
1	0	20	1	0	0	18	2	1	0	0	0	1	0	0	0	0	0	0	1	0	0	0	1

1	0	20	1	0	0	18	4	1	0	0	0	0	1	0	0	0	0	0	1	0	0	0	1
1	0	20	1	0	0	18	8	1	0	1	0	0	0	1	0	0	0	0	1	0	1	0	0
1	0	19	1	0	0	18	1	1	0	0	0	0	0	1	0	0	0	0	1	0	0	0	1
1	0	19	1	0	0	18	2	1	0	0	0	1	0	0	0	0	0	0	1	0	0	0	1
1	0	19	0	0	1	18	2	1	0	1	0	1	0	0	0	0	0	0	1	0	0	1	0
1	0	19	1	0	0	18	2	1	0	1	0	1	0	0	0	0	0	0	1	0	1	0	0
1	0	20	1	0	0	18	1	1	0	0	0	0	1	0	0	0	0	0	1	0	0	0	1
1	0	23	1	0	0	18	4	1	0	1	0	0	1	0	0	0	0	0	1	0	1	0	0
1	0	18	1	0	0	18	1	1	0	0	0	1	0	0	0	0	0	0	1	0	0	0	1
1	0	19	1	0	0	18	1	1	0	0	0	1	0	0	0	0	0	0	1	0	0	0	1
1	0	19	1	0	0	18	1	1	0	1	0	1	0	0	0	0	0	0	1	0	1	0	0
1	0	20	1	0	0	18	2	1	0	0	0	1	0	0	0	0	0	0	1	0	0	0	1
1	0	20	1	0	0	18	3	1	0	0	0	1	0	0	0	0	0	0	1	0	0	0	1
1	0	20	1	0	0	18	2	1	0	1	0	1	0	0	0	0	0	0	1	0	1	0	0
1	0	20	1	0	0	18	2	1	0	1	0	1	0	0	0	0	0	0	1	0	0	1	0
1	0	18	1	0	0	18	1	1	0	0	0	1	0	0	0	0	0	0	1	0	0	0	1
1	0	18	1	0	0	18	1	1	0	1	0	1	0	0	0	0	0	0	1	0	1	0	0
1	0	18	0	0	1	18	1	1	0	1	0	0	0	1	0	0	0	0	1	0	1	0	0
1	0	19	1	0	0	18	1	1	0	0	0	1	0	0	0	0	0	0	1	0	0	0	1
1	0	19	1	0	0	18	1	1	0	0	0	1	0	0	0	0	0	0	1	0	0	0	1
1	0	19	1	0	0	18	2	1	0	1	0	0	0	1	0	0	0	0	1	0	1	0	0
1	0	19	1	0	0	18	2	1	0	1	0	1	0	0	0	0	0	0	1	0	0	1	0
1	0	20	1	0	0	18	1	1	0	0	0	1	0	0	0	0	0	0	1	0	0	0	1
1	0	20	1	0	0	18	4	1	0	1	0	1	0	0	0	0	0	0	1	0	0	1	0
1	0	21	1	0	0	18	2	1	0	0	0	0	1	0	0	0	0	0	1	0	0	0	1
1	0	19	1	0	0	18	1	1	0	0	0	1	0	0	0	0	0	0	1	0	0	0	1
1	0	19	1	0	0	18	1	1	0	0	0	1	0	0	0	0	0	0	1	0	0	0	1
1	0	20	1	0	0	18	1	1	0	1	0	1	0	0	0	0	0	0	1	0	0	1	0
1	0	20	1	0	0	18	7	1	1	1	0	0	0	1	0	0	0	1	0	0	1	0	0
1	0	21	1	0	0	18	2	1	0	0	0	1	0	0	0	0	0	0	1	0	0	0	1
1	0	21	1	0	0	18	2	1	1	1	0	0	0	1	0	1	0	0	0	0	1	0	0
1	0	18	1	0	0	18	2	1	0	0	0	1	0	0	0	0	0	0	1	0	0	0	1
1	0	18	0	0	1	18	1	1	0	1	0	1	0	0	0	0	0	0	1	1	0	0	0
1	0	19	1	0	0	18	1	1	0	1	0	1	0	0	0	0	0	0	1	0	1	0	0
1	0	20	1	0	0	18	1	1	0	0	0	1	0	0	0	0	0	0	1	0	0	0	1
1	0	18	1	0	0	18	1	1	0	0	0	1	0	0	0	0	0	0	1	0	0	0	1
1	0	19	1	0	0	18	1	1	0	1	0	0	1	0	0	0	0	0	1	0	1	0	0
1	0	19	1	0	0	18	1	1	0	1	0	1	0	0	0	0	0	0	1	1	0	0	0
1	0	19	0	1	0	18	2	1	0	1	0	0	1	0	0	0	0	0	1	0	1	0	0
1	0	20	1	0	0	18	1	1	0	0	0	1	0	0	0	0	0	0	1	0	0	0	1
1	0	20	1	0	0	18	3	1	0	0	0	1	0	0	0	0	0	0	1	0	0	0	1
1	0	20	1	0	0	18	1	1	0	1	0	1	0	0	0	0	0	0	1	1	0	0	0
1	0	20	1	0	0	18	7	1	1	1	0	0	0	1	0	0	0	1	0	0	1	0	0
1	0	21	1	0	0	18	2	1	0	0	0	1	0	0	0	0	0	0	1	0	0	0	1
1	0	21	1	0	0	18	2	1	0	0	0	1	0	0	0	0	0	0	1	0	0	0	1
1	0	21	1	0	0	18	1	1	0	0	0	0	1	0	0	0	0	0	1	0	0	0	1
1	0	26	1	0	0	18	6	1	1	1	0	0	0	1	0	0	0	1	0	0	0	1	0
1	0	19	1	0	0	19	1	1	0	0	0	1	0	0	0	0	0	0	1	0	0	0	1
1	0	19	0	0	1	19	1	1	0	1	0	0	1	0	0	0	0	0	1	0	1	0	0
1	0	20	1	0	0	19	1	1	0	0	0	1	0	0	0	0	0	0	1	0	0	0	1

1	0	20	1	0	0	19	1	1	0	0	0	1	0	0	0	0	0	0	1	0	0	0	1
1	0	20	1	0	0	19	1	1	0	0	0	0	0	1	0	0	0	0	1	0	0	0	1
1	0	20	1	0	0	19	1	1	0	0	0	1	0	0	0	0	0	0	1	0	0	0	1
1	0	20	1	0	0	19	2	1	0	0	0	0	0	1	0	0	0	0	1	0	0	0	1
1	0	20	1	0	0	19	1	1	0	1	0	1	0	0	0	0	0	0	1	1	0	0	0
1	0	20	1	0	0	19	3	1	0	1	0	1	0	0	0	0	0	0	1	0	1	0	0
1	0	21	1	0	0	19	2	1	0	0	0	1	0	0	0	0	0	0	1	0	0	0	1
1	0	21	0	0	1	19	2	1	0	1	0	1	0	0	0	0	0	0	1	0	1	0	0
1	0	19	1	0	0	19	2	1	0	1	0	1	0	0	0	0	0	0	1	0	0	1	0
1	0	20	0	0	1	19	1	1	0	1	0	1	0	0	0	0	0	0	1	0	1	0	0
1	0	19	1	0	0	19	1	1	0	0	0	1	0	0	0	0	0	0	1	0	0	0	1
1	0	19	1	0	0	19	1	1	0	1	0	1	0	0	0	0	0	0	1	0	1	0	0
1	0	19	1	0	0	19	1	1	0	0	0	0	1	0	0	0	0	0	1	0	0	0	1
1	0	19	1	0	0	19	1	1	0	0	0	1	0	0	0	0	0	0	1	0	0	0	1
1	0	19	1	0	0	19	1	1	0	1	0	1	0	0	0	0	0	0	1	0	1	0	0
1	0	19	1	0	0	19	1	1	0	1	0	1	0	0	0	0	0	0	1	0	1	0	0
1	0	19	1	0	0	19	1	1	0	1	0	0	0	1	0	0	0	0	1	0	1	0	0
1	0	20	1	0	0	19	1	1	0	0	0	1	0	0	0	0	0	0	1	0	0	0	1
1	0	20	1	0	0	19	1	1	0	1	0	1	0	0	0	0	0	0	1	0	1	0	0
1	0	20	1	0	0	19	1	1	0	1	0	1	0	0	0	0	0	0	1	0	1	0	0
1	0	20	1	0	0	19	2	1	0	1	0	0	0	1	0	0	0	0	1	0	1	0	0
1	0	19	1	0	0	19	1	1	0	0	0	1	0	0	0	0	0	0	1	0	0	0	1
1	0	19	1	0	0	19	1	1	0	0	0	1	0	0	0	0	0	0	1	0	0	0	1
1	0	19	1	0	0	19	1	1	0	0	0	1	0	0	0	0	0	0	1	0	0	0	1
1	0	19	1	0	0	19	1	1	0	1	0	0	0	1	0	0	0	0	1	0	1	0	0
1	0	20	1	0	0	19	1	1	0	0	0	1	0	0	0	0	0	0	1	0	0	0	1
1	0	19	1	0	0	19	1	1	0	0	0	0	1	0	0	0	0	0	1	0	0	0	1
1	0	19	0	1	0	19	1	1	0	1	0	0	0	1	0	0	0	0	1	0	0	1	0
1	0	19	0	0	1	19	2	1	0	1	0	1	0	0	0	0	0	0	1	0	1	0	0
1	0	20	1	0	0	19	1	1	0	0	0	0	0	1	0	0	0	0	1	0	0	0	1
1	0	19	1	0	0	19	1	1	0	0	0	1	0	0	0	0	0	0	1	0	0	0	1
1	0	21	1	0	0	19	1	1	0	1	0	0	1	0	0	0	0	0	1	0	1	0	0
1	0	23	1	0	0	19	6	1	0	1	0	1	0	0	0	0	0	0	1	0	0	1	0
1	0	20	1	0	0	19	1	1	0	0	0	1	0	0	0	0	0	0	1	0	0	0	1
1	0	20	1	0	0	19	2	1	1	1	0	1	0	0	0	0	1	0	0	0	1	0	0
1	0	19	1	0	0	19	2	1	0	0	0	1	0	0	0	0	0	0	1	0	0	0	1
1	0	19	1	0	0	19	1	1	0	1	0	1	0	0	0	0	0	0	1	1	0	0	0
1	0	20	1	0	0	20	1	1	0	0	0	0	0	1	0	0	0	0	1	0	0	0	1
1	0	21	1	0	0	20	2	1	0	1	0	1	0	0	0	0	0	0	1	0	1	0	0
1	0	25	1	0	0	20	2	1	0	0	0	0	0	1	0	0	0	0	1	0	0	0	1
1	0	20	1	0	0	20	2	1	0	0	0	1	0	0	0	0	0	0	1	0	0	0	1
1	0	21	1	0	0	20	1	1	0	0	0	1	0	0	0	0	0	0	1	0	0	0	1
1	0	22	1	0	0	20	2	1	0	0	0	0	0	1	0	0	0	0	1	0	0	0	1
1	0	20	1	0	0	20	1	1	0	1	0	1	0	0	0	0	0	0	1	1	0	0	0
1	0	22	1	0	0	21	1	1	0	0	0	1	0	0	0	0	0	0	1	0	0	0	1
1	0	23	1	0	0	21	1	1	0	1	0	1	0	0	0	0	0	0	1	1	0	0	0
1	0	26	1	0	0	21	1	1	0	0	0	1	0	0	0	0	0	0	1	0	0	0	1
1	0	21	1	0	0	21	1	1	0	0	0	1	0	0	0	0	0	0	1	0	0	0	1
1	0	25	0	1	0	22	1	1	0	0	0	1	0	0	0	0	0	0	1	0	0	0	1
1	0	24	1	0	0	22	1	1	0	1	0	1	0	0	0	0	0	0	1	1	0	0	0

1	0	22	1	0	0	22	1	1	0	0	0	1	0	0	0	0	0	0	1	0	0	0	1
1	0	25	1	0	0	24	1	1	0	0	0	1	0	0	0	0	0	0	1	0	0	0	1
0	1	18	1	0	0	0	0	0	0	0	1	0	0	0	1	0	0	0	1	0	0	0	1
0	1	18	1	0	0	0	0	0	0	0	1	0	0	0	1	0	0	0	1	0	0	0	1
0	1	18	1	0	0	0	0	0	0	0	1	0	0	0	1	0	0	0	1	0	0	0	1
0	1	18	1	0	0	0	0	0	0	0	1	0	0	0	1	0	0	0	1	0	0	0	1
0	1	18	0	0	1	0	0	0	0	0	1	0	0	0	1	0	0	0	1	0	0	0	1
0	1	18	1	0	0	0	0	0	0	0	1	0	0	0	1	0	0	0	1	0	0	0	1
0	1	18	1	0	0	0	0	0	0	0	1	0	0	0	1	0	0	0	1	0	0	0	1
0	1	18	1	0	0	0	0	0	0	0	1	0	0	0	1	0	0	0	1	0	0	0	1
0	1	18	0	1	0	0	0	0	0	0	1	0	0	0	1	0	0	0	1	0	0	0	1
0	1	18	1	0	0	0	0	0	0	0	1	0	0	0	1	0	0	0	1	0	0	0	1
0	1	18	1	0	0	0	0	0	0	0	1	0	0	0	1	0	0	0	1	0	0	0	1
0	1	18	1	0	0	0	0	0	0	0	1	0	0	0	1	0	0	0	1	0	0	0	1
0	1	18	1	0	0	0	0	0	0	0	1	0	0	0	1	0	0	0	1	0	0	0	1
0	1	18	0	0	1	0	0	0	0	0	1	0	0	0	1	0	0	0	1	0	0	0	1
0	1	18	1	0	0	0	0	0	0	0	1	0	0	0	1	0	0	0	1	0	0	0	1
0	1	18	1	0	0	0	0	0	0	0	1	0	0	0	1	0	0	0	1	0	0	0	1
0	1	18	1	0	0	0	0	0	0	0	1	0	0	0	1	0	0	0	1	0	0	0	1
0	1	18	1	0	0	0	0	0	0	0	1	0	0	0	1	0	0	0	1	0	0	0	1
0	1	18	1	0	0	0	0	0	0	0	1	0	0	0	1	0	0	0	1	0	0	0	1
0	1	18	1	0	0	0	0	0	0	0	1	0	0	0	1	0	0	0	1	0	0	0	1
0	1	18	1	0	0	0	0	0	0	0	1	0	0	0	1	0	0	0	1	0	0	0	1
0	1	18	1	0	0	0	0	0	0	0	1	0	0	0	1	0	0	0	1	0	0	0	1
0	1	18	1	0	0	0	0	0	0	0	1	0	0	0	1	0	0	0	1	0	0	0	1
0	1	18	1	0	0	0	0	0	0	0	1	0	0	0	1	0	0	0	1	0	0	0	1
0	1	18	1	0	0	0	0	0	0	0	1	0	0	0	1	0	0	0	1	0	0	0	1
0	1	18	1	0	0	0	0	0	0	0	1	0	0	0	1	0	0	0	1	0	0	0	1
0	1	18	1	0	0	0	0	0	0	0	1	0	0	0	1	0	0	0	1	0	0	0	1
0	1	18	1	0	0	0	0	0	0	0	1	0	0	0	1	0	0	0	1	0	0	0	1
0	1	18	0	1	0	0	0	0	0	0	1	0	0	0	1	0	0	0	1	0	0	0	1
0	1	18	1	0	0	0	0	0	0	0	1	0	0	0	1	0	0	0	1	0	0	0	1
0	1	18	1	0	0	0	0	0	0	0	1	0	0	0	1	0	0	0	1	0	0	0	1
0	1	18	1	0	0	0	0	0	0	0	1	0	0	0	1	0	0	0	1	0	0	0	1
0	1	18	1	0	0	0	0	0	0	0	1	0	0	0	1	0	0	0	1	0	0	0	1
0	1	18	1	0	0	0	0	0	0	0	1	0	0	0	1	0	0	0	1	0	0	0	1
0	1	18	1	0	0	0	0	0	0	0	1	0	0	0	1	0	0	0	1	0	0	0	1
0	1	18	1	0	0	0	0	0	0	0	1	0	0	0	1	0	0	0	1	0	0	0	1
0	1	18	1	0	0	0	0	0	0	0	1	0	0	0	1	0	0	0	1	0	0	0	1
0	1	18	1	0	0	0	0	0	0	0	1	0	0	0	1	0	0	0	1	0	0	0	1
0	1	18	1	0	0	0	0	0	0	0	1	0	0	0	1	0	0	0	1	0	0	0	1
0	1	18	1	0	0	0	0	0	0	0	1	0	0	0	1	0	0	0	1	0	0	0	1
0	1	18	1	0	0	0	0	0	0	0	1	0	0	0	1	0	0	0	1	0	0	0	1
0	1	18	1	0	0	0	0	0	0	0	1	0	0	0	1	0	0	0	1	0	0	0	1
0	1	18	1	0	0	0	0	0	0	0	1	0	0	0	1	0	0	0	1	0	0	0	1
0	1	18	1	0	0	0	0	0	0	0	1	0	0	0	1	0	0	0	1	0	0	0	1
0	1	18	1	0	0	0	0	0	0	0	1	0	0	0	1	0	0	0	1	0	0	0	1
0	1	18	1	0	0	0	0	0	0	0	1	0	0	0	1	0	0	0	1	0	0	0	1
0	1	18	1	0	0	0	0	0	0	0	1	0	0	0	1	0	0	0	1	0	0	0	1
0	1	18	1	0	0	0	0	0	0	0	1	0	0	0	1	0	0	0	1	0	0	0	1

0	1	18	1	0	0	0	0	0	0	0	1	0	0	0	1	0	0	0	1	0	0	0	1
0	1	18	1	0	0	0	0	0	0	0	1	0	0	0	1	0	0	0	1	0	0	0	1
0	1	18	1	0	0	0	0	0	0	0	1	0	0	0	1	0	0	0	1	0	0	0	1
0	1	18	1	0	0	0	0	0	0	0	1	0	0	0	1	0	0	0	1	0	0	0	1
0	1	18	1	0	0	0	0	0	0	0	1	0	0	0	1	0	0	0	1	0	0	0	1
0	1	18	1	0	0	0	0	0	0	0	1	0	0	0	1	0	0	0	1	0	0	0	1
0	1	18	1	0	0	0	0	0	0	0	1	0	0	0	1	0	0	0	1	0	0	0	1
0	1	18	1	0	0	0	0	0	0	0	1	0	0	0	1	0	0	0	1	0	0	0	1
0	1	18	1	0	0	0	0	0	0	0	1	0	0	0	1	0	0	0	1	0	0	0	1
0	1	18	1	0	0	0	0	0	0	0	1	0	0	0	1	0	0	0	1	0	0	0	1
0	1	18	1	0	0	0	0	0	0	0	1	0	0	0	1	0	0	0	1	0	0	0	1
0	1	18	1	0	0	0	0	0	0	0	1	0	0	0	1	0	0	0	1	0	0	0	1
0	1	18	1	0	0	0	0	0	0	0	1	0	0	0	1	0	0	0	1	0	0	0	1
0	1	18	1	0	0	0	0	0	0	0	1	0	0	0	1	0	0	0	1	0	0	0	1
0	1	18	1	0	0	0	0	0	0	0	1	0	0	0	1	0	0	0	1	0	0	0	1
0	1	18	1	0	0	0	0	0	0	0	1	0	0	0	1	0	0	0	1	0	0	0	1
0	1	18	0	0	1	0	0	0	0	0	1	0	0	0	1	0	0	0	1	0	0	0	1
0	1	18	1	0	0	0	0	0	0	0	1	0	0	0	1	0	0	0	1	0	0	0	1
0	1	18	0	1	0	0	0	0	0	0	1	0	0	0	1	0	0	0	1	0	0	0	1
0	1	18	1	0	0	0	0	0	0	0	1	0	0	0	1	0	0	0	1	0	1	0	0
0	1	19	1	0	0	0	0	0	0	0	1	0	0	0	1	0	0	0	1	0	0	0	1
0	1	19	1	0	0	0	0	0	0	0	1	0	0	0	1	0	0	0	1	0	0	0	1
0	1	19	1	0	0	0	0	0	0	0	1	0	0	0	1	0	0	0	1	0	0	0	1
0	1	19	1	0	0	0	0	0	0	0	1	0	0	0	1	0	0	0	1	0	0	0	1
0	1	19	1	0	0	0	0	0	0	0	1	0	0	0	1	0	0	0	1	0	0	0	1
0	1	19	1	0	0	0	0	0	0	0	1	0	0	0	1	0	0	0	1	0	0	0	1
0	1	19	1	0	0	0	0	0	0	0	1	0	0	0	1	0	0	0	1	0	0	0	1
0	1	19	1	0	0	0	0	0	0	0	1	0	0	0	1	0	0	0	1	0	0	0	1
0	1	19	1	0	0	0	0	0	0	0	1	0	0	0	1	0	0	0	1	0	0	0	1
0	1	19	1	0	0	0	0	0	0	0	1	0	0	0	1	0	0	0	1	0	0	0	1
0	1	19	1	0	0	0	0	0	0	0	1	0	0	0	1	0	0	0	1	0	0	0	1
0	1	19	1	0	0	0	0	0	0	0	1	0	0	0	1	0	0	0	1	0	0	0	1
0	1	19	1	0	0	0	0	0	0	0	1	0	0	0	1	0	0	0	1	0	0	0	1
0	1	19	1	0	0	0	0	0	0	0	1	0	0	0	1	0	0	0	1	0	0	0	1
0	1	19	1	0	0	0	0	0	0	0	1	0	0	0	1	0	0	0	1	0	0	0	1
0	1	19	0	1	0	0	0	0	0	0	1	0	0	0	1	0	0	0	1	0	0	0	1
0	1	19	1	0	0	0	0	0	0	0	1	0	0	0	1	0	0	0	1	0	0	0	1
0	1	19	1	0	0	0	0	0	0	0	1	0	0	0	1	0	0	0	1	0	0	0	1
0	1	19	1	0	0	0	0	0	0	0	1	0	0	0	1	0	0	0	1	0	0	0	1
0	1	19	1	0	0	0	0	0	0	0	1	0	0	0	1	0	0	0	1	0	0	0	1
0	1	19	1	0	0	0	0	0	0	0	1	0	0	0	1	0	0	0	1	0	0	0	1
0	1	19	1	0	0	0	0	0	0	0	1	0	0	0	1	0	0	0	1	0	0	0	1
0	1	19	1	0	0	0	0	0	0	0	1	0	0	0	1	0	0	0	1	0	0	0	1
0	1	19	1	0	0	0	0	0	0	0	1	0	0	0	1	0	0	0	1	0	0	0	1
0	1	19	1	0	0	0	0	0	0	0	1	0	0	0	1	0	0	0	1	0	0	0	1
0	1	19	1	0	0	0	0	0	0	0	1	0	0	0	1	0	0	0	1	0	0	0	1
0	1	19	1	0	0	0	0	0	0	0	1	0	0	0	1	0	0	0	1	0	0	0	1
0	1	19	1	0	0	0	0	0	0	0	1	0	0	0	1	0	0	0	1	0	0	0	1
0	1	19	0	1	0	0	0	0	0	0	1	0	0	0	1	0	0	0	1	0	0	0	1
0	1	19	1	0	0	0	0	0	0	0	1	0	0	0	1	0	0	0	1	0	0	0	1

0	1	19	1	0	0	0	0	0	0	0	1	0	0	0	1	0	0	0	1	0	0	0	1
0	1	19	1	0	0	0	0	0	0	0	1	0	0	0	1	0	0	0	1	0	0	0	1
0	1	19	1	0	0	0	0	0	0	0	1	0	0	0	1	0	0	0	1	0	0	0	1
0	1	19	1	0	0	0	0	0	0	0	1	0	0	0	1	0	0	0	1	0	0	0	1
0	1	19	0	0	1	0	0	0	0	0	1	0	0	0	1	0	0	0	1	0	0	0	1
0	1	19	1	0	0	0	0	0	0	0	1	0	0	0	1	0	0	0	1	0	0	0	1
0	1	19	1	0	0	0	0	0	0	0	1	0	0	0	1	0	0	0	1	0	0	0	1
0	1	19	1	0	0	0	0	0	0	0	1	0	0	0	1	0	0	0	1	0	0	0	1
0	1	19	1	0	0	0	0	0	0	0	1	0	0	0	1	0	0	0	1	0	0	0	1
0	1	19	1	0	0	0	0	0	0	0	1	0	0	0	1	0	0	0	1	0	0	0	1
0	1	19	1	0	0	0	0	0	0	0	1	0	0	0	1	0	0	0	1	0	0	0	1
0	1	19	1	0	0	0	0	0	0	0	1	0	0	0	1	0	0	0	1	0	0	0	1
0	1	19	1	0	0	0	0	0	0	0	1	0	0	0	1	0	0	0	1	0	0	0	1
0	1	19	1	0	0	0	0	0	0	0	1	0	0	0	1	0	0	0	1	0	0	0	1
0	1	19	1	0	0	0	0	0	0	0	1	0	0	0	1	0	0	0	1	0	0	0	1
0	1	19	1	0	0	0	0	0	0	0	1	0	0	0	1	0	0	0	1	0	0	0	1
0	1	19	1	0	0	0	0	0	0	0	1	0	0	0	1	0	0	0	1	0	0	0	1
0	1	19	1	0	0	0	0	0	0	0	1	0	0	0	1	0	0	0	1	0	0	0	1
0	1	19	1	0	0	0	0	0	0	0	1	0	0	0	1	0	0	0	1	1	0	0	0
0	1	20	1	0	0	0	0	0	0	0	1	0	0	0	1	0	0	0	1	0	0	0	1
0	1	20	0	1	0	0	0	0	0	0	1	0	0	0	1	0	0	0	1	0	0	0	1
0	1	20	1	0	0	0	0	0	0	0	1	0	0	0	1	0	0	0	1	0	0	0	1
0	1	20	1	0	0	0	0	0	0	0	1	0	0	0	1	0	0	0	1	0	0	0	1
0	1	20	1	0	0	0	0	0	0	0	1	0	0	0	1	0	0	0	1	0	0	0	1
0	1	20	1	0	0	0	0	0	0	0	1	0	0	0	1	0	0	0	1	0	0	0	1
0	1	20	1	0	0	0	0	0	0	0	1	0	0	0	1	0	0	0	1	0	0	0	1
0	1	20	1	0	0	0	0	0	0	0	1	0	0	0	1	0	0	0	1	0	0	0	1
0	1	21	1	0	0	0	0	0	0	0	1	0	0	0	1	0	0	0	1	0	0	0	1
0	1	23	1	0	0	0	0	0	0	0	1	0	0	0	1	0	0	0	1	0	0	0	1
0	1	18	1	0	0	12	5	1	0	1	0	0	1	0	0	0	0	0	1	0	1	0	0
0	1	18	0	1	0	12	25	1	1	1	0	1	0	0	0	1	0	0	0	0	0	1	0
0	1	24	0	1	0	12	12	1	1	1	0	1	0	0	0	1	0	0	0	0	1	0	0
0	1	18	1	0	0	13	6	1	1	1	0	0	0	1	0	0	0	1	0	0	0	0	1
0	1	18	1	0	0	13	17	1	0	1	0	0	0	1	0	0	0	0	1	0	1	0	0
0	1	20	0	1	0	13	3	0	1	1	0	1	0	0	0	1	0	0	0	0	1	0	0
0	1	22	1	0	0	13	11	1	0	0	0	1	0	0	0	0	0	0	1	0	0	0	1
0	1	18	1	0	0	14	4	1	0	0	0	1	0	0	0	0	0	0	1	0	0	0	1
0	1	18	1	0	0	14	5	1	0	0	0	1	0	0	0	0	0	0	1	0	0	0	1
0	1	18	1	0	0	14	3	1	1	1	0	0	0	1	0	0	0	1	0	0	1	0	0
0	1	18	1	0	0	14	5	1	1	1	0	1	0	0	0	1	0	0	0	1	0	0	0
0	1	18	1	0	0	14	30	1	0	1	0	0	0	1	0	0	0	0	1	0	1	0	0
0	1	19	1	0	0	14	1	1	0	0	0	1	0	0	0	0	0	0	1	0	0	0	1
0	1	19	0	1	0	14	10	0	1	0	0	0	0	0	1	0	0	1	0	0	0	0	1
0	1	19	1	0	0	14	5	1	0	1	0	1	0	0	0	0	0	0	1	0	1	0	0
0	1	19	1	0	0	14	8	1	1	1	0	0	0	1	0	0	1	0	0	0	1	0	0
0	1	20	1	0	0	14	4	1	0	0	0	1	0	0	0	0	0	0	1	0	0	0	1
0	1	20	1	0	0	14	9	1	0	0	0	1	0	0	0	0	0	0	1	0	0	0	1
0	1	20	1	0	0	14	5	1	0	1	0	1	0	0	0	0	0	0	1	0	1	0	0
0	1	20	1	0	0	14	5	1	0	1	0	1	0	0	0	0	0	0	1	1	0	0	0
0	1	20	1	0	0	14	5	1	0	1	0	1	0	0	0	0	0	0	1	0	0	1	0

0	1	20	1	0	0	14	6	1	0	1	0	1	0	0	0	0	0	0	1	0	0	1	0
0	1	22	1	0	0	14	5	1	0	1	0	1	0	0	0	0	0	0	1	0	1	0	0
0	1	27	1	0	0	14	9	1	0	1	0	1	0	0	0	0	0	0	1	1	0	0	0
0	1	18	1	0	0	15	2	1	0	0	0	1	0	0	0	0	0	0	1	0	0	0	1
0	1	18	1	0	0	15	4	1	0	0	0	1	0	0	0	0	0	0	1	0	0	0	1
0	1	18	1	0	0	15	5	1	0	0	0	1	0	0	0	0	0	0	1	0	0	0	1
0	1	18	1	0	0	15	1	1	0	1	0	1	0	0	0	0	0	0	1	0	1	0	0
0	1	18	1	0	0	15	1	1	1	1	0	1	0	0	0	1	0	0	0	0	1	0	0
0	1	18	1	0	0	15	2	1	0	1	0	1	0	0	0	0	0	0	1	0	1	0	0
0	1	18	1	0	0	15	2	1	1	1	0	1	0	0	0	1	0	0	0	0	0	1	0
0	1	18	0	1	0	15	2	0	1	1	0	0	0	0	1	0	0	1	0	0	1	0	0
0	1	18	1	0	0	15	2	1	0	1	0	1	0	0	0	0	0	0	1	0	1	0	0
0	1	18	1	0	0	15	4	1	1	1	0	1	0	0	0	1	0	0	0	0	1	0	0
0	1	18	1	0	0	15	4	1	1	1	0	1	0	0	0	1	0	0	0	0	1	0	0
0	1	18	1	0	0	15	6	1	1	1	0	1	0	0	0	1	0	0	0	0	1	0	0
0	1	18	1	0	0	15	6	1	1	1	0	1	0	0	0	1	0	0	0	1	0	0	0
0	1	18	1	0	0	15	6	1	1	1	0	1	0	0	0	1	0	0	0	0	0	1	0
0	1	18	0	1	0	15	10	0	1	1	0	0	0	0	1	0	0	1	0	0	1	0	0
0	1	18	1	0	0	15	12	1	1	1	0	0	0	1	0	0	0	1	0	0	1	0	0
0	1	18	0	1	0	15	15	0	1	1	0	0	0	0	1	0	0	1	0	0	1	0	0
0	1	19	1	0	0	15	1	1	0	0	0	1	0	0	0	0	0	0	1	0	0	0	1
0	1	19	1	0	0	15	2	1	0	0	0	1	0	0	0	0	0	0	1	1	0	0	0
0	1	19	1	0	0	15	3	1	0	0	0	1	0	0	0	0	0	0	1	0	0	0	1
0	1	19	1	0	0	15	3	1	0	0	0	0	0	1	0	0	0	0	1	0	0	0	1
0	1	19	1	0	0	15	3	1	0	0	0	1	0	0	0	0	0	0	1	0	0	0	1
0	1	19	1	0	0	15	5	1	0	0	0	1	0	0	0	0	0	0	1	0	0	0	1
0	1	19	1	0	0	15	5	1	0	0	0	1	0	0	0	0	0	0	1	0	0	0	1
0	1	19	1	0	0	15	6	1	0	0	0	1	0	0	0	0	0	0	1	0	0	0	1
0	1	19	1	0	0	15	7	1	0	0	0	1	0	0	0	0	0	0	1	0	0	0	1
0	1	19	1	0	0	15	1	1	0	1	0	1	0	0	0	0	0	0	1	1	0	0	0
0	1	19	1	0	0	15	2	1	0	1	0	1	0	0	0	0	0	0	1	0	1	0	0
0	1	19	1	0	0	15	3	1	0	1	0	1	0	0	0	0	0	0	1	0	1	0	0
0	1	19	0	1	0	15	3	0	1	1	0	0	0	0	1	1	0	0	0	0	0	1	0
0	1	19	1	0	0	15	3	1	1	1	0	1	0	0	0	1	0	0	0	0	1	0	0
0	1	19	1	0	0	15	4	1	0	1	0	1	0	0	0	0	0	0	1	0	1	0	0
0	1	19	1	0	0	15	4	1	1	1	0	0	1	0	0	0	1	0	0	0	1	0	0
0	1	19	1	0	0	15	5	1	1	1	0	1	0	0	0	0	0	1	0	0	1	0	0
0	1	19	1	0	0	15	5	1	0	1	0	1	0	0	0	0	0	0	1	1	0	0	0
0	1	19	1	0	0	15	5	1	0	1	0	1	0	0	0	0	0	0	1	0	0	1	0
0	1	19	1	0	0	15	8	1	0	1	0	1	0	0	0	0	0	0	1	0	1	0	0
0	1	20	1	0	0	15	2	1	0	0	0	1	0	0	0	0	0	0	1	0	0	0	1
0	1	20	1	0	0	15	2	1	0	1	0	1	0	0	0	0	0	0	1	0	1	0	0
0	1	20	1	0	0	15	4	1	0	1	0	1	0	0	0	0	0	0	1	0	1	0	0
0	1	20	1	0	0	15	5	1	1	1	0	0	0	1	0	1	0	0	0	0	1	0	0
0	1	20	1	0	0	15	7	1	0	1	0	1	0	0	0	0	0	0	1	1	0	0	0
0	1	20	1	0	0	15	8	1	0	1	0	1	0	0	0	0	0	0	1	0	1	0	0
0	1	20	1	0	0	15	8	1	0	1	0	1	0	0	0	0	0	0	1	0	1	0	0
0	1	21	1	0	0	15	5	1	0	0	0	1	0	0	0	0	0	0	1	0	0	0	1
0	1	21	1	0	0	15	1	1	0	1	0	0	1	0	0	0	0	0	1	0	1	0	0
0	1	21	1	0	0	15	4	1	0	1	0	1	0	0	0	0	0	0	1	1	0	0	0

0	1	21	0	1	0	15	5	0	1	1	0	0	0	0	1	1	0	0	0	0	0	1	0
0	1	21	1	0	0	15	5	1	1	1	0	1	0	0	0	1	0	0	0	0	0	1	0
0	1	21	1	0	0	15	23	1	0	1	0	1	0	0	0	0	0	0	1	0	1	0	0
0	1	22	1	0	0	15	4	1	1	1	0	0	0	1	0	1	0	0	0	0	1	0	0
0	1	27	0	1	0	15	6	0	1	1	0	0	0	0	1	0	0	1	0	0	0	1	0
0	1	18	1	0	0	16	1	1	0	0	0	1	0	0	0	0	0	0	1	0	0	0	1
0	1	18	1	0	0	16	1	1	0	0	0	1	0	0	0	0	0	0	1	0	0	0	1
0	1	18	1	0	0	16	1	1	0	0	0	1	0	0	0	0	0	0	1	0	0	0	1
0	1	18	1	0	0	16	2	1	0	0	0	0	1	0	0	0	0	0	1	0	0	0	1
0	1	18	1	0	0	16	3	1	0	0	0	1	0	0	0	0	0	0	1	0	0	0	1
0	1	18	1	0	0	16	11	1	0	0	0	1	0	0	0	0	0	0	1	0	0	0	1
0	1	18	1	0	0	16	1	1	1	1	0	0	0	1	0	1	0	0	0	0	1	0	0
0	1	18	1	0	0	16	1	1	1	1	0	1	0	0	0	1	0	0	0	0	1	0	0
0	1	18	1	0	0	16	1	1	1	1	0	1	0	0	0	1	0	0	0	0	0	1	0
0	1	18	1	0	0	16	1	0	0	1	0	0	0	0	1	0	0	0	1	0	1	0	0
0	1	18	1	0	0	16	1	1	1	1	0	0	1	0	0	0	1	0	0	0	1	0	0
0	1	18	1	0	0	16	2	1	0	1	0	0	0	1	0	0	0	0	1	0	1	0	0
0	1	18	1	0	0	16	2	1	0	1	0	1	0	0	0	0	0	0	1	0	0	1	0
0	1	18	1	0	0	16	2	1	0	1	0	1	0	0	0	0	0	0	1	0	0	0	1
0	1	18	1	0	0	16	3	1	0	1	0	0	0	1	0	0	0	0	1	0	1	0	0
0	1	18	1	0	0	16	3	1	1	1	0	1	0	0	0	1	0	0	0	0	0	1	0
0	1	18	1	0	0	16	3	1	0	1	0	1	0	0	0	0	0	0	1	1	0	0	0
0	1	18	1	0	0	16	3	1	0	1	0	1	0	0	0	0	0	0	1	0	1	0	0
0	1	18	1	0	0	16	3	1	1	1	0	1	0	0	0	1	0	0	0	0	1	0	0
0	1	18	1	0	0	16	3	1	0	1	0	1	0	0	0	0	0	0	1	1	0	0	0
0	1	18	1	0	0	16	3	1	0	1	0	1	0	0	0	0	0	0	1	0	1	0	0
0	1	18	1	0	0	16	3	1	0	1	0	1	0	0	0	0	0	0	1	0	1	0	0
0	1	18	1	0	0	16	5	1	0	1	0	0	0	1	0	0	0	0	1	0	0	1	0
0	1	18	0	1	0	16	5	0	1	1	0	0	0	0	1	0	0	1	0	0	1	0	0
0	1	18	1	0	0	16	6	1	1	1	0	1	0	0	0	0	0	1	0	0	0	1	0
0	1	19	1	0	0	16	1	1	0	0	0	1	0	0	0	0	0	0	1	0	0	0	1
0	1	19	1	0	0	16	2	1	0	0	0	1	0	0	0	0	0	0	1	0	0	0	1
0	1	19	1	0	0	16	4	1	1	0	0	1	0	0	0	1	0	0	0	0	0	0	1
0	1	19	1	0	0	16	4	1	0	0	0	1	0	0	0	0	0	0	1	0	0	0	1
0	1	19	1	0	0	16	1	1	0	1	0	1	0	0	0	0	0	0	1	0	1	0	0
0	1	19	1	0	0	16	1	1	1	1	0	0	0	1	0	1	0	0	0	0	1	0	0
0	1	19	1	0	0	16	1	1	0	1	0	1	0	0	0	0	0	0	1	0	1	0	0
0	1	19	1	0	0	16	2	1	0	1	0	0	1	0	0	0	0	0	1	0	1	0	0
0	1	19	1	0	0	16	2	1	0	1	0	0	0	1	0	0	0	0	1	0	1	0	0
0	1	19	1	0	0	16	2	1	0	1	0	1	0	0	0	0	0	0	1	1	0	0	0
0	1	19	1	0	0	16	3	1	1	1	0	1	0	0	0	0	0	1	0	0	1	0	0
0	1	19	1	0	0	16	3	1	0	1	0	0	0	1	0	0	0	0	1	0	1	0	0
0	1	19	1	0	0	16	3	1	0	1	0	1	0	0	0	0	0	0	1	1	0	0	0
0	1	19	1	0	0	16	4	1	0	1	0	1	0	0	0	0	0	0	1	0	1	0	0
0	1	19	1	0	0	16	4	1	0	1	0	1	0	0	0	0	0	0	1	0	1	0	0
0	1	19	1	0	0	16	6	1	0	1	0	1	0	0	0	0	0	0	1	0	0	1	0
0	1	19	1	0	0	16	7	1	0	1	0	1	0	0	0	0	0	0	1	0	1	0	0
0	1	19	1	0	0	16	15	1	0	1	0	1	0	0	0	0	0	0	1	0	1	0	0
0	1	20	1	0	0	16	4	1	0	0	0	0	0	1	0	0	0	0	1	0	0	0	1
0	1	20	1	0	0	16	5	1	0	0	0	1	0	0	0	0	0	0	1	0	0	0	1

0	1	20	1	0	0	16	5	1	1	1	0	1	0	0	0	0	0	1	0	0	1	0	0
0	1	20	1	0	0	16	6	1	0	1	0	1	0	0	0	0	0	0	1	0	1	0	0
0	1	20	1	0	0	16	8	1	0	1	0	1	0	0	0	0	0	0	1	0	1	0	0
0	1	21	1	0	0	16	6	1	0	0	0	1	0	0	0	0	0	0	1	0	0	0	1
0	1	21	1	0	0	16	5	1	0	1	0	1	0	0	0	0	0	0	1	0	1	0	0
0	1	21	1	0	0	16	11	1	1	1	0	0	0	1	0	0	0	1	0	0	1	0	0
0	1	22	1	0	0	16	2	1	1	1	0	1	0	0	0	1	0	0	0	0	1	0	0
0	1	22	1	0	0	16	4	1	1	1	0	1	0	0	0	1	0	0	0	0	1	0	0
0	1	23	1	0	0	16	1	1	0	1	0	0	1	0	0	0	0	0	1	0	1	0	0
0	1	23	1	0	0	16	10	1	0	1	0	0	0	1	0	0	0	0	1	0	1	0	0
0	1	27	1	0	0	16	8	1	0	1	0	1	0	0	0	0	0	0	1	0	1	0	0
0	1	18	1	0	0	17	1	1	0	0	0	1	0	0	0	0	0	0	1	0	0	0	1
0	1	18	1	0	0	17	1	1	0	0	0	1	0	0	0	0	0	0	1	0	0	0	1
0	1	18	1	0	0	17	1	1	0	0	0	1	0	0	0	0	0	0	1	0	0	0	1
0	1	18	1	0	0	17	1	1	0	0	0	1	0	0	0	0	0	0	1	0	0	0	1
0	1	18	1	0	0	17	1	1	0	0	0	1	0	0	0	0	0	0	1	0	0	0	1
0	1	18	1	0	0	17	1	1	0	0	0	1	0	0	0	0	0	0	1	0	0	0	1
0	1	18	1	0	0	17	2	1	0	0	0	0	0	1	0	0	0	0	1	0	0	0	1
0	1	18	1	0	0	17	3	1	0	0	0	1	0	0	0	0	0	0	1	0	0	0	1
0	1	18	1	0	0	17	3	1	0	0	0	1	0	0	0	0	0	0	1	0	0	0	1
0	1	18	1	0	0	17	1	1	0	1	0	1	0	0	0	0	0	0	1	0	1	0	0
0	1	18	0	1	0	17	1	0	0	1	0	0	0	0	1	0	0	0	1	1	0	0	0
0	1	18	1	0	0	17	1	1	0	1	0	1	0	0	0	0	0	0	1	0	1	0	0
0	1	18	1	0	0	17	1	1	0	1	0	1	0	0	0	0	0	0	1	0	1	0	0
0	1	18	1	0	0	17	1	1	0	1	0	1	0	0	0	0	0	0	1	1	0	0	0
0	1	18	1	0	0	17	1	1	1	1	0	1	0	0	0	1	0	0	0	0	0	1	0
0	1	18	1	0	0	17	1	1	0	1	0	1	0	0	0	0	0	0	1	1	0	0	0
0	1	18	1	0	0	17	1	1	1	1	0	0	0	1	0	0	1	0	0	0	1	0	0
0	1	18	1	0	0	17	2	1	0	1	0	1	0	0	0	0	0	0	1	1	0	0	0
0	1	18	1	0	0	17	2	1	1	1	0	1	0	0	0	0	0	1	0	0	0	1	0
0	1	18	1	0	0	17	3	1	1	1	0	1	0	0	0	1	0	0	0	0	1	0	0
0	1	18	1	0	0	17	4	1	0	1	0	1	0	0	0	0	0	0	1	1	0	0	0
0	1	18	0	1	0	17	4	0	1	1	0	0	0	0	1	1	0	0	0	0	1	0	0
0	1	18	1	0	0	17	5	1	0	1	0	1	0	0	0	0	0	0	1	0	0	1	0
0	1	18	0	0	1	17	5	1	1	1	0	1	0	0	0	1	0	0	0	0	0	1	0
0	1	18	1	0	0	17	5	1	0	1	0	1	0	0	0	0	0	0	1	0	0	1	0
0	1	18	0	1	0	17	5	0	1	1	0	0	0	0	1	0	0	1	0	0	1	0	0
0	1	18	1	0	0	17	8	1	0	1	0	1	0	0	0	0	0	0	1	0	1	0	0
0	1	19	1	0	0	17	1	1	0	0	0	1	0	0	0	0	0	0	1	0	0	0	1
0	1	19	1	0	0	17	1	1	0	0	0	1	0	0	0	0	0	0	1	0	0	0	1
0	1	19	1	0	0	17	1	1	0	0	0	1	0	0	0	0	0	0	1	0	0	0	1
0	1	19	1	0	0	17	1	1	0	0	0	1	0	0	0	0	0	0	1	0	0	0	1
0	1	19	1	0	0	17	1	1	0	0	0	1	0	0	0	0	0	0	1	0	0	0	1
0	1	19	1	0	0	17	1	1	0	0	0	1	0	0	0	0	0	0	1	0	0	0	1
0	1	19	1	0	0	17	2	1	0	0	0	1	0	0	0	0	0	0	1	0	0	0	1
0	1	19	1	0	0	17	2	1	0	0	0	0	0	1	0	0	0	0	1	0	0	0	1
0	1	19	1	0	0	17	2	1	0	0	0	1	0	0	0	0	0	0	1	0	0	0	1
0	1	19	1	0	0	17	2	0	0	0	0	0	0	0	1	0	0	0	1	0	0	0	1
0	1	19	1	0	0	17	2	1	0	0	0	1	0	0	0	0	0	0	1	1	0	0	0
0	1	19	1	0	0	17	2	1	0	0	0	1	0	0	0	0	0	0	1	0	0	0	1

0	1	19	1	0	0	17	3	1	0	0	0	0	0	1	0	0	0	0	1	0	0	0	1
0	1	19	1	0	0	17	6	1	0	0	0	1	0	0	0	0	0	0	1	0	0	0	1
0	1	19	1	0	0	17	6	1	0	0	0	1	0	0	0	0	0	0	1	1	0	0	0
0	1	19	1	0	0	17	1	1	0	1	0	0	1	0	0	0	0	0	1	0	1	0	0
0	1	19	1	0	0	17	1	1	0	1	0	1	0	0	0	0	0	0	1	0	1	0	0
0	1	19	1	0	0	17	1	1	0	1	0	1	0	0	0	0	0	0	1	0	1	0	0
0	1	19	1	0	0	17	1	1	0	1	0	1	0	0	0	0	0	0	1	0	1	0	0
0	1	19	1	0	0	17	1	1	0	1	0	1	0	0	0	0	0	0	1	0	1	0	0
0	1	19	0	0	1	17	1	1	1	1	0	1	0	0	0	1	0	0	0	0	0	1	0
0	1	19	1	0	0	17	1	0	0	1	0	0	0	0	1	0	0	0	1	0	1	0	0
0	1	19	1	0	0	17	1	1	0	1	0	1	0	0	0	0	0	0	1	0	1	0	0
0	1	19	1	0	0	17	2	1	0	1	0	1	0	0	0	0	0	0	1	0	0	1	0
0	1	19	0	0	1	17	2	1	1	1	0	1	0	0	0	1	0	0	0	0	0	1	0
0	1	19	1	0	0	17	2	1	0	1	0	1	0	0	0	0	0	0	1	1	0	0	0
0	1	19	1	0	0	17	2	1	1	1	0	0	0	1	0	0	0	1	0	0	1	0	0
0	1	19	1	0	0	17	2	1	0	1	0	1	0	0	0	0	0	0	1	0	0	0	1
0	1	19	1	0	0	17	2	1	0	1	0	0	0	1	0	0	0	0	1	0	1	0	0
0	1	19	1	0	0	17	2	1	0	1	0	1	0	0	0	0	0	0	1	0	1	0	0
0	1	19	1	0	0	17	2	1	0	1	0	1	0	0	0	0	0	0	1	0	1	0	0
0	1	19	1	0	0	17	3	1	0	1	0	1	0	0	0	0	0	0	1	0	1	0	0
0	1	19	1	0	0	17	3	1	0	1	0	1	0	0	0	0	0	0	1	0	0	1	0
0	1	19	1	0	0	17	3	1	0	1	0	1	0	0	0	0	0	0	1	0	1	0	0
0	1	19	1	0	0	17	4	1	0	1	0	1	0	0	0	0	0	0	1	0	0	1	0
0	1	19	1	0	0	17	5	1	1	1	0	1	0	0	0	0	1	0	0	0	1	0	0
0	1	19	1	0	0	17	5	1	0	1	0	1	0	0	0	0	0	0	1	0	1	0	0
0	1	19	0	0	1	17	5	0	1	1	0	0	0	0	1	1	0	0	0	0	1	0	0
0	1	19	1	0	0	17	8	1	1	1	0	1	0	0	0	1	0	0	0	0	0	0	1
0	1	19	1	0	0	17	10	1	0	1	0	1	0	0	0	0	0	0	1	0	1	0	0
0	1	19	0	0	1	17	10	1	1	1	0	1	0	0	0	1	0	0	0	0	1	0	0
0	1	19	1	0	0	17	12	1	1	1	0	1	0	0	0	0	0	1	0	0	1	0	0
0	1	20	1	0	0	17	2	1	0	0	0	0	0	1	0	0	0	0	1	0	0	0	1
0	1	20	1	0	0	17	5	1	0	0	0	0	0	1	0	0	0	0	1	0	0	0	1
0	1	20	0	0	1	17	5	0	1	0	0	0	0	1	0	1	0	0	0	0	0	0	1
0	1	20	0	1	0	17	1	0	1	1	0	0	0	0	1	0	1	0	0	0	1	0	0
0	1	20	1	0	0	17	1	1	0	1	0	0	0	1	0	0	0	0	1	0	1	0	0
0	1	20	1	0	0	17	3	1	1	1	0	0	1	0	0	1	0	0	0	0	1	0	0
0	1	20	1	0	0	17	5	1	0	1	0	1	0	0	0	0	0	0	1	0	1	0	0
0	1	20	0	1	0	17	30	0	1	1	0	0	0	0	1	1	0	0	0	0	0	1	0
0	1	21	1	0	0	17	3	1	0	0	0	1	0	0	0	0	0	0	1	0	0	0	1
0	1	21	1	0	0	17	3	1	0	0	0	1	0	0	0	0	0	0	1	0	0	0	1
0	1	21	1	0	0	17	2	1	1	1	0	1	0	0	0	1	0	0	0	0	0	1	0
0	1	21	1	0	0	17	3	1	0	1	0	1	0	0	0	0	0	0	1	0	0	1	0
0	1	21	1	0	0	17	5	1	1	1	0	0	1	0	0	0	1	0	0	0	1	0	0
0	1	21	1	0	0	17	7	1	1	1	0	0	0	1	0	0	0	1	0	0	1	0	0
0	1	22	1	0	0	17	4	1	0	0	0	0	0	0	1	0	0	0	1	0	0	0	1
0	1	23	1	0	0	17	9	1	1	1	0	0	0	1	0	1	0	0	0	0	0	1	0
0	1	18	0	1	0	18	1	0	1	0	0	0	0	0	1	1	0	0	0	0	0	0	1
0	1	18	1	0	0	18	1	1	0	0	0	1	0	0	0	0	0	0	1	0	0	0	1
0	1	18	1	0	0	18	1	1	0	0	0	1	0	0	0	0	0	0	1	0	0	0	1
0	1	18	1	0	0	18	1	1	0	0	0	1	0	0	0	0	0	0	1	0	0	0	1

0	1	18	1	0	0	18	1	1	0	0	0	1	0	0	0	0	0	0	1	0	0	0	1
0	1	18	1	0	0	18	1	1	0	0	0	1	0	0	0	0	0	0	1	0	0	0	1
0	1	18	1	0	0	18	1	1	0	0	0	1	0	0	0	0	0	0	1	0	0	0	1
0	1	18	1	0	0	18	1	1	0	0	0	1	0	0	0	0	0	0	1	0	0	0	1
0	1	18	1	0	0	18	1	1	0	0	0	1	0	0	0	0	0	0	1	0	0	0	1
0	1	18	1	0	0	18	2	1	0	0	0	1	0	0	0	0	0	0	1	0	0	0	1
0	1	18	1	0	0	18	1	1	0	1	0	1	0	0	0	0	0	0	1	0	0	1	0
0	1	18	1	0	0	18	1	1	0	1	0	1	0	0	0	0	0	0	1	0	1	0	0
0	1	18	1	0	0	18	1	1	0	1	0	1	0	0	0	0	0	0	1	0	1	0	0
0	1	18	0	1	0	18	4	0	0	1	0	0	0	0	1	0	0	0	1	0	0	1	0
0	1	19	1	0	0	18	1	1	0	0	0	1	0	0	0	0	0	0	1	0	0	0	1
0	1	19	1	0	0	18	1	1	0	0	0	1	0	0	0	0	0	0	1	0	0	0	1
0	1	19	1	0	0	18	4	1	0	0	0	1	0	0	0	0	0	0	1	0	0	0	1
0	1	19	1	0	0	18	1	1	1	1	0	1	0	0	0	0	1	0	0	0	1	0	0
0	1	19	1	0	0	18	1	1	0	1	0	0	0	1	0	0	0	0	1	0	1	0	0
0	1	19	1	0	0	18	1	1	0	1	0	0	0	1	0	0	0	0	1	0	1	0	0
0	1	19	1	0	0	18	1	1	1	1	0	1	0	0	0	0	0	1	0	0	0	1	0
0	1	19	1	0	0	18	1	1	0	1	0	1	0	0	0	0	0	0	1	1	0	0	0
0	1	19	1	0	0	18	1	1	0	1	0	1	0	0	0	0	0	0	1	0	1	0	0
0	1	19	1	0	0	18	1	1	0	1	0	1	0	0	0	0	0	0	1	1	0	0	0
0	1	19	1	0	0	18	1	0	0	1	0	1	0	0	0	0	0	0	1	1	0	0	0
0	1	19	1	0	0	18	1	1	0	1	0	1	0	0	0	0	0	0	1	0	1	0	0
0	1	19	1	0	0	18	1	1	0	1	0	1	0	0	0	0	0	0	1	0	1	0	0
0	1	19	1	0	0	18	1	1	0	1	0	1	0	0	0	0	0	0	1	0	1	0	0
0	1	19	1	0	0	18	1	1	0	1	0	1	0	0	0	0	0	0	1	0	0	1	0
0	1	19	1	0	0	18	1	1	0	1	0	0	0	1	0	0	0	0	1	0	1	0	0
0	1	19	1	0	0	18	1	1	1	1	0	1	0	0	0	1	0	0	0	0	1	0	0
0	1	19	1	0	0	18	1	1	0	1	0	1	0	0	0	0	0	0	1	0	1	0	0
0	1	19	1	0	0	18	2	1	0	1	0	1	0	0	0	0	0	0	1	0	1	0	0
0	1	19	1	0	0	18	2	1	1	1	0	0	1	0	0	1	0	0	0	0	1	0	0
0	1	19	0	1	0	18	2	0	1	1	0	1	0	0	0	1	0	0	0	0	1	0	0
0	1	19	1	0	0	18	3	1	1	1	0	1	0	0	0	1	0	0	0	0	1	0	0
0	1	19	1	0	0	18	3	1	0	1	0	1	0	0	0	0	0	0	1	0	0	1	0
0	1	19	0	0	1	18	3	0	1	1	0	1	0	0	0	1	0	0	0	0	0	1	0
0	1	19	1	0	0	18	4	1	0	1	0	0	0	1	0	0	0	0	1	0	1	0	0
0	1	20	1	0	0	18	1	1	0	0	0	0	0	1	0	0	0	0	1	0	0	0	1
0	1	20	1	0	0	18	1	1	0	0	0	1	0	0	0	0	0	0	1	0	0	0	1
0	1	20	1	0	0	18	4	1	0	0	0	1	0	0	0	0	0	0	1	0	0	0	1
0	1	20	1	0	0	18	8	1	0	0	0	1	0	0	0	0	0	0	1	0	0	0	1
0	1	20	1	0	0	18	1	1	0	1	0	1	0	0	0	0	0	0	1	0	0	1	0
0	1	20	0	0	1	18	2	0	1	1	0	1	0	0	0	1	0	0	0	0	0	0	1
0	1	20	0	0	1	18	4	1	1	1	0	1	0	0	0	1	0	0	0	0	1	0	0
0	1	20	1	0	0	18	11	1	1	1	0	0	0	1	0	0	0	1	0	0	1	0	0
0	1	20	1	0	0	18	18	1	0	1	0	0	0	1	0	0	0	0	1	0	1	0	0
0	1	21	1	0	0	18	1	1	0	0	0	1	0	0	0	0	0	0	1	0	0	0	1
0	1	21	1	0	0	18	3	1	0	0	0	1	0	0	0	0	0	0	1	0	0	0	1
0	1	21	1	0	0	18	3	1	0	0	0	1	0	0	0	0	0	0	1	0	0	0	1
0	1	21	0	1	0	18	5	0	1	1	0	1	0	0	0	1	0	0	0	0	0	1	0
0	1	22	1	0	0	18	4	1	0	1	0	1	0	0	0	0	0	0	1	0	0	1	0
0	1	22	0	1	0	18	5	0	1	1	0	0	0	0	1	1	0	0	0	0	1	0	0

0	1	22	1	0	0	18	5	1	0	1	0	0	0	1	0	0	0	0	1	0	1	0	0
0	1	23	1	0	0	18	3	1	0	0	0	1	0	0	0	0	0	0	1	0	0	0	1
0	1	23	1	0	0	18	1	1	1	1	0	0	0	1	0	0	0	1	0	0	1	0	0
0	1	24	0	0	1	18	12	1	1	1	0	1	0	0	0	1	0	0	0	0	0	1	0
0	1	25	1	0	0	18	1	1	0	0	0	0	1	0	0	0	0	0	1	0	0	0	1
0	1	26	1	0	0	18	1	1	0	0	0	1	0	0	0	0	0	0	1	0	0	0	1
0	1	19	1	0	0	19	1	1	0	0	0	1	0	0	0	0	0	0	1	0	0	0	1
0	1	19	1	0	0	19	1	1	0	1	0	1	0	0	0	0	0	0	1	0	0	1	0
0	1	19	1	0	0	19	1	1	0	1	0	1	0	0	0	0	0	0	1	1	0	0	0
0	1	19	1	0	0	19	1	1	0	1	0	1	0	0	0	0	0	0	1	0	0	1	0
0	1	19	1	0	0	19	2	1	0	1	0	1	0	0	0	0	0	0	1	0	1	0	0
0	1	19	0	1	0	19	10	0	1	1	0	0	0	0	1	1	0	0	0	0	1	0	0
0	1	20	1	0	0	19	1	1	0	0	0	1	0	0	0	0	0	0	1	0	0	0	1
0	1	20	1	0	0	19	2	1	0	0	0	1	0	0	0	0	0	0	1	0	0	0	1
0	1	20	1	0	0	19	1	1	0	1	0	1	0	0	0	0	0	0	1	0	1	0	0
0	1	21	1	0	0	19	1	1	1	1	0	1	0	0	0	1	0	0	0	0	1	0	0
0	1	21	1	0	0	19	1	1	0	1	0	1	0	0	0	0	0	0	1	0	0	1	0
0	1	22	0	1	0	19	1	0	1	1	0	0	0	0	1	0	1	0	0	0	1	0	0
0	1	20	1	0	0	20	1	1	0	0	0	1	0	0	0	0	0	0	1	0	0	0	1
0	1	20	0	0	1	20	2	1	1	0	0	1	0	0	0	1	0	0	0	0	0	0	1
0	1	20	1	0	0	20	1	1	0	1	0	1	0	0	0	0	0	0	1	0	1	0	0
0	1	23	1	0	0	20	4	1	0	1	0	1	0	0	0	0	0	0	1	0	1	0	0

The number 1 represents the selected answer for single select options or multi-select options.
